# High-throughput Treg cell receptor sequencing reveals differential immune repertoires in rheumatoid arthritis with kidney deficiency

**DOI:** 10.7717/peerj.14837

**Published:** 2023-02-02

**Authors:** Lu Zhang, Wei Jiao, Hui Deng, Congqi Hu, Jia Xu, Jiahui Yu, Lijuan Liu, Mingying Zhang, Jiduo Liu, Guangxing Chen

**Affiliations:** 1 First Clinical Medical School, Guangzhou University of Chinese Medicine, Guangzhou, China; 2 Department of Rheumatology, First Affiliated Hospital of Guangzhou University of Chinese Medicine, Guangzhou, China; 3 Baiyun Hospital of the First Affiliated Hospital of Guangzhou University of Chinese Medicine, Guangzhou, China

**Keywords:** Rheumatoid arthritis, Regulatory T cell, Traditional Chinese medicine, Kidney deficiency

## Abstract

**Background:**

Regulatory T (Treg) cells are important immune cells that are regulated by adaptive immunity in the composition of Treg-cell subsets and T-cell receptors (TCRs). Treg cells are related to most autoimmune diseases, such as rheumatoid arthritis (RA). In traditional Chinese medicine (TCM), RA is typically attributed to kidney deficiency (KD) associated with the immunosenescence that causes immune dysfunction and the impaired function of Treg cells. So far, however, no mechanism related to KD and immune repertoires has been identified in RA.

**Methods:**

Flow cytometry and high-throughput Treg-cell receptor sequencing were used to investigate the amount of different Treg-cell subsets and the diversity of TCRs between RA patients and healthy subjects, as well as between KD RA and non-KD RA patients. RT-qPCR was used to validate the high-throughput sequencing results.

**Results:**

The data showed that the amount of naïve Treg cells in KD patients was less than in non-KD RA patients (*P* = 0.004) with no significant differences observed between other subsets. In the TCR of Treg cells, the length of complementarity determining region 3 (CDR3) was low and clonotypes increased in the KD group compared with the non-KD group. The diversity and abundance of Treg TCRs were low, as determined by the Hill number. In addition, several V(D)J combinations, such as T-cell receptor beta variable 7-2 (TRBV7-2), TRBV11-1, TRBV13, TRBV15, and TRBJ2-3, varied significantly between the two groups, indicating that KD causes Treg dysfunction. RT-qPCR shows that FOXP3 expression in peripheral blood Treg is lower in KD than in non-KD.

**Conclusion:**

The results demonstrate the close correlation between KD and immune repertoires in RA and provide a new evaluation method for RA in TCM.

## Introduction

Rheumatoid arthritis (RA) is a chronic autoimmune inflammatory disorder, defined as autoreactive T-cell accumulation and pro-inflammatory cytokine overproduction, with a high rate of disability that mainly influences the joints (Scherer, Häupl & Burmester, 2020).

The prevalence of RA is estimated to be 0.5–1.1% of the population in most developed countries, with conflicting data for developing countries (Wang et al., 2016). The etiology of RA is closely tied to adaptive and immune responses, with the interaction of genetic, environmental, and behavioral risk elements contributing to impaired immune tolerance and autoimmune processes (Tobón, Youinou & Saraux, 2010).

Traditional Chinese medicine (TCM) states that the kidney is one of the five Zang (viscera) organs of the body; these organs store the body’s essence (Petrovská et al., 2021). The kidney is essential to reproduction, growth, development, and aging (Efferth, Shan & Zhang, 2016; Zhao et al., 2015). A child’s growth indicates the strengthening of their essence, while the age-related weakening of the human body indicates essence consumption. The waning of kidney essence leads to senility, qi stagnation, blood stasis, and even joint stiffness. This can lead to rheumatoid arthritis, which TCM attributes directly to kidney deficiency (KD). Compared with non-KD RA patients, KD RA patients may experience worse pain and compounding immune disorders. One study found that kidney deficiency was closely connected with disease activity and autoimmune disorders in RA (Mo et al., 2016).

It is well known that cellular and humoral immune disorders are caused by an imbalance in the function or the number of T cells, and that T-cell-mediated immunity is essential for the pathogenesis of RA (Rao et al., 2017). CD4+ T cells interact with the peptide presented by the major histocompatibility complex (MHC) through specific T-cell receptors (TCRs) (Jiang et al., 2020). Treg cells are mainly generated through self antigen recognition in the thymus and through both self and foreign antigen recognition in peripheral lymphoid organs (Abbas, Lichtman & Pillai, 2014). Treg cells have recently been identified as a unique subset of CD4+ T cells that express high levels of the IL-2R chain (CD25) along with a master transcriptional regulator, FoxP3 (Owen, Punt & Stranford, 2013). A recent study found that Treg cells with the phenotype CD4+CD25+Foxp3+ regulate inflammation and maintain immune tolerance (Jiang et al., 2021). Treg cells suppress immune responses in multiple ways by: (i) producing inhibitory cytokines to alter the metabolism of target cells, such as IL-10 and TGF-β; (ii) reducing the ability of APCs to stimulate T cells by triggering the binding of CTLA-4 on regulatory cells to B7 molecules on APCs (Shevyrev & Tereshchenko, 2019); (iii) and consuming IL-2, resulting in reduced proliferation and differentiation of other IL-2–dependent cells (Abbas, Lichtman & Pillai, 2014). The dysfunction of Treg cells is connected with the prominent breakdown of self-tolerance in RA progression (Jiang et al., 2021).

T-cell receptor (TCR) diversity goes hand in hand with effective treatment and patient prognosis in different diseases (Tong et al., 2016; Wagner et al., 1998). Complementarity-determining region 3 (CDR3), which is randomly encoded by variable (V), diversity (D), and joining (J) gene recombination, is important to the overall diversity of the TCR repertoire. (Chen et al., 2016). Total TCR diversity is primarily determined by the number of rare clonotypes in CDR3 (Yager et al., 2008). A recent study concluded that both the percentage of naive CD4+ T cells and TCR diversity decrease with aging (Britanova et al., 2014). In this study, we identified Treg cells using flow cytometry to explore the distinction between KD and non-KD RA patients. We also used a recently-developed high-throughput immune repertoire sequencing technique to explore the relationship between Treg cell immune repertoire diversity and KD in RA.

## Materials and Methods

### Patients

This study was approved by The Ethics Committee of the First Affiliated Hospital of Guangzhou University of Chinese Medicine (ZYYECK[2018]141). All donors signed an informed consent form, which notified participants of the use of their peripheral blood in this study. Peripheral blood samples from 34 RA patients and 10 healthy volunteers enrolled in this study from the Department of Rheumatology in The First Affiliated Hospital of Guangzhou University of Chinese Medicine were used for flow cytometry analysis (Table 1). The disease activity of each subject was assessed using the DAS28, developed by The European League Against Rheumatism (EULAR; Fransen & van Riel, 2009). Low disease activity was defined as 3.2>DAS28>2.6, moderate disease activity was defined as 5.1>DAS28>3.2, high disease activity was defined as DAS28>5.1, and a DAS28<2.6 indicated that the RA was in remission. TCM experts defined kidney deficiency according to the 1986 deficiency syndrome criteria of *TCM Deficiency Evidence and Geriatric Disease Research Society and Classification Standards for Diseases and Treatment Effects* established by the Chinese State Administration of TCM in 1994. This criteria for kidney deficiency syndrome includes: low back pain (excluding injury); shin pain and claudication or heel pain; limited anterior and posterior bending; hair loss or tooth tremors; dribbling urine or urinary incontinence; sexual dysfunction or infertility; fear of cold and cold extremities; and fatigue. RA patients were classified as having KD if they met five or more of these criteria. Of the 34 RA patients who participated in this study, 14 were classified as KD RA patients and 20 as non-KD RA patients. Six additional RA patients with or without KD in this study were recruited for high-throughput Treg-cell receptor sequencing in this study (Table 2).

**Table 1:** Baseline characteristics of RA patients with or without kidney-deficiency syndrome undergoing flow cytometry. The data is divided into a kidney-deficiency group and non-kidney-deficiency group and includes baseline characteristics of patients with rheumatoid arthritis who underwent flow cytometry, such as age, gender, inflammatory indicators, disease activity, *etc*.

Charactieristics	Kidney-deficiency syndrome (*n* = 14)	Non-Kidney-deficiency syndrome (*n* = 20)
Female, *n* (%)	12 (85.71)	15 (75.00)
Age (years)	55.14 (7.60)	51.20 (6.96)
ESR (mm/h)	58.91 (26.06)	45.3 (16.40)
CRP (mg/L)	32.22 (32.00)	31.33 (24.79)
DAS28-ESR	6.55 (0.75)	5.92 (0.88)*
DAS28-CRP	5.83 (0.79)	5.42 (0.90)
Rheumatoid factor (%)	10 (71.43)	17 (85.00)
ACPA (%)	12 (85.71)	20 (100.00)

**Note:**

* Significance between RA with kidney-deficiency and RA without kidney-deficiency by independent-samples t-test.

**Table 2:** Baseline characteristics of RA patients with or without kidney-deficiency syndrome recruited for TCR sequencing. The data includes baseline characteristics of patients with rheumatoid arthritis who underwent high-throughput Treg cell receptor sequencing, such as age, gender, inflammatory indicators, disease activity, *etc*.

No.	Sex	Age	Kidney-deficiency syndrome						No.	Sex	Age	Non-Kidney-deficiency syndrome					
			ESR (mm/h)	CRP (mg/L)	DAS28 (CRP)	DAS28 (ESR)	Rheumatoid factor	ACPA				ESR (mm/h)	CRP (mg/L)	DAS28 (CRP)	DAS28 (ESR)	Rheumatoid factor	ACPA
1	Female	64	55	9.32	4.49	5.50	202.00	>200	4	Female	39	30.00	6.93	4.00	4.68	<20	<0.5
2	Female	47	30	6.02	4.24	4.96	870.00	173.70	5	Female	46	42.00	2.39	4.17	5.39	456.00	133.40
3	Female	51	45	31.60	5.24	5.69	209.00	>200	6	Female	60	56.00	11.70	4.29	5.23	54.20	>200

**Note:**

ESR, Erythrocyte sedimentation rate; CRP, C reactive protein; DAS28, Disease Activity Score with 28-joint counts; N/A, not applicable; ACPA, anti-cyclic citrullinated peptide.

### Flow cytometric analysis

To analyze the percentage of CD4^+^CD25^+^ T cells, CD4^+^CD25^+^CD45RA^+^ naïve Treg cells, CD4^+^CD25^+^CD45RA^-^CD127^low^ Treg cells, and CD3^+^CD4^+^HLADR^+^ T cells in the peripheral blood drawn from RA patients, the following fluorophore-conjugated antibodies were used: anti-CD3-ECD (A07748), anti-CD4-FITC (A07750), anti-CD25-PC5 (IM2646), CD127-PE (IM1980U; all from Beckman Coulter, Inc., Brea, CA, USA), anti-human HLA-DR (HPDR-025, 4A BIOTECH, Beijing, China), and anti-human CD45RA (FNH0453-025, 4A BIOTECH, Beijing). The samples were incubated with antibodies (Table S1) at 4 °C in darkness for 15 min and then lysed with RBC lysing buffer. After two washes in PBS buffer, the cells were analyzed using a Beckman Coulter FC500 Cytometer. To ensure the rigor and authenticity of the data, the flow cytometric analysis for each sample was repeated twice. All data analyses were performed using CXP analysis software.

### PBMC isolation and FACS

Peripheral blood mononuclear cells (PBMCs) were isolated from peripheral blood samples by density gradient centrifugation using Ficoll-Paque medium (GE Healthcare, Chicago, IL, USA; Supplement Material 2). The following antibodies were used for characterization of the Treg cell pool in purified PBMCs incubated within 15 min: anti-CD4-FITC (A07750), anti-CD25-PC5 (IM2646), and CD127-PE (IM1980U; all from Beckman Coulter Inc., Brea, CA, USA). Labeled Treg cells were isolated through fluorescence-activated cell sorting (FACS) using the MoFlo Astrios Cell Sorter (Beckman Coulter).

### RNA extraction and cDNA synthesis

Total RNA was extracted from Treg cells using a TRIzol kit (Invitrogen, Waltham, MA, USA) according to the instructions from the manufacturer. Qualified RNA was reverse transcribed using SMARTScribe™ Reverse Transcriptase (Clontech, Mountain View, CA, USA), which synthesized 5′ rapid amplification of cDNA ends (RACE) for high-throughput sequencing. The resulting cDNA was purified using the MinElute PCR Purification Kit (Qiagen, Hilden, Germany) before being sequenced.

### High-throughput TCR library preparation

Two rounds of nested PCRs were used to prepare the TCR library. The first round of PCR was performed by mixing the cDNA, primers, Q5® High-Fidelity 2X Master Mix (NEB, Ipswich, MA, USA), and enzyme-free water to a final volume of 45 μl. The reaction procedures for the premixes were: one cycle at 92 °C for 2 min, 18 cycles at 94 °C for 30 s, one cycle at 60 °C for 30 s, and one cycle at 68 °C for 2 min. The products were subjected to the second round of PCR reactions with sixteen cycles under the same reaction conditions as the first round of PCR. Each round of PCR products was purified with the MinElute PCR Purification Kit (Qiagen, Hilden, Germany). The NEB Next Ultra DNA Library Prep kit was used to connect the Illumina sequencing connectors, followed by the high-throughput sequencing of PCR products using the Illumina Miseq sequencing platform (Illumina, San Diego, CA, USA).

### Data processing and analysis

The raw sequence reads obtained from high-throughput sequencing were stored in FASTQ format. Only duplicate reads with different UMIs were analyzed for downstream processing. A clonotype was defined by the CDR3 amino acid sequence and the TCRβ V, D, and J genes; clonotypes were defined according to IMGT59 and Igblast; the TCRβ V(D)J combination was defined using MIXCR.

### RT-qPCR

Peripheral blood Treg was selected from 34 subjects (including 14 RA patients with KD and 20 RA patients with non-KD). Total RNA of Tregs were extracted by TRIzol. RNA concentration was measured using a NanoDrop 2,000 spectrophotometer (Thermo Scientific, Waltham, MA, USA). Qualified RNA was reverse transcribed using PrimeScript RT Master Mix (Takara, Kusatsu, Shiga, Japan). RT-qPCR analysis was performed on an ABI Q5 instrument using the TB Green™ Premix Ex Taq™ (Takara, Kusatsu, Shiga, Japan). GAPDH as the housekeeping gene.

### Statistical analysis

Non-paired samples were compared using an independent T-test, Mann-Whitney test, and chi-square test. Statistical analyses were performed using SPSS software version 22.0 (IBM Corp., Armonk, New York, USA). Two-sided *P*-values < 0.05 were considered statistically significant.

## Results

### Flow analysis of Treg cells and their subsets

**Figure 1:**
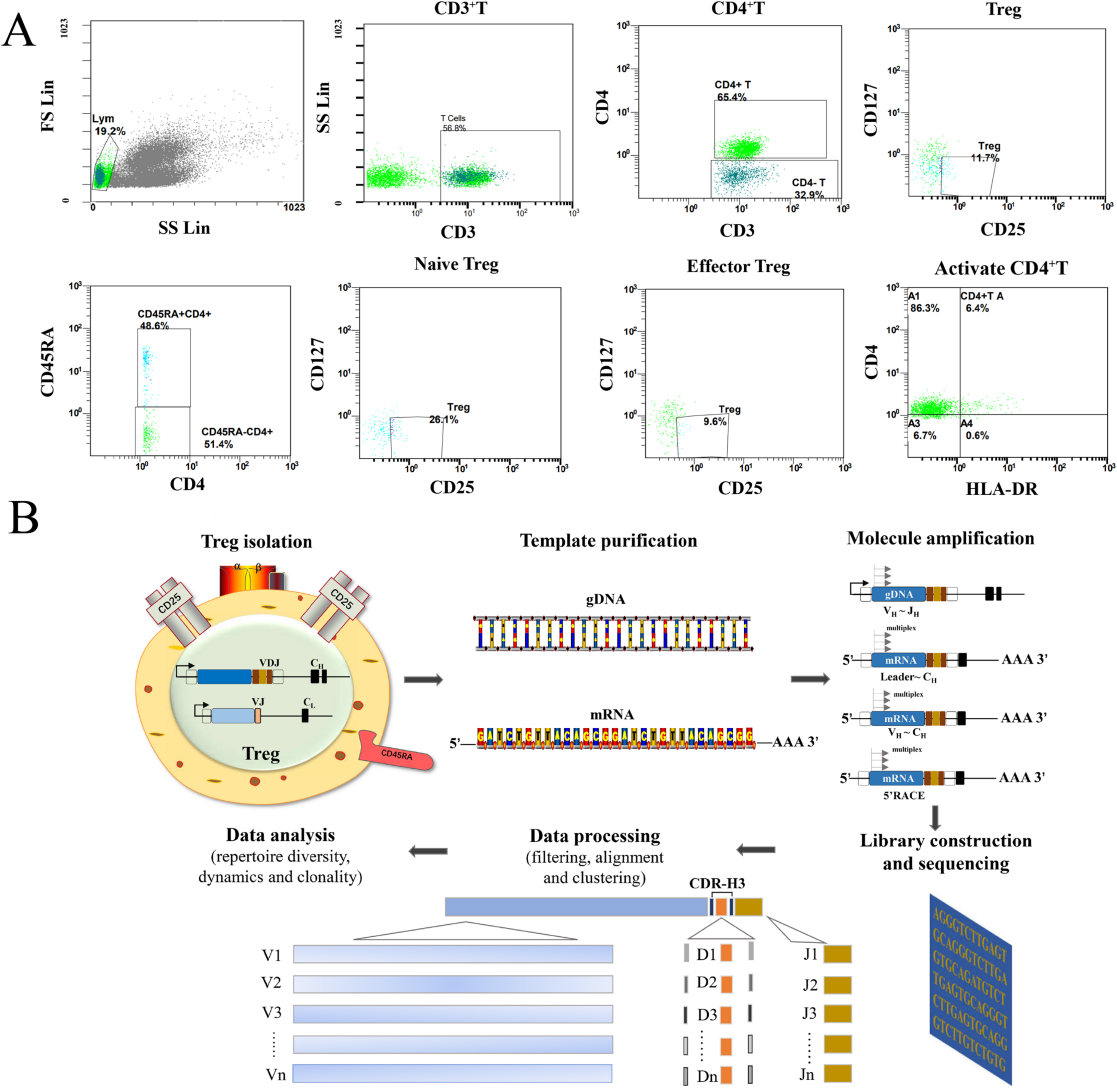
Flow analysis of Treg cells and sequencing of the high-throughput immune group library. (A) This study mainly analyzed the Treg cells (CD4+CD25+CD127−), the naïve Treg cells (CD4+CD45RA+CD25+CD127-), the effector Treg cells (CD4+CD45RA-CD25+CD127-) and the activated CD4+T cells (CD4+HLA-DR+). (B) The sequencing steps of the high-throughput immune group library include: cell separation, template extraction, amplification and construction of the database, sequencing, and a bioinformatics analysis.

**Figure 2:**
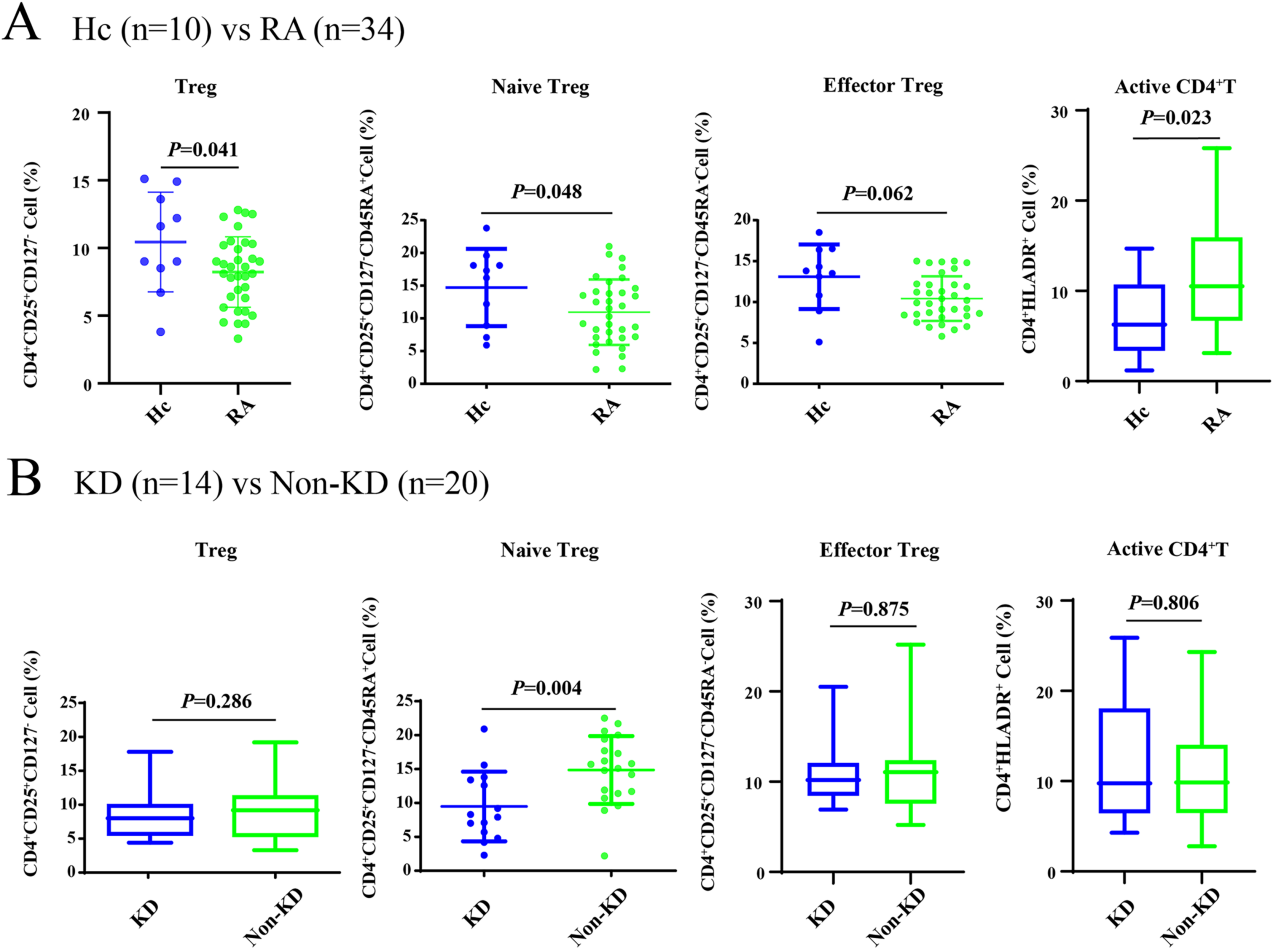
The percentage of Treg cells and their subsets in the peripheral blood of RA patients with KD, RA patients with non-KD, and healthy controls. (A) Compared with healthy controls (*n* = 10), total Treg cells, naïve Treg cells, and activated CD4+T cells were significantly lower in patients with RA (*n* = 34, *P* < 0.05). (B) There were no significant differences in total Treg cells, effector Treg cells, and activated CD4+T cells (*P* > 0.05) in RA patients diagnosed with KD (*n* = 14) compared with RA patients identified as non-KD (*n* = 20), but naïve Treg cells were significantly higher in the KD RA group (*P* < 0.05).

As shown in Fig. 1A, all Treg cells from 44 participants were labeled and sorted into four subsets using flow cytometry, including Treg cells (CD3^+^CD4^+^CD25^+^CD127^-^), naïve Treg cells (CD4^+^CD45RA^+^CD25^+^CD127^-^), effector Treg cells (CD4^+^CD45RA^-^CD25^+^CD127^-^), and activated CD4+T cells (CD4^+^HLA-DR^+^). The sequencing steps for the high-throughput immune group library are shown in Fig. 1B. The results showed that the percentage of total Treg cells (*P* = 0.041) and naïve Treg cells (*P* = 0.048) were both lower in the RA group (*n* = 34) compared to healthy participants (*n* = 10). Activated CD4+T cells (*P* = 0.023) were higher in the RA group. There were no significant differences in effector Treg cells (*P* = 0.062) in peripheral blood between the two groups of patients (Fig. 2A). All RA patients were then distributed into two groups: a KD RA group (*n* = 14), and a non-KD RA control group (*n* = 20). The percentage of naïve Treg cells was significantly different between the KD and Non-KD groups (*P* = 0.004, Fig. 2B). Similar differences were observed in total Treg cells (*P* = 0.286) and number of effector Treg cells (*P* = 0.875); differences in activated CD4+T cells (*P* = 0.806) were not significant. These findings indicate that a reduction in the percentage of naïve Treg cells is correlated with KD.

### Profiling of TRB CDR3 in RA

**Figure 3:**
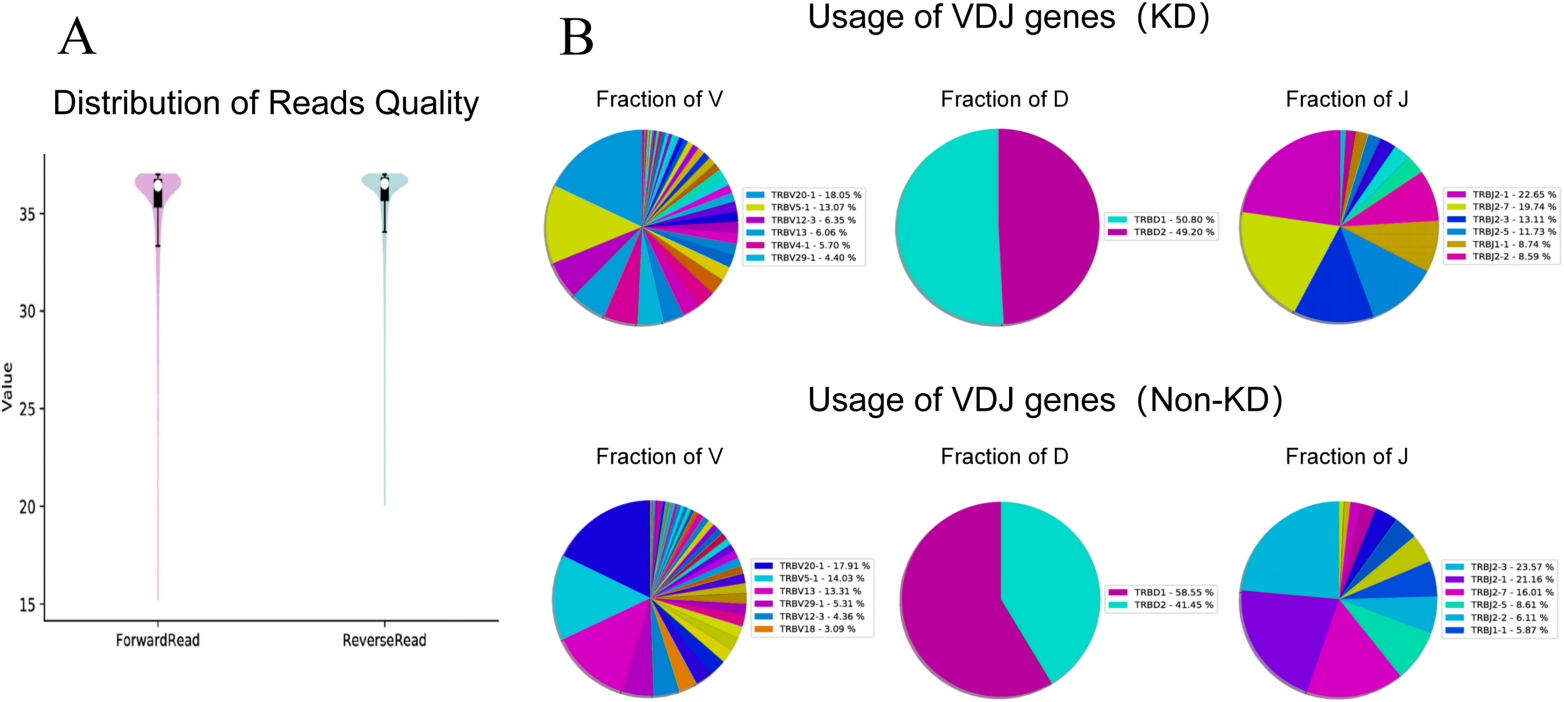
TCR repertoire sequencing analysis of peripheral Treg cells in RA patients. Sorted Treg cells from the peripheral blood of three RA patients (KD group) were analyzed using TCR sequencing. The control sample (non-KD RA group) was collected from the peripheral blood of three non-KD RA patients. (A) A total of 10,000 original reads were randomly selected to make the sequencing quality distribution map. The results showed that the median value of all data was more than 35, and the density distribution was concentrated above the median value, indicating that the data quality was high. (B) Statistical analysis of V(D)J usage frequency and pie chart showing the first six most frequently used genes, as listed in the chart legend.

**Figure 4:**
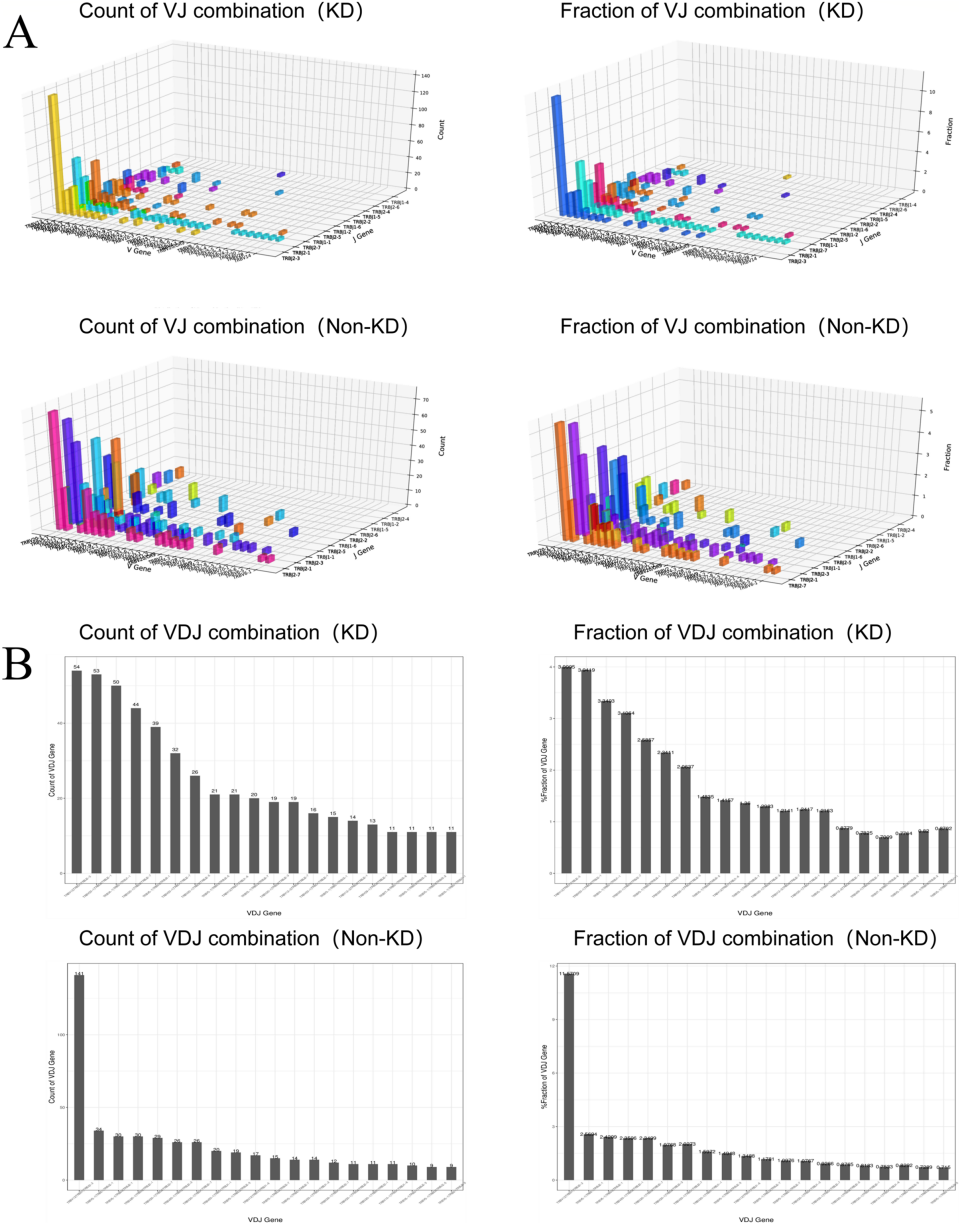
Distribution of VJ and V(D)J combinations. (A) 3D map of VJ combinations (showing the first 30 highest frequency V genes). (B) 2D V(D)J combination map (showing the 20 combinations with the highest frequencies).

We investigated the TCRβ chain repertoires of Treg cell subsets using high-throughput Treg-cell receptor sequencing to identify which the diversity of Treg-cell depends on the CDR3 and is encoded by V(D)J recombination. The sequencing results demonstrated that the quality analysis results were accurate (Fig. 3A). The use of V(D)J recombination between the KD and non-KD groups is presented as a pie chart in Fig. 3B. Both 3D (Fig. 4A) and 2D images (Fig. 4B) were used to visualize the count and fraction of T cell receptor beta (TRB) of VJ combination in the KD and non-KD groups. Overall, the non-KD group had a higher count of V(D)J recombination with a superior genotype (TRBV13|TRBD1|TRBJ2-3). The TRBV13|TRBD1|TRBJ2-3 recombination was also more highly-expressed in the non-KD group, though the difference was not significant.

### The TCRB CDR3 amino acid sequence and CDR3 length distribution assay

**Figure 5:**
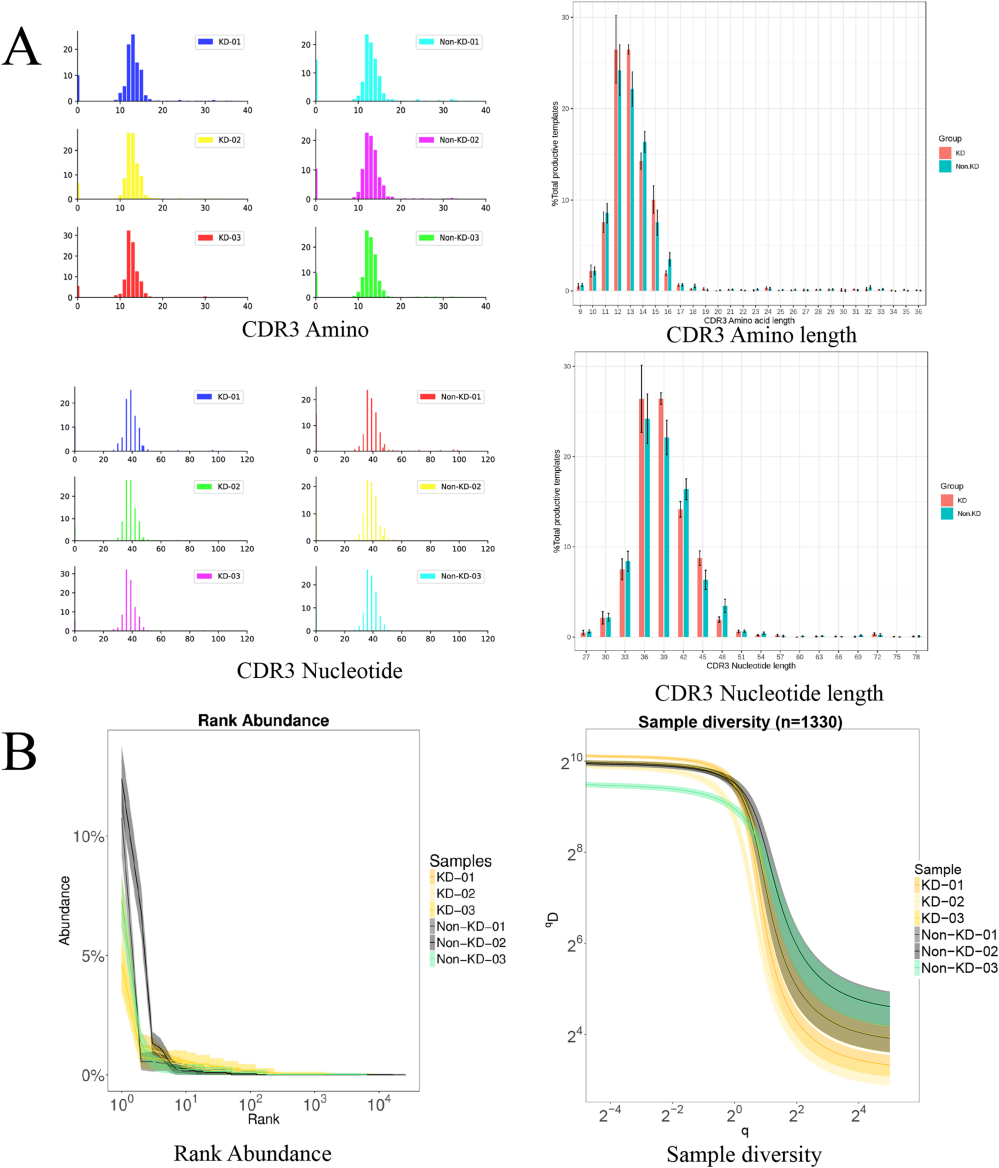
Analysis of characteristics, abundance, and diversity of the TCR CDR3 region of Treg cells. (A) Length distribution of CDR3 amino acids and nucleic acids. (B) The rank abundance curve was used to analyze diversity. The width of the curve indicates abundance: the wider the curve (as measured by the x axis), the higher the abundance of the CDR3 region. The richness of the non-KD group was higher than the KD group. Hill numbers are a function of continuous variable Q, which can directly reflect diversity using: the richness index, the Shannon index, and the Simpson index. The abscissa represents different Q values, and the ordinate indicates the diversity of samples under that Q value. When Q approaches infinity, the Hill index can reflect the largest component frequency in the sample. The clones of Treg cells in the KD group were less diverse than those in the non-KD group.oup were less diverse than those of Non-KD group.

The TCRB CDR3 amino acid sequence and CDR3 length distribution assay were used to verify the TCR diversity of Treg cells. A bar chart was used to visualize differences in TCR CDR3 lengths (Fig. 5A). The results showed that there was no significant difference in the number of total TCRβ CDR3 amino acid sequences between the KD and non-KD groups, though the amino acid sequences were shorter in the KD group. There was also no significant difference in TCRβ CDR3 Nucleotide amino acid sequences between the two groups, but these sequences were larger in the non-KD group.

Because the smooth level reflected the samples evenness of CDR3 length in rank abundance, the cliffy curve revealed that the non-KD groups had more discrete variation in CDR3 length than the KD group. In addition, Hill numbers were used to measure TCR diversity using the richness index, Shannon index, and Simpson index. As shown in Fig. 5B, there were significant differences between the KD group and the non-KD group, indicating TCR diversity is closely tied with KD.

### The usage patterns of Vb, Db, and Jb gene segments

**Figure 6:**
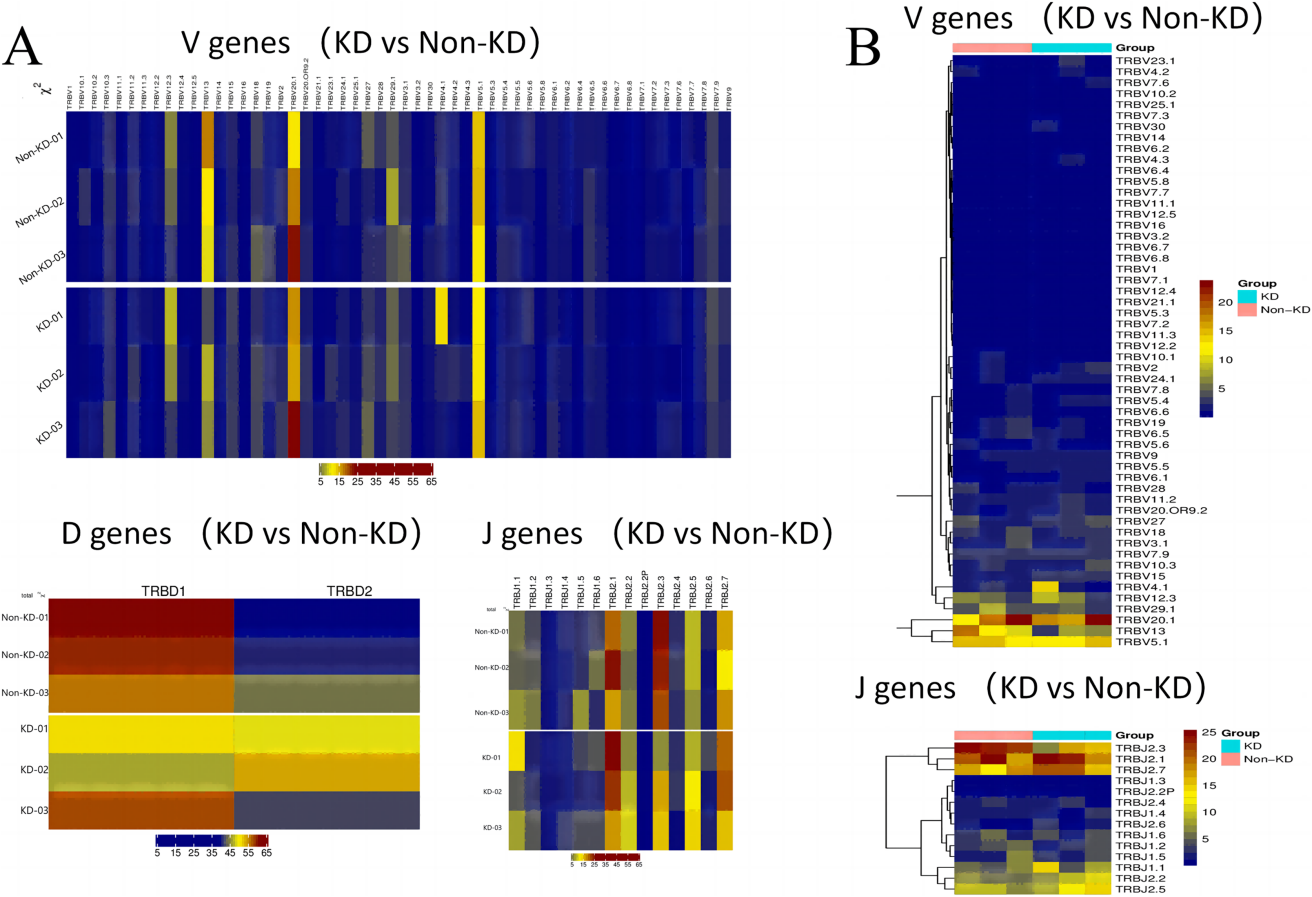
Differences in V(D)J gene usage (*via* heatmap). (A) A chi-square test was used to analyze the differences in V(D)J gene usage. (B) A rank sum test (Wilcox test) was used to analyze the differences of V and J genes between the two groups.

**Figure 7:**
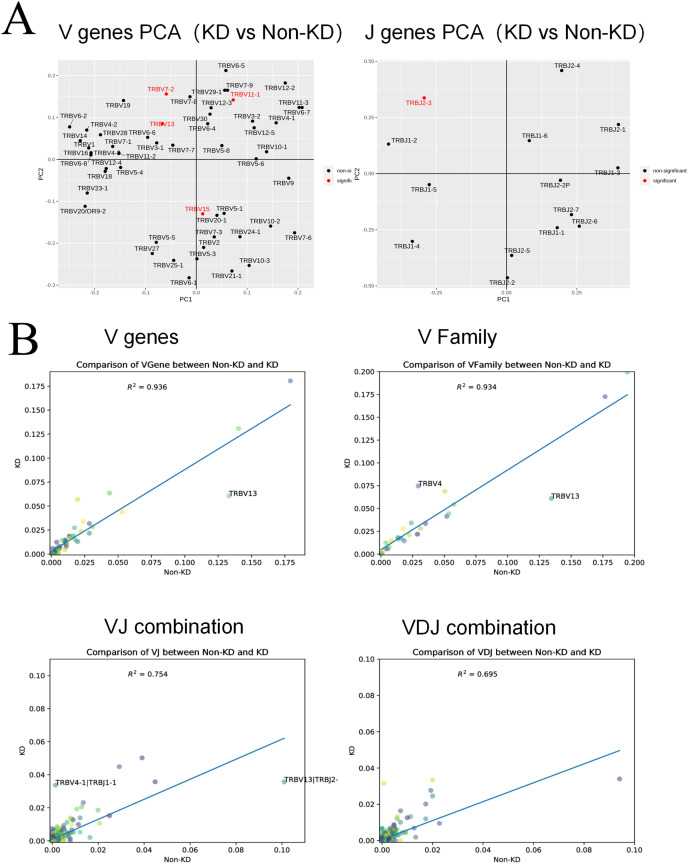
Collinear analysis of TCR CDR3 V(D)J differential genes and their combinations in Treg cells. (A) A principal component analysis (PCA) was used for a cluster analysis of V and J genes between the two groups. (B) The collinearity graph of the V gene, V family gene, VJ gene, and V(D)J gene between the KD and non-KD group.

A heatmap was used to evaluate the TCR usage patterns of Vb, Db, and Jb gene segments. A scatterplot using the PCA method was used to evaluate the expression levels of each gene in the two groups. As shown in Figs. 6A and 6B, there were similar patterns observed in both the KD and non-KD groups for the Vb, Db, and Jb genes, but several Vb and Jb gene segments differed significantly between the two groups. Of the Vb gene segments, TRBV7-2 and TRBV13 were significantly more frequent in the non-KD group (*P* < 0.05), which may indicate these two TRVBs may decrease with age and play a prominent role in growth and aging. TRBV11-1 gene segments were significantly less frequent in the non-KD group (*P* < 0.05), and the Jb gene segment, TRBJ2-3, was significantly less frequent in the KD group (Fig. 7A). There were no significant differences in Db gene segments between the two groups (all *P*-values were > 0.05).

V(D)J collinearity was analyzed (line chart, Fig. 7B) and the sample standard deviation was found to be high. An *R*^*2*^ value close to one indicates that the samples are similar. The *R*^*2*^ values of both the V gene (*R*^*2*^= 0.936) and V family (*R*^*2*^= 0.934) were higher than 0.80, indicating no significant differences. Conversely, the *R*^*2*^ values of the VJ gene (*R*^*2*^= 0.754) and VJ family (*R*^*2*^= 0.695) were significant.

## Discussion

RA is an age-related, systemic inflammatory autoimmune disease characterized by defective adaptive immunity and a chronic state of inflammation (Santoro, Bientinesi & Monti, 2021; Sparks, 2019). T-cell dysfunction, leading to an abnormal proliferation of T cells, is considered one of the main mechanisms of RA pathogenesis (Malemud, 2018). One previous study found that the aging process is closely tied to immunosenescence, including the deterioration of the regenerative capacity of T cells (Bauer, 2020). As people age, the defects of naïve T cells and alterations to TCR repertoire diversity cause immune aging, leading to chronic tissue inflammation (Appay & Sauce, 2014; Weyand, Yang & Goronzy, 2014). Thus, RA is directly tied to premature immunosenescence as it combines immune dysfunction with chronic inflammation (Pawelec, 2012; Santoro, Bientinesi & Monti, 2021).

In TCM theory, the aging of the body leads to the continuous dissipation of both essence and qi in the kidneys, with increased dissipation in the pathologic state (Dong et al., 2013). *Basic theories of traditional Chinese medicine* states (Wang & Zhu, 2011): “Once people reach middle age, kidney energy starts to decline.” There is a strong link between aging and KD. A previous study found that KD and aging are similar in the nervous-endocrine-immune networks and conclude that: “the essence of aging is physiological KD” (Shen, Chen & Huang, 2006). RA, diagnosed as “BiZhen,” is frequently attributed to KD. Our preliminary study found that KD RA patients had higher disease activity, including more defective immunity and inflammation, than non-KD individuals (Mo et al., 2016). However, KD’s mechanism for causing defective adaptive immunity, especially T cells, was still unknown.

Treg cells play a pivotal role in the regulation of adaptive immunity and prevention of autoimmune diseases (Wing & Sakaguchi, 2011). Treg cells utilize a distinct TCR repertoire to regulate their function; defective Treg function is associated with the development of RA (Esensten, Wofsy & Bluestone, 2009; Liu et al., 2019). In human studies, reduced Treg-cell populations indicate defective adaptive immunity, which leads to the exacerbated form of RA with increased disease activity and an excessive inflammatory response (Lawson et al., 2006; Okeke & Uzonna, 2019). Our study used flow cytometry to analyze the amount of Treg cells and their subpopulations in the peripheral blood of KD RA patients and non-KD RA patients.

With the exception of effector Treg cells (CD4+CD45RA-CD25+CD127-), total Treg cells, naïve Treg cells, and activated CD4+T cells all differed significantly between RA patients and healthy controls, which expands the findings of previous studies (Kawashiri et al., 2011; Walter et al., 2013; Walter et al., 2016). This study found that the number of effector Treg cells in RA patients was similar to those found in healthy participants. While there were no significant differences in the total amount of effector Treg cells between the two groups, effector Treg cells in RA patients showed increased expression of pro-inflammatory cytokines and maintained their Treg phenotype and function (Walter et al., 2013). CD25^int^ effector Treg cells (CD4+CD45RA-CD25^int^CD127-) contained increased percentages of IL-17+ and TNF+ cells, suggesting a potentially higher activation status (Walter et al., 2016). This conclusion also helps explain the higher number of activated T cells observed in this study. However, it is still unclear whether effector Treg cells in the peripheral blood of RA patients have decreased suppressive capacity compared to those in healthy controls.

Our study also measured Treg-cell subsets and found a sharp decline in naïve Treg cells in KD RA patients compared to non-KD RA patients. However, there were no significant differences in the total number of Treg cells and other Treg-cell subsets observed between the two groups. It is well known that the number of circulating Treg cells, Treg-cell subset distribution, and Treg cell functionality affect immune homeostasis and function (Bektas et al., 2017). A recent study found that impaired Treg cell homeostasis and function increase the risk of immune-mediated disorders (Haribhai et al., 2011).

Previous studies have found that premature naïve T-cell aging, accompanied by defective DNA repair responses, results in the compromised function of Treg cells in RA patients (Fessler et al., 2017; Goronzy et al., 2015). Furthermore, the function of Treg cells declines significantly with age. Older individuals with an abnormal number of Treg cells are unable to regulate the production of anti-inflammatory cytokines, such as IL-10, resulting in a chronic, low-grade proinflammatory state. The results of this study demonstrated that KD, which is directly tied to immunosenescence, was positively correlated with a decline in the number of naïve Treg cells as well as the loss of Treg function. There were no significant differences in the total number of Treg cells, effector Treg cells, or activated T cells between KD RA and non-KD RA patients, though the Treg cells and effector Treg cells may be functionally different between the two groups. Treg cells have reduced functional activity *in vivo*, which may lead to the proliferation of various T-cell clones, including self-reactive clones, resulting in reduced TCR diversity and increased risk of autoimmunity (Shevyrev et al., 2021). This study investigated the TCR receptors of Treg cells using high-throughput sequencing and found reduced TCR diversity in Treg cells, which is in agreement with existing findings.

Treg cells require high-affinity TCR ligation for antigen recognition and the antigen-specific immune response (Levine et al., 2014). This study used immune repertoire sequencing to explore the repertoires of Treg cells in KD and non-KD patients. A decline in TCRβ CDR3 diversity was observed in KD patients compared to non-KD patients, which means shorter CDR3 length and lower numbers of added nucleotides. This result requires further investigation because it was only marginally significant. The TCR repertoire constriction that occurs in KD patients, including decreased CDR3 length and increased clonotypes, is likely part of the aging process. Researchers have discovered that as people get older, there is a decrease in CDR3 length, NDN insert, and number of non-template added N nucleotides within TCR beta CDR3 (Egorov et al., 2018). This phenomenon may reflect exhaustion of naïve T cells with higher affinity to self MHC, making it difficult to maintain a diverse repertoire (Chung et al., 2009; Myers, Zikherman & Roose, 2017). This change indicates T-cell dysfunction and leads to immunosenescence, which may reduce infection resistance in older adults (Minato, Hattori & Hamazaki, 2020).

Kidney deficiency plays a key role in shaping T-cell function with age, affecting levels of inflammation and immunosenescence. Hill numbers were used to measure Treg cell diversity using the richness index, Shannon index, and Simpson index (Chiffelle et al., 2020; Ma & Li, 2018). The results showed that the abundance and diversity of Treg cells were lower in the KD group compared to the non-KD group. Key findings from this study are that KD leads to a decrease in the number of native Treg cells and a decrease in TCR diversity.

V(D)J combinations in both KD and non-KD patients were analyzed using a chi-square test and other statistical analyses, and several specific VJ combinations were observed. Some VJ combinations, such as TRBV7-2, TRBV11-1, TRBV13, TRBV15, TRBJ2-3, appeared with high frequency. Existing research indicates that TRBV7-2 and TRBV13 are related to immunosenescence (Lian et al., 2019; Liu et al., 2012; Nakashima et al., 1996). Previous studies of TCR β-chain repertoire found that TRBV7-2 expression was higher in people aged 6–20 years, indicating that there is a possible correlation between TRBV7-2 and youth. Disability also increases with age, so non-KD RA patients may have lower disability and disease activity levels (Lian et al., 2019). TRBV13 seems to initiate the development of autoimmunity. One animal study indicated that anti–Vβ13+TCR antibodies may help to prevent autoimmune diabetes in rats, but the mechanism behind this possibility was not reported (Liu et al., 2012). An increase in the level of T cells expressing TRBV13 is also associated with the progression of autoimmune thyroiditis (Nakashima et al., 1996).

RT-qPCR showed that FOXP3 expression in peripheral blood Treg is lower in the KD group than in the non-KD group, confirming the accuracy of our data (Fig. S1). CD25, CD127, and FOXP3 are the three canonical Tregs markers (Reinhardt et al., 2022). The expression of FOXP3 in human naïve Treg was lower than in other Treg subgroups (Miyara et al., 2009). Therefore, the decrease in FOXP3 expression in the KD group represents an increase in the proportion of naïve Treg.

This study has a number of limitations that should be considered in future experiments. The heterogeneity of our sample resulted in some conflicting results; future research should attempt to duplicate detection or increase the sample size. The etiopathogenesis of KD might also be more than just KD. Age-associated KD is an indicator of RA, but some RA cases cannot be explained by KD; some RA cases can be explained by the TCM wind-cold-dampness theory. Therefore, a better method of stratifying RA cases is needed (Chen et al., 2020). This study also has a number of strengths. To the best of our knowledge, this is the first study to identify how KD correlates with immunosenescence in RA from an immunological perspective. Flow cytometry and high-throughput sequencing technology have also not been broadly used to explain the relationship between KD and RA (Zhang & Zhao, 2019). This study provides quantitative measurements and new evaluation methods for the prevention of RA. Based on the results of this study Treg cell-based therapies, which target defective Treg cell functions, may be a promising therapeutic approach to RA (Schlöder et al., 2022; Yan et al., 2022). Previous studies have shown that Treg cell-based therapy can delay the onset of autoimmune inflammation (Dijke et al., 2016). Treg cell-based therapies may be able to ameliorate impaired Treg-cell function and increase Treg cells in RA patients. When exogenous Treg cells were infused in CIA mice, it was possible to significantly improve the severity of arthritis by increasing the proportion of Treg cells in the spleen and peripheral blood (Li et al., 2020). These findings may provide new knowledge for the development of clinical trials of Treg cell-based therapies in arthritis.

## Conclusions

The results of this study indicate that KD causes a decrease in naïve Treg cells and TCR diversity in RA, reflecting declines in the adaptive immune response. Age-associated immunosenescence and immune repertoires were strongly correlated with KD. This study highlights KD’s connection to RA, and provides quantitative measurements and modern evaluation methods for preventing and combating disease in TCM.

## Supplemental Information

10.7717/peerj.14837/supp-1 Supplemental Information 1 Flow cytometry raw data (Table 2A.Hc *vs* RA). Each data point indicates the flow cytometry of each subject.

10.7717/peerj.14837/supp-2 Supplemental Information 2 Flow cytometry raw data (Table 2B.KD *vs* Non-KD). Each data point indicates the flow cytometry of each subject.

10.7717/peerj.14837/supp-3 Supplemental Information 3 Baseline characteristics (Fig. 1). Each data point indicates the baseline characteristics of each subject, such as age, sex, CRP, ESR, *etc*.

10.7717/peerj.14837/supp-4 Supplemental Information 4 Procedure for isolation of PBMCs. The text describes each step of the peripheral blood isolation.

10.7717/peerj.14837/supp-5 Supplemental Information 5 Antibodies used in flow cytometry. The table includes specific information on the antibodies used in flow cytometry.

10.7717/peerj.14837/supp-6 Supplemental Information 6 AllSample V Fraction and count. Fraction and count of the V gene for each sample are included in the dataset.

10.7717/peerj.14837/supp-7 Supplemental Information 7 AllSample D Fraction and count. Fraction and count of the D gene for each sample are included in the dataset.

10.7717/peerj.14837/supp-8 Supplemental Information 8 AllSample J Fraction and count. Fraction and count of the J gene for each sample are included in the dataset.

10.7717/peerj.14837/supp-9 Supplemental Information 9 AllSample VDJ Combination fraction and count. Fractions and frequencies of the VDJ combination for each sample are included in the dataset.

10.7717/peerj.14837/supp-10 Supplemental Information 10 Relative mRNA expression of CD25 (A), CD127 (B) and FOXP3 (C) of RA patients with KD or non-KD. ns means no significant between two groups.

10.7717/peerj.14837/supp-11 Supplemental Information 11 RT-qPCR raw data(CD25). Relative mRNA expression of CD25 of RA patients with KD or non-KD.

10.7717/peerj.14837/supp-12 Supplemental Information 12 RT-qPCR raw data (FOXP3). Relative mRNA expression of FOXP3 of RA patients with KD or non-KD.

10.7717/peerj.14837/supp-13 Supplemental Information 13 RT-qPCR raw data (CD127).xls. Relative mRNA expression of CD127 of RA patients with KD or non-KD.

## Additional Information and Declarations

### Data Availability

The following information was supplied regarding data availability: Raw sequencing data have been deposited in the NCBI Sequence Read Archive (SRA) database under the accession number PRJNA866871.
