# Supplementary material for: High-throughput Treg cell receptor sequencing reveals differential immune repertoires in rheumatoid arthritis with kidney deficiency
Source: PeerJ. 2023 Feb 2;11:e14837. doi: 10.7717/peerj.14837 (PMC9899432; doi:10.7717/peerj.14837)
Supplement: Supplemental Information 4 — The text describes each step of the peripheral blood isolation. [file peerj-11-14837-s004.docx]

Procedure for isolation of PBMCs

1. Mix anticoagulant-treated peripheral blood with PBS in equal volume;

2. Add Ficoll-Paque to the centrifuge tube;

3. Carefully layer the diluted blood sample on Ficoll-Paque (Note: When layering the sample do not mix Ficoll-Paque and the diluted blood sample) ;

4. Centrifuge at 2200 rpm for 30 min at 18°C to 20°C;

5. Draw off the upper layer containing plasma and platelets using a sterile pipette, leaving the layer of PBMCs undisturbed at the interface;

6. Transfer the layer of mononuclear cells to a sterile centrifuge tube using a sterile pipette;

7. Estimate the volume of the transferred mononuclear cells. Add at least 3 volumes of PBS to resuspend the PBMCs in the centrifuge tube;

8. Centrifuge at 2200 rpm for 15 min at 18°C to 20°C;

9. Remove the supernatant;

10. Repeat actions 7-8 for 2 times;

11. Purified PBMCs continue to be used for fluorescence-activated cell sorting (FACS) of Treg cells.
