# Supplementary material for: High-throughput Treg cell receptor sequencing reveals differential immune repertoires in rheumatoid arthritis with kidney deficiency
Source: PeerJ. 2023 Feb 2;11:e14837. doi: 10.7717/peerj.14837 (PMC9899432; doi:10.7717/peerj.14837)
Supplement: Supplemental Information 5 — The table includes specific information on the antibodies used in flow cytometry. [file peerj-11-14837-s005.docx]

|  |  |  |  |
| --- | --- | --- | --- |
| **Supplement Table1. Antibodies used in flow cytometry** | | | |
| Antibody | Company | Product No. | Dosage |
| anti-CD3-ECD | Beckman Coulter, Inc. USA | A07748 | 10μL/100μL test sample |
| anti-CD4-FITC | Beckman Coulter, Inc. USA | A07750 | 10μL/100μL test sample |
| anti-CD25-PC5 | Beckman Coulter, Inc. USA | IM2646 | 10μL/100μL test sample |
| anti-CD127-PE | Beckman Coulter, Inc. USA | IM1980U | 10μL/100μL test sample |
| anti-human HLA-DR | 4A BIOTECH, Beijing | FHPDR | 10μL/100μL test sample |
| anti-human CD45RA | 4A BIOTECH, Beijing | FNH0453 | 10μL/100μL test sample |
|  |  |  |  |
