# Supplementary material for: High-throughput Treg cell receptor sequencing reveals differential immune repertoires in rheumatoid arthritis with kidney deficiency
Source: PeerJ. 2023 Feb 2;11:e14837. doi: 10.7717/peerj.14837 (PMC9899432; doi:10.7717/peerj.14837)
Supplement: Supplemental Information 6 — Fraction and count of the V gene for each sample are included in the dataset. [file peerj-11-14837-s006.docx]

**Supplement Table2. AllSample V Fraction and count**

| Gene | | KD-01_Fraction | KD-02_Fraction | KD-03_Fraction | Non-KD-01_Fraction | Non-KD-02_Fraction | Non-KD-03_Fraction | KD-01_count | KD-02_count | KD-03_count | Non-KD-01_count | Non-KD-02_count | Non-KD-03_count |
| --- | --- | --- | --- | --- | --- | --- | --- | --- | --- | --- | --- | --- | --- |
| TRBV1 | | 0 | 0.1222 | 0 | 0.0931 | 0 | 0 | 0 | 2 | 0 | 1 | 0 | 0 |
| TRBV10-1 | | 0.4011 | 0.2445 | 0.8239 | 0.1862 | 2.2599 | 0.3257 | 6 | 4 | 8 | 2 | 32 | 4 |
| TRBV10-2 | | 0.7353 | 0.1222 | 0.7209 | 0.4655 | 0.1412 | 0.2443 | 11 | 2 | 7 | 5 | 2 | 3 |
| TRBV10-3 | | 2.139 | 1.8949 | 3.6045 | 2.1415 | 2.5424 | 2.0358 | 32 | 31 | 35 | 23 | 36 | 25 |
| TRBV11-1 | | 0 | 0 | 0 | 0.1862 | 0.4237 | 0.1629 | 0 | 0 | 0 | 2 | 6 | 2 |
| TRBV11-2 | | 1.2701 | 2.5672 | 1.5448 | 1.5829 | 2.048 | 1.3029 | 19 | 42 | 15 | 17 | 29 | 16 |
| TRBV11-3 | | 0.6684 | 0 | 0.103 | 0.2793 | 0.2825 | 0.2443 | 10 | 0 | 1 | 3 | 4 | 3 |
| TRBV12-2 | | 0.4011 | 0 | 0 | 0.3724 | 0.4944 | 0.1629 | 6 | 0 | 0 | 4 | 7 | 2 |
| TRBV12-3 | | 8.623 | 6.7237 | 2.5747 | 6.1453 | 4.8023 | 2.5244 | 129 | 110 | 25 | 66 | 68 | 31 |
| TRBV12-4 | | 0.0668 | 0.489 | 0.206 | 0.2793 | 0.2825 | 0.0814 | 1 | 8 | 2 | 3 | 4 | 1 |
| TRBV12-5 | | 0.1337 | 0 | 0 | 0.0931 | 0 | 0 | 2 | 0 | 0 | 1 | 0 | 0 |
| TRBV13 | | 3.4759 | 8.2518 | 6.4882 | 17.9702 | 12.7825 | 10.4235 | 52 | 135 | 63 | 193 | 181 | 128 |
| TRBV14 | | 0.4011 | 0.7335 | 0.4119 | 0.7449 | 0.2825 | 0.6515 | 6 | 12 | 4 | 8 | 4 | 8 |
| TRBV15 | | 3.008 | 2.5672 | 2.7806 | 1.676 | 1.4831 | 2.1173 | 45 | 42 | 27 | 18 | 21 | 26 |
| TRBV16 | | 0 | 0.0611 | 0 | 0 | 0 | 0 | 0 | 1 | 0 | 0 | 0 | 0 |
| TRBV18 | | 2.0722 | 3.3619 | 3.1926 | 2.9795 | 2.048 | 4.4788 | 31 | 55 | 31 | 32 | 29 | 55 |
| TRBV19 | | 1.1364 | 2.0171 | 0.8239 | 1.1173 | 1.2712 | 3.013 | 17 | 33 | 8 | 12 | 18 | 37 |
| TRBV2 | | 0.9358 | 1.7726 | 2.6777 | 0.838 | 2.048 | 0.8958 | 14 | 29 | 26 | 9 | 29 | 11 |
| TRBV20-1 | | 17.1123 | 16.0147 | 23.7899 | 12.8492 | 17.9379 | 23.2085 | 256 | 262 | 231 | 138 | 254 | 285 |
| TRBV20/OR9-2 | 1.2032 | 2.445 | 1.8538 | 1.3035 | 1.1299 | 1.6287 | 18 | 40 | 18 | 14 | 16 | 20 |  |
| TRBV21-1 | 0.0668 | 0.0611 | 0.6179 | 0.1862 | 0.2119 | 0 | 1 | 1 | 6 | 2 | 3 | 0 |  |
| TRBV23-1 | 0.2005 | 2.3839 | 0.9269 | 0.6518 | 0.2119 | 0.2443 | 3 | 39 | 9 | 7 | 3 | 3 |  |
| TRBV24-1 | 1.2701 | 1.2836 | 1.8538 | 0.5587 | 1.4124 | 0.3257 | 19 | 21 | 18 | 6 | 20 | 4 |  |
| TRBV25-1 | 0.3342 | 0.5501 | 0.8239 | 0.6518 | 0.3531 | 0.1629 | 5 | 9 | 8 | 7 | 5 | 2 |  |
| TRBV27 | 2.4733 | 3.4841 | 4.7374 | 4.2831 | 1.4124 | 1.9544 | 37 | 57 | 46 | 46 | 20 | 24 |  |
| TRBV28 | 1.5374 | 2.2005 | 1.1329 | 2.4209 | 0.565 | 2.0358 | 23 | 36 | 11 | 26 | 8 | 25 |  |
| TRBV29-1 | 4.6123 | 5.1956 | 2.9866 | 4.0037 | 7.6977 | 3.8274 | 69 | 85 | 29 | 43 | 109 | 47 |  |
| TRBV3-1 | 1.8048 | 2.2616 | 2.4717 | 1.9553 | 2.1186 | 4.5603 | 27 | 37 | 24 | 21 | 30 | 56 |  |
| TRBV3-2 | 0 | 0 | 0 | 0 | 0.0706 | 0 | 0 | 0 | 0 | 0 | 1 | 0 |  |
| TRBV30 | 1.2032 | 0.6112 | 0.4119 | 0.3724 | 0.2825 | 1.1401 | 18 | 10 | 4 | 4 | 4 | 14 |  |
| TRBV4-1 | 13.3021 | 1.7115 | 0.9269 | 1.9553 | 2.4011 | 1.4658 | 199 | 28 | 9 | 21 | 34 | 18 |  |
| TRBV4-2 | 0.6016 | 2.0782 | 0.5149 | 1.3966 | 0.9181 | 0.7329 | 9 | 34 | 5 | 15 | 13 | 9 |  |
| TRBV4-3 | 0.0668 | 1.4059 | 0 | 0.0931 | 0 | 0 | 1 | 23 | 0 | 1 | 0 | 0 |  |
| TRBV5-1 | 12.2995 | 12.9584 | 15.036 | 14.6182 | 15.1836 | 12.785 | 184 | 212 | 146 | 157 | 215 | 157 |  |
| TRBV5-3 | 0 | 0 | 0.4119 | 0.2793 | 0 | 0.0814 | 0 | 0 | 4 | 3 | 0 | 1 |  |
| TRBV5-4 | 0.8021 | 1.2836 | 1.2358 | 0.9311 | 0.7768 | 1.873 | 12 | 21 | 12 | 10 | 11 | 23 |  |
| TRBV5-5 | 1.2032 | 1.3447 | 1.8538 | 1.0242 | 0.565 | 1.5472 | 18 | 22 | 18 | 11 | 8 | 19 |  |
| TRBV5-6 | 2.139 | 0.9169 | 1.1329 | 1.7691 | 0.8475 | 0.57 | 32 | 15 | 11 | 19 | 12 | 7 |  |
| TRBV5-8 | 0.2674 | 0 | 0.103 | 0.5587 | 0.0706 | 0.0814 | 4 | 0 | 1 | 6 | 1 | 1 |  |
| TRBV6-1 | 1.0027 | 1.2836 | 1.8538 | 1.0242 | 1.0593 | 0.8958 | 15 | 21 | 18 | 11 | 15 | 11 |  |
| TRBV6-2 | 0.3342 | 0.9169 | 0.4119 | 0.5587 | 0.4944 | 0.7329 | 5 | 15 | 4 | 6 | 7 | 9 |  |
| TRBV6-4 | 0.0668 | 0.0611 | 0 | 0.9311 | 0.565 | 0 | 1 | 1 | 0 | 10 | 8 | 0 |  |
| TRBV6-5 | 1.6711 | 1.2836 | 0.8239 | 1.0242 | 2.2599 | 2.443 | 25 | 21 | 8 | 11 | 32 | 30 |  |
| TRBV6-6 | 0.6016 | 0.9169 | 0.9269 | 0.9311 | 1.0593 | 1.3844 | 9 | 15 | 9 | 10 | 15 | 17 |  |
| TRBV6-7 | 0.0668 | 0 | 0 | 0 | 0.0706 | 0 | 1 | 0 | 0 | 0 | 1 | 0 |  |
| TRBV6-8 | 0 | 0.1834 | 0 | 0 | 0 | 0 | 0 | 3 | 0 | 0 | 0 | 0 |  |
| TRBV7-1 | 0 | 0.1222 | 0 | 0.1862 | 0 | 0 | 0 | 2 | 0 | 2 | 0 | 0 |  |
| TRBV7-2 | 0.1337 | 0.1834 | 0.103 | 0.4655 | 0.3531 | 0.7329 | 2 | 3 | 1 | 5 | 5 | 9 |  |
| TRBV7-3 | 0.4011 | 0.1834 | 0.9269 | 0.7449 | 0.2825 | 0.6515 | 6 | 3 | 9 | 8 | 4 | 8 |  |
| TRBV7-6 | 0.8021 | 0.0611 | 1.2358 | 0.1862 | 0.7062 | 0.4886 | 12 | 1 | 12 | 2 | 10 | 6 |  |
| TRBV7-7 | 0.0668 | 0.0611 | 0 | 0.7449 | 0 | 0 | 1 | 1 | 0 | 8 | 0 | 0 |  |
| TRBV7-8 | 1.1364 | 1.0391 | 0.6179 | 0.3724 | 1.0593 | 1.7915 | 17 | 17 | 6 | 4 | 15 | 22 |  |
| TRBV7-9 | 3.5428 | 3.1785 | 2.5747 | 2.8864 | 3.0367 | 2.7687 | 53 | 52 | 25 | 31 | 43 | 34 |  |
| TRBV9 | 2.2059 | 1.4059 | 1.7508 | 0.9311 | 1.6949 | 1.2215 | 33 | 23 | 17 | 10 | 24 | 15 |  |
