# Supplementary material for: High-throughput Treg cell receptor sequencing reveals differential immune repertoires in rheumatoid arthritis with kidney deficiency
Source: PeerJ. 2023 Feb 2;11:e14837. doi: 10.7717/peerj.14837 (PMC9899432; doi:10.7717/peerj.14837)
Supplement: Supplemental Information 7 — Fraction and count of the D gene for each sample are included in the dataset. [file peerj-11-14837-s007.docx]

| Gene | KD-01_Fraction | KD-02_Fraction | KD-03_Fraction | Non-KD-01_Fraction | Non-KD-02_Fraction | Non-KD-03_Fraction | KD-01_count | KD-02_count | KD-03_count | Non-KD-01_count | Non-KD-02_count | Non-KD-03_count |
| --- | --- | --- | --- | --- | --- | --- | --- | --- | --- | --- | --- | --- |
| TRBD1 | 51.2701 | 46.2714 | 57.7755 | 60.6145 | 59.2514 | 55.8632 | 767 | 757 | 561 | 651 | 839 | 686 |
| TRBD2 | 48.7299 | 53.7286 | 42.2245 | 39.3855 | 40.7486 | 44.1368 | 729 | 879 | 410 | 423 | 577 | 542 |

**Supplement Table3. AllSample D Fraction and count**
