# Supplementary material for: High-throughput Treg cell receptor sequencing reveals differential immune repertoires in rheumatoid arthritis with kidney deficiency
Source: PeerJ. 2023 Feb 2;11:e14837. doi: 10.7717/peerj.14837 (PMC9899432; doi:10.7717/peerj.14837)
Supplement: Supplemental Information 8 — Fraction and count of the J gene for each sample are included in the dataset. [file peerj-11-14837-s008.docx]

**Supplement Table 4. AllSample J Fraction and count**

| Gene | KD-01_Fraction | KD-02_Fraction | KD-03_Fraction | Non-KD-01_Fraction | Non-KD-02_Fraction | Non-KD-03_Fraction | KD-01_count | KD-02_count | KD-03_count | Non-KD-01_count | Non-KD-02_count | Non-KD-03_count |
| --- | --- | --- | --- | --- | --- | --- | --- | --- | --- | --- | --- | --- |
| TRBJ1-1 | 14.0374 | 4.401 | 8.033 | 5.6797 | 4.8023 | 7.329 | 210 | 72 | 78 | 61 | 68 | 90 |
| TRBJ1-2 | 1.6711 | 2.5672 | 4.3254 | 3.6313 | 4.4492 | 6.1075 | 25 | 42 | 42 | 39 | 63 | 75 |
| TRBJ1-3 | 0.869 | 0.8557 | 0.7209 | 0.6518 | 0.8475 | 0.57 | 13 | 14 | 7 | 7 | 12 | 7 |
| TRBJ1-4 | 1.2701 | 1.1002 | 2.7806 | 1.9553 | 1.4124 | 1.873 | 19 | 18 | 27 | 21 | 20 | 23 |
| TRBJ1-5 | 3.4091 | 1.7115 | 3.8105 | 2.3277 | 2.4011 | 7.4104 | 51 | 28 | 37 | 25 | 34 | 91 |
| TRBJ1-6 | 4.2112 | 1.8337 | 3.8105 | 3.1657 | 5.6497 | 2.1173 | 63 | 30 | 37 | 34 | 80 | 26 |
| TRBJ2-1 | 25.4011 | 23.1051 | 17.9197 | 20.3911 | 24.8588 | 17.5896 | 380 | 378 | 174 | 219 | 352 | 216 |
| TRBJ2-2 | 6.885 | 9.1687 | 10.4016 | 6.7039 | 5.4379 | 6.2704 | 103 | 150 | 101 | 72 | 77 | 77 |
| TRBJ2-2P | 0 | 0.1222 | 0 | 0 | 0 | 0 | 0 | 2 | 0 | 0 | 0 | 0 |
| TRBJ2-3 | 7.0856 | 17.2372 | 15.654 | 25.0466 | 24.0113 | 21.9055 | 106 | 282 | 152 | 269 | 340 | 269 |
| TRBJ2-4 | 2.2727 | 2.7506 | 0.4119 | 2.2346 | 2.9661 | 2.1987 | 34 | 45 | 4 | 24 | 42 | 27 |
| TRBJ2-5 | 8.8235 | 12.5917 | 14.7271 | 9.311 | 9.322 | 7.2476 | 132 | 206 | 143 | 100 | 132 | 89 |
| TRBJ2-6 | 3.6096 | 1.0391 | 1.6478 | 1.676 | 0.7768 | 1.0586 | 54 | 17 | 16 | 18 | 11 | 13 |
| TRBJ2-7 | 20.4545 | 21.5159 | 15.757 | 17.2253 | 13.065 | 18.3225 | 306 | 352 | 153 | 185 | 185 | 225 |
