# Supplementary material for: High-throughput Treg cell receptor sequencing reveals differential immune repertoires in rheumatoid arthritis with kidney deficiency
Source: PeerJ. 2023 Feb 2;11:e14837. doi: 10.7717/peerj.14837 (PMC9899432; doi:10.7717/peerj.14837)
Supplement: Supplemental Information 9 — Fractions and frequencies of the VDJ combination for each sample are included in the dataset. [file peerj-11-14837-s009.docx]

**Supplement Table 5. AllSample VDJ Combination fraction and count**

| Gene | KD-01_Fraction | KD-02_Fraction | KD-03_Fraction | Non-KD-01_Fraction | Non-KD-02_Fraction | Non-KD-03_Fraction | KD-01_count | KD-02_count | KD-03_count | Non-KD-01_count | Non-KD-02_count | Non-KD-03_count |
| --- | --- | --- | --- | --- | --- | --- | --- | --- | --- | --- | --- | --- |
| TRBV10-1\|TRBD1\|TRBJ1-1 | 0.0668 | 0 | 0 | 0.0931 | 0 | 0.0814 | 1 | 0 | 0 | 1 | 0 | 1 |
| TRBV10-1\|TRBD1\|TRBJ1-4 | 0 | 0 | 0 | 0.0931 | 0 | 0 | 0 | 0 | 0 | 1 | 0 | 0 |
| TRBV10-1\|TRBD1\|TRBJ1-6 | 0 | 0 | 0 | 0 | 2.2599 | 0 | 0 | 0 | 0 | 0 | 32 | 0 |
| TRBV10-1\|TRBD1\|TRBJ2-1 | 0 | 0.1222 | 0 | 0 | 0 | 0 | 0 | 2 | 0 | 0 | 0 | 0 |
| TRBV10-1\|TRBD1\|TRBJ2-5 | 0.3342 | 0 | 0 | 0 | 0 | 0.0814 | 5 | 0 | 0 | 0 | 0 | 1 |
| TRBV10-1\|TRBD2\|TRBJ2-2 | 0 | 0 | 0 | 0 | 0 | 0.1629 | 0 | 0 | 0 | 0 | 0 | 2 |
| TRBV10-1\|TRBD2\|TRBJ2-3 | 0 | 0.1222 | 0 | 0 | 0 | 0 | 0 | 2 | 0 | 0 | 0 | 0 |
| TRBV10-1\|TRBD2\|TRBJ2-5 | 0 | 0 | 0.8239 | 0 | 0 | 0 | 0 | 0 | 8 | 0 | 0 | 0 |
| TRBV10-2\|TRBD1\|TRBJ1-1 | 0 | 0 | 0 | 0.0931 | 0 | 0 | 0 | 0 | 0 | 1 | 0 | 0 |
| TRBV10-2\|TRBD1\|TRBJ1-2 | 0.0668 | 0 | 0 | 0 | 0 | 0 | 1 | 0 | 0 | 0 | 0 | 0 |
| TRBV10-2\|TRBD1\|TRBJ1-5 | 0 | 0 | 0 | 0.0931 | 0.0706 | 0 | 0 | 0 | 0 | 1 | 1 | 0 |
| TRBV10-2\|TRBD1\|TRBJ2-1 | 0.2674 | 0 | 0 | 0 | 0 | 0 | 4 | 0 | 0 | 0 | 0 | 0 |
| TRBV10-2\|TRBD1\|TRBJ2-5 | 0 | 0 | 0.206 | 0.0931 | 0 | 0 | 0 | 0 | 2 | 1 | 0 | 0 |
| TRBV10-2\|TRBD1\|TRBJ2-6 | 0 | 0 | 0 | 0 | 0 | 0.0814 | 0 | 0 | 0 | 0 | 0 | 1 |
| TRBV10-2\|TRBD1\|TRBJ2-7 | 0 | 0 | 0.206 | 0 | 0 | 0 | 0 | 0 | 2 | 0 | 0 | 0 |
| TRBV10-2\|TRBD2\|TRBJ1-1 | 0 | 0 | 0 | 0 | 0 | 0.0814 | 0 | 0 | 0 | 0 | 0 | 1 |
| TRBV10-2\|TRBD2\|TRBJ2-1 | 0.4011 | 0.1222 | 0 | 0 | 0.0706 | 0 | 6 | 2 | 0 | 0 | 1 | 0 |
| TRBV10-2\|TRBD2\|TRBJ2-2 | 0 | 0 | 0 | 0.0931 | 0 | 0.0814 | 0 | 0 | 0 | 1 | 0 | 1 |
| TRBV10-2\|TRBD2\|TRBJ2-7 | 0 | 0 | 0.309 | 0.0931 | 0 | 0 | 0 | 0 | 3 | 1 | 0 | 0 |
| TRBV10-3\|TRBD1\|TRBJ1-1 | 0.0668 | 0 | 0.206 | 0.2793 | 0.1412 | 0.1629 | 1 | 0 | 2 | 3 | 2 | 2 |
| TRBV10-3\|TRBD1\|TRBJ1-2 | 0.0668 | 0 | 0 | 0.0931 | 0.0706 | 0 | 1 | 0 | 0 | 1 | 1 | 0 |
| TRBV10-3\|TRBD1\|TRBJ1-3 | 0.1337 | 0 | 0.309 | 0 | 0 | 0 | 2 | 0 | 3 | 0 | 0 | 0 |
| TRBV10-3\|TRBD1\|TRBJ1-4 | 0 | 0 | 0 | 0 | 0.0706 | 0 | 0 | 0 | 0 | 0 | 1 | 0 |
| TRBV10-3\|TRBD1\|TRBJ1-5 | 0.2005 | 0 | 0 | 0.0931 | 0.1412 | 0.0814 | 3 | 0 | 0 | 1 | 2 | 1 |
| TRBV10-3\|TRBD1\|TRBJ1-6 | 0 | 0 | 0 | 0 | 0.1412 | 0 | 0 | 0 | 0 | 0 | 2 | 0 |
| TRBV10-3\|TRBD1\|TRBJ2-1 | 0.0668 | 0.4279 | 1.1329 | 0.0931 | 0.3531 | 0 | 1 | 7 | 11 | 1 | 5 | 0 |
| TRBV10-3\|TRBD1\|TRBJ2-2 | 0 | 0 | 0.4119 | 0.0931 | 0.2119 | 0.3257 | 0 | 0 | 4 | 1 | 3 | 4 |
| TRBV10-3\|TRBD1\|TRBJ2-3 | 0.2674 | 0 | 0.4119 | 0.0931 | 0.0706 | 0.0814 | 4 | 0 | 4 | 1 | 1 | 1 |
| TRBV10-3\|TRBD1\|TRBJ2-4 | 0 | 0 | 0 | 0.1862 | 0 | 0 | 0 | 0 | 0 | 2 | 0 | 0 |
| TRBV10-3\|TRBD1\|TRBJ2-5 | 0.2005 | 0.2445 | 0.103 | 0.0931 | 0 | 0 | 3 | 4 | 1 | 1 | 0 | 0 |
| TRBV10-3\|TRBD1\|TRBJ2-6 | 0 | 0.0611 | 0 | 0 | 0 | 0 | 0 | 1 | 0 | 0 | 0 | 0 |
| TRBV10-3\|TRBD1\|TRBJ2-7 | 0.4011 | 0 | 0 | 0.2793 | 0.2825 | 0.1629 | 6 | 0 | 0 | 3 | 4 | 2 |
| TRBV10-3\|TRBD2\|TRBJ1-1 | 0 | 0 | 0.7209 | 0.1862 | 0 | 0 | 0 | 0 | 7 | 2 | 0 | 0 |
| TRBV10-3\|TRBD2\|TRBJ1-2 | 0 | 0 | 0 | 0 | 0.0706 | 0 | 0 | 0 | 0 | 0 | 1 | 0 |
| TRBV10-3\|TRBD2\|TRBJ1-4 | 0 | 0 | 0 | 0 | 0.1412 | 0 | 0 | 0 | 0 | 0 | 2 | 0 |
| TRBV10-3\|TRBD2\|TRBJ1-5 | 0.0668 | 0 | 0 | 0 | 0 | 0.0814 | 1 | 0 | 0 | 0 | 0 | 1 |
| TRBV10-3\|TRBD2\|TRBJ2-1 | 0.0668 | 0.6724 | 0 | 0.4655 | 0.4944 | 0.0814 | 1 | 11 | 0 | 5 | 7 | 1 |
| TRBV10-3\|TRBD2\|TRBJ2-2 | 0 | 0.0611 | 0 | 0 | 0.1412 | 0.1629 | 0 | 1 | 0 | 0 | 2 | 2 |
| TRBV10-3\|TRBD2\|TRBJ2-3 | 0.3342 | 0 | 0.309 | 0.0931 | 0.1412 | 0.2443 | 5 | 0 | 3 | 1 | 2 | 3 |
| TRBV10-3\|TRBD2\|TRBJ2-5 | 0 | 0.0611 | 0 | 0.0931 | 0 | 0.1629 | 0 | 1 | 0 | 1 | 0 | 2 |
| TRBV10-3\|TRBD2\|TRBJ2-6 | 0.0668 | 0 | 0 | 0 | 0 | 0 | 1 | 0 | 0 | 0 | 0 | 0 |
| TRBV10-3\|TRBD2\|TRBJ2-7 | 0.2005 | 0.3667 | 0 | 0 | 0.0706 | 0.4886 | 3 | 6 | 0 | 0 | 1 | 6 |
| TRBV11-1\|TRBD1\|TRBJ1-1 | 0 | 0 | 0 | 0 | 0.0706 | 0.0814 | 0 | 0 | 0 | 0 | 1 | 1 |
| TRBV11-1\|TRBD1\|TRBJ1-2 | 0 | 0 | 0 | 0 | 0.0706 | 0 | 0 | 0 | 0 | 0 | 1 | 0 |
| TRBV11-1\|TRBD1\|TRBJ2-3 | 0 | 0 | 0 | 0.0931 | 0.0706 | 0 | 0 | 0 | 0 | 1 | 1 | 0 |
| TRBV11-1\|TRBD1\|TRBJ2-5 | 0 | 0 | 0 | 0 | 0.0706 | 0 | 0 | 0 | 0 | 0 | 1 | 0 |
| TRBV11-1\|TRBD2\|TRBJ2-1 | 0 | 0 | 0 | 0.0931 | 0.0706 | 0.0814 | 0 | 0 | 0 | 1 | 1 | 1 |
| TRBV11-1\|TRBD2\|TRBJ2-7 | 0 | 0 | 0 | 0 | 0.0706 | 0 | 0 | 0 | 0 | 0 | 1 | 0 |
| TRBV11-2\|TRBD1\|TRBJ1-1 | 0 | 0.0611 | 0 | 0 | 0.1412 | 0.0814 | 0 | 1 | 0 | 0 | 2 | 1 |
| TRBV11-2\|TRBD1\|TRBJ1-3 | 0 | 0 | 0 | 0 | 0.1412 | 0 | 0 | 0 | 0 | 0 | 2 | 0 |
| TRBV11-2\|TRBD1\|TRBJ1-4 | 0 | 0.1222 | 0 | 0 | 0.0706 | 0 | 0 | 2 | 0 | 0 | 1 | 0 |
| TRBV11-2\|TRBD1\|TRBJ1-5 | 0 | 0 | 0 | 0 | 0 | 0.0814 | 0 | 0 | 0 | 0 | 0 | 1 |
| TRBV11-2\|TRBD1\|TRBJ1-6 | 0 | 0 | 0 | 0.0931 | 0.0706 | 0 | 0 | 0 | 0 | 1 | 1 | 0 |
| TRBV11-2\|TRBD1\|TRBJ2-1 | 0.1337 | 0.0611 | 0 | 0 | 0 | 0 | 2 | 1 | 0 | 0 | 0 | 0 |
| TRBV11-2\|TRBD1\|TRBJ2-2 | 0.0668 | 0.4279 | 0 | 0.0931 | 0.0706 | 0.0814 | 1 | 7 | 0 | 1 | 1 | 1 |
| TRBV11-2\|TRBD1\|TRBJ2-3 | 0 | 0 | 0 | 0.1862 | 0 | 0 | 0 | 0 | 0 | 2 | 0 | 0 |
| TRBV11-2\|TRBD1\|TRBJ2-4 | 0 | 0 | 0 | 0.0931 | 0 | 0.4072 | 0 | 0 | 0 | 1 | 0 | 5 |
| TRBV11-2\|TRBD1\|TRBJ2-5 | 0 | 0 | 0.206 | 0 | 0.2119 | 0 | 0 | 0 | 2 | 0 | 3 | 0 |
| TRBV11-2\|TRBD1\|TRBJ2-7 | 0 | 0.1834 | 0.103 | 0.1862 | 0.1412 | 0.0814 | 0 | 3 | 1 | 2 | 2 | 1 |
| TRBV11-2\|TRBD2\|TRBJ1-3 | 0 | 0 | 0.103 | 0 | 0.0706 | 0 | 0 | 0 | 1 | 0 | 1 | 0 |
| TRBV11-2\|TRBD2\|TRBJ1-4 | 0 | 0 | 0.5149 | 0 | 0.0706 | 0 | 0 | 0 | 5 | 0 | 1 | 0 |
| TRBV11-2\|TRBD2\|TRBJ1-6 | 0 | 0 | 0.206 | 0 | 0 | 0 | 0 | 0 | 2 | 0 | 0 | 0 |
| TRBV11-2\|TRBD2\|TRBJ2-1 | 0.4011 | 0.489 | 0.206 | 0.4655 | 0.4944 | 0.4072 | 6 | 8 | 2 | 5 | 7 | 5 |
| TRBV11-2\|TRBD2\|TRBJ2-2 | 0 | 0 | 0 | 0.1862 | 0.2119 | 0 | 0 | 0 | 0 | 2 | 3 | 0 |
| TRBV11-2\|TRBD2\|TRBJ2-3 | 0.1337 | 0.2445 | 0 | 0.1862 | 0 | 0 | 2 | 4 | 0 | 2 | 0 | 0 |
| TRBV11-2\|TRBD2\|TRBJ2-5 | 0.1337 | 0.1834 | 0 | 0 | 0.2119 | 0 | 2 | 3 | 0 | 0 | 3 | 0 |
| TRBV11-2\|TRBD2\|TRBJ2-7 | 0.4011 | 0.7946 | 0.206 | 0.0931 | 0.1412 | 0.1629 | 6 | 13 | 2 | 1 | 2 | 2 |
| TRBV11-3\|TRBD1\|TRBJ1-3 | 0.1337 | 0 | 0 | 0 | 0 | 0 | 2 | 0 | 0 | 0 | 0 | 0 |
| TRBV11-3\|TRBD1\|TRBJ1-5 | 0 | 0 | 0 | 0 | 0.0706 | 0 | 0 | 0 | 0 | 0 | 1 | 0 |
| TRBV11-3\|TRBD1\|TRBJ2-1 | 0 | 0 | 0 | 0.0931 | 0 | 0.0814 | 0 | 0 | 0 | 1 | 0 | 1 |
| TRBV11-3\|TRBD1\|TRBJ2-2 | 0 | 0 | 0 | 0 | 0.1412 | 0.0814 | 0 | 0 | 0 | 0 | 2 | 1 |
| TRBV11-3\|TRBD2\|TRBJ1-5 | 0 | 0 | 0 | 0.0931 | 0 | 0 | 0 | 0 | 0 | 1 | 0 | 0 |
| TRBV11-3\|TRBD2\|TRBJ2-1 | 0.1337 | 0 | 0.103 | 0 | 0 | 0 | 2 | 0 | 1 | 0 | 0 | 0 |
| TRBV11-3\|TRBD2\|TRBJ2-2 | 0.2005 | 0 | 0 | 0 | 0 | 0.0814 | 3 | 0 | 0 | 0 | 0 | 1 |
| TRBV11-3\|TRBD2\|TRBJ2-3 | 0 | 0 | 0 | 0 | 0.0706 | 0 | 0 | 0 | 0 | 0 | 1 | 0 |
| TRBV11-3\|TRBD2\|TRBJ2-4 | 0.1337 | 0 | 0 | 0 | 0 | 0 | 2 | 0 | 0 | 0 | 0 | 0 |
| TRBV11-3\|TRBD2\|TRBJ2-6 | 0 | 0 | 0 | 0.0931 | 0 | 0 | 0 | 0 | 0 | 1 | 0 | 0 |
| TRBV11-3\|TRBD2\|TRBJ2-7 | 0.0668 | 0 | 0 | 0 | 0 | 0 | 1 | 0 | 0 | 0 | 0 | 0 |
| TRBV12-2\|TRBD1\|TRBJ1-1 | 0 | 0 | 0 | 0.0931 | 0 | 0 | 0 | 0 | 0 | 1 | 0 | 0 |
| TRBV12-2\|TRBD1\|TRBJ1-5 | 0.1337 | 0 | 0 | 0 | 0.1412 | 0.0814 | 2 | 0 | 0 | 0 | 2 | 1 |
| TRBV12-2\|TRBD1\|TRBJ1-6 | 0 | 0 | 0 | 0 | 0 | 0.0814 | 0 | 0 | 0 | 0 | 0 | 1 |
| TRBV12-2\|TRBD1\|TRBJ2-1 | 0.2005 | 0 | 0 | 0 | 0 | 0 | 3 | 0 | 0 | 0 | 0 | 0 |
| TRBV12-2\|TRBD1\|TRBJ2-3 | 0 | 0 | 0 | 0 | 0.0706 | 0 | 0 | 0 | 0 | 0 | 1 | 0 |
| TRBV12-2\|TRBD1\|TRBJ2-5 | 0 | 0 | 0 | 0.0931 | 0.0706 | 0 | 0 | 0 | 0 | 1 | 1 | 0 |
| TRBV12-2\|TRBD1\|TRBJ2-7 | 0 | 0 | 0 | 0.1862 | 0.1412 | 0 | 0 | 0 | 0 | 2 | 2 | 0 |
| TRBV12-2\|TRBD2\|TRBJ2-3 | 0 | 0 | 0 | 0 | 0.0706 | 0 | 0 | 0 | 0 | 0 | 1 | 0 |
| TRBV12-2\|TRBD2\|TRBJ2-7 | 0.0668 | 0 | 0 | 0 | 0 | 0 | 1 | 0 | 0 | 0 | 0 | 0 |
| TRBV12-3\|TRBD1\|TRBJ1-1 | 0.1337 | 0.3667 | 0.103 | 0.5587 | 0.2119 | 0 | 2 | 6 | 1 | 6 | 3 | 0 |
| TRBV12-3\|TRBD1\|TRBJ1-2 | 0.0668 | 0.1834 | 0.103 | 0.1862 | 0.2825 | 0.4072 | 1 | 3 | 1 | 2 | 4 | 5 |
| TRBV12-3\|TRBD1\|TRBJ1-3 | 0.0668 | 0 | 0 | 0 | 0.0706 | 0 | 1 | 0 | 0 | 0 | 1 | 0 |
| TRBV12-3\|TRBD1\|TRBJ1-4 | 0.3342 | 0 | 0 | 0 | 0.0706 | 0 | 5 | 0 | 0 | 0 | 1 | 0 |
| TRBV12-3\|TRBD1\|TRBJ1-5 | 0.0668 | 0 | 0 | 0 | 0.0706 | 0.0814 | 1 | 0 | 0 | 0 | 1 | 1 |
| TRBV12-3\|TRBD1\|TRBJ1-6 | 0 | 0 | 0.206 | 0.1862 | 0 | 0.0814 | 0 | 0 | 2 | 2 | 0 | 1 |
| TRBV12-3\|TRBD1\|TRBJ2-1 | 1.0027 | 0.2445 | 0.206 | 0.4655 | 0.4237 | 0.0814 | 15 | 4 | 2 | 5 | 6 | 1 |
| TRBV12-3\|TRBD1\|TRBJ2-2 | 0.2005 | 0.8557 | 0 | 0.1862 | 0.3531 | 0.3257 | 3 | 14 | 0 | 2 | 5 | 4 |
| TRBV12-3\|TRBD1\|TRBJ2-3 | 0.1337 | 0.0611 | 0.206 | 0.2793 | 0.1412 | 0.2443 | 2 | 1 | 2 | 3 | 2 | 3 |
| TRBV12-3\|TRBD1\|TRBJ2-4 | 0.1337 | 0.2445 | 0 | 0.0931 | 0.2119 | 0 | 2 | 4 | 0 | 1 | 3 | 0 |
| TRBV12-3\|TRBD1\|TRBJ2-5 | 0.2674 | 0.9169 | 0 | 0.1862 | 0.1412 | 0 | 4 | 15 | 0 | 2 | 2 | 0 |
| TRBV12-3\|TRBD1\|TRBJ2-6 | 0.2674 | 0 | 0 | 0.0931 | 0.1412 | 0 | 4 | 0 | 0 | 1 | 2 | 0 |
| TRBV12-3\|TRBD1\|TRBJ2-7 | 0.869 | 0.7335 | 0.4119 | 0.838 | 0.2825 | 0.3257 | 13 | 12 | 4 | 9 | 4 | 4 |
| TRBV12-3\|TRBD2\|TRBJ1-1 | 0 | 0.0611 | 0 | 0.2793 | 0.0706 | 0.0814 | 0 | 1 | 0 | 3 | 1 | 1 |
| TRBV12-3\|TRBD2\|TRBJ1-2 | 0.0668 | 0.0611 | 0 | 0.0931 | 0 | 0.0814 | 1 | 1 | 0 | 1 | 0 | 1 |
| TRBV12-3\|TRBD2\|TRBJ1-3 | 0 | 0.1222 | 0 | 0 | 0 | 0 | 0 | 2 | 0 | 0 | 0 | 0 |
| TRBV12-3\|TRBD2\|TRBJ1-4 | 0 | 0 | 0 | 0.1862 | 0 | 0.0814 | 0 | 0 | 0 | 2 | 0 | 1 |
| TRBV12-3\|TRBD2\|TRBJ1-5 | 0.2674 | 0.0611 | 0.206 | 0 | 0 | 0 | 4 | 1 | 2 | 0 | 0 | 0 |
| TRBV12-3\|TRBD2\|TRBJ2-1 | 2.2059 | 0.4279 | 0 | 0.4655 | 0.7062 | 0.3257 | 33 | 7 | 0 | 5 | 10 | 4 |
| TRBV12-3\|TRBD2\|TRBJ2-2 | 0.0668 | 0 | 0 | 0.3724 | 0.2825 | 0 | 1 | 0 | 0 | 4 | 4 | 0 |
| TRBV12-3\|TRBD2\|TRBJ2-3 | 0.0668 | 0.3667 | 0.5149 | 0.2793 | 0.3531 | 0 | 1 | 6 | 5 | 3 | 5 | 0 |
| TRBV12-3\|TRBD2\|TRBJ2-4 | 0 | 0.0611 | 0 | 0 | 0.1412 | 0 | 0 | 1 | 0 | 0 | 2 | 0 |
| TRBV12-3\|TRBD2\|TRBJ2-5 | 0.3342 | 0.489 | 0.5149 | 0 | 0.2119 | 0.1629 | 5 | 8 | 5 | 0 | 3 | 2 |
| TRBV12-3\|TRBD2\|TRBJ2-6 | 0 | 0 | 0 | 0.0931 | 0 | 0 | 0 | 0 | 0 | 1 | 0 | 0 |
| TRBV12-3\|TRBD2\|TRBJ2-7 | 2.0722 | 1.467 | 0.103 | 1.3035 | 0.6356 | 0.2443 | 31 | 24 | 1 | 14 | 9 | 3 |
| TRBV12-4\|TRBD1\|TRBJ1-1 | 0 | 0.0611 | 0.206 | 0 | 0.1412 | 0 | 0 | 1 | 2 | 0 | 2 | 0 |
| TRBV12-4\|TRBD1\|TRBJ1-2 | 0 | 0.1834 | 0 | 0 | 0 | 0 | 0 | 3 | 0 | 0 | 0 | 0 |
| TRBV12-4\|TRBD1\|TRBJ2-2 | 0 | 0.1834 | 0 | 0 | 0 | 0.0814 | 0 | 3 | 0 | 0 | 0 | 1 |
| TRBV12-4\|TRBD1\|TRBJ2-7 | 0.0668 | 0 | 0 | 0 | 0 | 0 | 1 | 0 | 0 | 0 | 0 | 0 |
| TRBV12-4\|TRBD2\|TRBJ1-6 | 0 | 0 | 0 | 0.0931 | 0 | 0 | 0 | 0 | 0 | 1 | 0 | 0 |
| TRBV12-4\|TRBD2\|TRBJ2-1 | 0 | 0 | 0 | 0.0931 | 0 | 0 | 0 | 0 | 0 | 1 | 0 | 0 |
| TRBV12-4\|TRBD2\|TRBJ2-3 | 0 | 0 | 0 | 0 | 0.0706 | 0 | 0 | 0 | 0 | 0 | 1 | 0 |
| TRBV12-4\|TRBD2\|TRBJ2-5 | 0 | 0.0611 | 0 | 0 | 0 | 0 | 0 | 1 | 0 | 0 | 0 | 0 |
| TRBV12-4\|TRBD2\|TRBJ2-7 | 0 | 0 | 0 | 0.0931 | 0.0706 | 0 | 0 | 0 | 0 | 1 | 1 | 0 |
| TRBV12-5\|TRBD1\|TRBJ2-1 | 0.0668 | 0 | 0 | 0 | 0 | 0 | 1 | 0 | 0 | 0 | 0 | 0 |
| TRBV12-5\|TRBD1\|TRBJ2-7 | 0.0668 | 0 | 0 | 0 | 0 | 0 | 1 | 0 | 0 | 0 | 0 | 0 |
| TRBV12-5\|TRBD2\|TRBJ2-2 | 0 | 0 | 0 | 0.0931 | 0 | 0 | 0 | 0 | 0 | 1 | 0 | 0 |
| TRBV13\|TRBD1\|TRBJ1-2 | 0 | 0 | 0 | 0.0931 | 0 | 0.0814 | 0 | 0 | 0 | 1 | 0 | 1 |
| TRBV13\|TRBD1\|TRBJ1-3 | 0.0668 | 0 | 0.103 | 0 | 0.0706 | 0.1629 | 1 | 0 | 1 | 0 | 1 | 2 |
| TRBV13\|TRBD1\|TRBJ1-4 | 0 | 0.0611 | 0 | 0.0931 | 0.0706 | 0 | 0 | 1 | 0 | 1 | 1 | 0 |
| TRBV13\|TRBD1\|TRBJ1-5 | 0.1337 | 0 | 0 | 0 | 0 | 0 | 2 | 0 | 0 | 0 | 0 | 0 |
| TRBV13\|TRBD1\|TRBJ1-6 | 2.6738 | 1.1614 | 0.4119 | 1.9553 | 1.7655 | 0.3257 | 40 | 19 | 4 | 21 | 25 | 4 |
| TRBV13\|TRBD1\|TRBJ2-2 | 0.4679 | 0.0611 | 0.309 | 0.9311 | 0.4237 | 0.1629 | 7 | 1 | 3 | 10 | 6 | 2 |
| TRBV13\|TRBD1\|TRBJ2-3 | 0 | 6.5403 | 5.4583 | 14.7114 | 10.3107 | 9.6906 | 0 | 107 | 53 | 158 | 146 | 119 |
| TRBV13\|TRBD1\|TRBJ2-5 | 0 | 0 | 0.206 | 0 | 0 | 0 | 0 | 0 | 2 | 0 | 0 | 0 |
| TRBV13\|TRBD1\|TRBJ2-7 | 0 | 0.1222 | 0 | 0 | 0 | 0 | 0 | 2 | 0 | 0 | 0 | 0 |
| TRBV13\|TRBD2\|TRBJ1-5 | 0 | 0 | 0 | 0 | 0.0706 | 0 | 0 | 0 | 0 | 0 | 1 | 0 |
| TRBV13\|TRBD2\|TRBJ2-1 | 0 | 0 | 0 | 0.0931 | 0 | 0 | 0 | 0 | 0 | 1 | 0 | 0 |
| TRBV13\|TRBD2\|TRBJ2-3 | 0 | 0 | 0 | 0 | 0.0706 | 0 | 0 | 0 | 0 | 0 | 1 | 0 |
| TRBV13\|TRBD2\|TRBJ2-7 | 0.1337 | 0.3056 | 0 | 0.0931 | 0 | 0 | 2 | 5 | 0 | 1 | 0 | 0 |
| TRBV14\|TRBD1\|TRBJ1-1 | 0.1337 | 0.1834 | 0 | 0 | 0 | 0 | 2 | 3 | 0 | 0 | 0 | 0 |
| TRBV14\|TRBD1\|TRBJ1-2 | 0 | 0.0611 | 0 | 0 | 0 | 0 | 0 | 1 | 0 | 0 | 0 | 0 |
| TRBV14\|TRBD1\|TRBJ2-1 | 0 | 0 | 0 | 0 | 0.0706 | 0.0814 | 0 | 0 | 0 | 0 | 1 | 1 |
| TRBV14\|TRBD1\|TRBJ2-5 | 0 | 0 | 0.4119 | 0.0931 | 0 | 0 | 0 | 0 | 4 | 1 | 0 | 0 |
| TRBV14\|TRBD1\|TRBJ2-7 | 0.0668 | 0.1834 | 0 | 0.1862 | 0.1412 | 0 | 1 | 3 | 0 | 2 | 2 | 0 |
| TRBV14\|TRBD2\|TRBJ1-1 | 0 | 0 | 0 | 0 | 0.0706 | 0 | 0 | 0 | 0 | 0 | 1 | 0 |
| TRBV14\|TRBD2\|TRBJ2-1 | 0.1337 | 0.1834 | 0 | 0.2793 | 0 | 0.4886 | 2 | 3 | 0 | 3 | 0 | 6 |
| TRBV14\|TRBD2\|TRBJ2-2 | 0 | 0 | 0 | 0.0931 | 0 | 0 | 0 | 0 | 0 | 1 | 0 | 0 |
| TRBV14\|TRBD2\|TRBJ2-3 | 0.0668 | 0 | 0 | 0 | 0 | 0.0814 | 1 | 0 | 0 | 0 | 0 | 1 |
| TRBV14\|TRBD2\|TRBJ2-4 | 0 | 0.0611 | 0 | 0 | 0 | 0 | 0 | 1 | 0 | 0 | 0 | 0 |
| TRBV14\|TRBD2\|TRBJ2-5 | 0 | 0 | 0 | 0.0931 | 0 | 0 | 0 | 0 | 0 | 1 | 0 | 0 |
| TRBV14\|TRBD2\|TRBJ2-7 | 0 | 0.0611 | 0 | 0 | 0 | 0 | 0 | 1 | 0 | 0 | 0 | 0 |
| TRBV15\|TRBD1\|TRBJ1-1 | 0.0668 | 0 | 0 | 0.0931 | 0 | 0.0814 | 1 | 0 | 0 | 1 | 0 | 1 |
| TRBV15\|TRBD1\|TRBJ1-2 | 0.1337 | 0.1222 | 0 | 0 | 0 | 0.0814 | 2 | 2 | 0 | 0 | 0 | 1 |
| TRBV15\|TRBD1\|TRBJ1-3 | 0 | 0 | 0 | 0.1862 | 0 | 0 | 0 | 0 | 0 | 2 | 0 | 0 |
| TRBV15\|TRBD1\|TRBJ1-4 | 0 | 0.1222 | 0 | 0 | 0.1412 | 0.57 | 0 | 2 | 0 | 0 | 2 | 7 |
| TRBV15\|TRBD1\|TRBJ1-6 | 0 | 0 | 0 | 0 | 0.0706 | 0.2443 | 0 | 0 | 0 | 0 | 1 | 3 |
| TRBV15\|TRBD1\|TRBJ2-1 | 0.2674 | 0.3667 | 0 | 0.1862 | 0.0706 | 0.0814 | 4 | 6 | 0 | 2 | 1 | 1 |
| TRBV15\|TRBD1\|TRBJ2-2 | 0.1337 | 0 | 0 | 0 | 0.0706 | 0 | 2 | 0 | 0 | 0 | 1 | 0 |
| TRBV15\|TRBD1\|TRBJ2-3 | 0 | 0.1222 | 0 | 0 | 0 | 0 | 0 | 2 | 0 | 0 | 0 | 0 |
| TRBV15\|TRBD1\|TRBJ2-4 | 0.0668 | 0 | 0 | 0.0931 | 0 | 0 | 1 | 0 | 0 | 1 | 0 | 0 |
| TRBV15\|TRBD1\|TRBJ2-5 | 0.4011 | 0.6724 | 1.0299 | 0.0931 | 0.2119 | 0.2443 | 6 | 11 | 10 | 1 | 3 | 3 |
| TRBV15\|TRBD1\|TRBJ2-6 | 0.1337 | 0 | 0 | 0 | 0 | 0 | 2 | 0 | 0 | 0 | 0 | 0 |
| TRBV15\|TRBD1\|TRBJ2-7 | 0.1337 | 0 | 0.5149 | 0 | 0 | 0 | 2 | 0 | 5 | 0 | 0 | 0 |
| TRBV15\|TRBD2\|TRBJ1-1 | 0 | 0 | 0 | 0 | 0.0706 | 0 | 0 | 0 | 0 | 0 | 1 | 0 |
| TRBV15\|TRBD2\|TRBJ1-2 | 0 | 0 | 0 | 0 | 0.0706 | 0 | 0 | 0 | 0 | 0 | 1 | 0 |
| TRBV15\|TRBD2\|TRBJ1-4 | 0 | 0 | 0 | 0 | 0.0706 | 0 | 0 | 0 | 0 | 0 | 1 | 0 |
| TRBV15\|TRBD2\|TRBJ1-5 | 0 | 0 | 1.1329 | 0 | 0 | 0 | 0 | 0 | 11 | 0 | 0 | 0 |
| TRBV15\|TRBD2\|TRBJ1-6 | 0 | 0 | 0 | 0 | 0 | 0.0814 | 0 | 0 | 0 | 0 | 0 | 1 |
| TRBV15\|TRBD2\|TRBJ2-1 | 0.6684 | 0.978 | 0 | 0.2793 | 0.2825 | 0.57 | 10 | 16 | 0 | 3 | 4 | 7 |
| TRBV15\|TRBD2\|TRBJ2-2 | 0.8021 | 0 | 0 | 0.0931 | 0 | 0 | 12 | 0 | 0 | 1 | 0 | 0 |
| TRBV15\|TRBD2\|TRBJ2-3 | 0 | 0 | 0.103 | 0.1862 | 0.2119 | 0 | 0 | 0 | 1 | 2 | 3 | 0 |
| TRBV15\|TRBD2\|TRBJ2-4 | 0.0668 | 0.0611 | 0 | 0 | 0 | 0 | 1 | 1 | 0 | 0 | 0 | 0 |
| TRBV15\|TRBD2\|TRBJ2-5 | 0 | 0.1222 | 0 | 0.2793 | 0.2119 | 0.0814 | 0 | 2 | 0 | 3 | 3 | 1 |
| TRBV15\|TRBD2\|TRBJ2-7 | 0.1337 | 0 | 0 | 0.1862 | 0 | 0.0814 | 2 | 0 | 0 | 2 | 0 | 1 |
| TRBV16\|TRBD1\|TRBJ1-1 | 0 | 0.0611 | 0 | 0 | 0 | 0 | 0 | 1 | 0 | 0 | 0 | 0 |
| TRBV18\|TRBD1\|TRBJ1-1 | 0.2005 | 0.1222 | 0 | 0.0931 | 0.1412 | 0.4886 | 3 | 2 | 0 | 1 | 2 | 6 |
| TRBV18\|TRBD1\|TRBJ1-2 | 0.1337 | 0.1222 | 0.309 | 0 | 0 | 0.1629 | 2 | 2 | 3 | 0 | 0 | 2 |
| TRBV18\|TRBD1\|TRBJ1-3 | 0 | 0 | 0 | 0 | 0.0706 | 0.0814 | 0 | 0 | 0 | 0 | 1 | 1 |
| TRBV18\|TRBD1\|TRBJ1-4 | 0 | 0 | 0.103 | 0 | 0 | 0.0814 | 0 | 0 | 1 | 0 | 0 | 1 |
| TRBV18\|TRBD1\|TRBJ1-5 | 0.0668 | 0 | 0.206 | 0.0931 | 0.0706 | 0.0814 | 1 | 0 | 2 | 1 | 1 | 1 |
| TRBV18\|TRBD1\|TRBJ1-6 | 0 | 0 | 0.206 | 0 | 0 | 0.0814 | 0 | 0 | 2 | 0 | 0 | 1 |
| TRBV18\|TRBD1\|TRBJ2-1 | 0.3342 | 0.6724 | 0.103 | 0.3724 | 0.565 | 0.57 | 5 | 11 | 1 | 4 | 8 | 7 |
| TRBV18\|TRBD1\|TRBJ2-2 | 0.1337 | 0 | 0 | 0 | 0.1412 | 0 | 2 | 0 | 0 | 0 | 2 | 0 |
| TRBV18\|TRBD1\|TRBJ2-3 | 0.0668 | 0.1834 | 0.103 | 0.2793 | 0.1412 | 0.4886 | 1 | 3 | 1 | 3 | 2 | 6 |
| TRBV18\|TRBD1\|TRBJ2-4 | 0 | 0.0611 | 0 | 0.1862 | 0 | 0.1629 | 0 | 1 | 0 | 2 | 0 | 2 |
| TRBV18\|TRBD1\|TRBJ2-5 | 0.1337 | 0.3667 | 0 | 0.2793 | 0.0706 | 0.6515 | 2 | 6 | 0 | 3 | 1 | 8 |
| TRBV18\|TRBD1\|TRBJ2-6 | 0.0668 | 0 | 0.206 | 0 | 0 | 0 | 1 | 0 | 2 | 0 | 0 | 0 |
| TRBV18\|TRBD1\|TRBJ2-7 | 0.1337 | 0.3056 | 1.8538 | 0.3724 | 0.2119 | 0.6515 | 2 | 5 | 18 | 4 | 3 | 8 |
| TRBV18\|TRBD2\|TRBJ1-1 | 0.2005 | 0.2445 | 0 | 0 | 0.0706 | 0 | 3 | 4 | 0 | 0 | 1 | 0 |
| TRBV18\|TRBD2\|TRBJ1-5 | 0 | 0 | 0 | 0 | 0 | 0.1629 | 0 | 0 | 0 | 0 | 0 | 2 |
| TRBV18\|TRBD2\|TRBJ2-1 | 0.2674 | 0.0611 | 0 | 0.5587 | 0.3531 | 0.4072 | 4 | 1 | 0 | 6 | 5 | 5 |
| TRBV18\|TRBD2\|TRBJ2-2 | 0 | 0 | 0 | 0.0931 | 0 | 0 | 0 | 0 | 0 | 1 | 0 | 0 |
| TRBV18\|TRBD2\|TRBJ2-3 | 0 | 0.1834 | 0 | 0.2793 | 0 | 0.3257 | 0 | 3 | 0 | 3 | 0 | 4 |
| TRBV18\|TRBD2\|TRBJ2-4 | 0 | 0.1222 | 0 | 0 | 0 | 0 | 0 | 2 | 0 | 0 | 0 | 0 |
| TRBV18\|TRBD2\|TRBJ2-5 | 0.2005 | 0.0611 | 0 | 0.0931 | 0 | 0.0814 | 3 | 1 | 0 | 1 | 0 | 1 |
| TRBV18\|TRBD2\|TRBJ2-7 | 0.1337 | 0.8557 | 0.103 | 0.2793 | 0.2119 | 0 | 2 | 14 | 1 | 3 | 3 | 0 |
| TRBV19\|TRBD1\|TRBJ1-1 | 0.2005 | 0.2445 | 0.103 | 0 | 0 | 0.0814 | 3 | 4 | 1 | 0 | 0 | 1 |
| TRBV19\|TRBD1\|TRBJ1-2 | 0.2674 | 0.0611 | 0 | 0 | 0.1412 | 0.0814 | 4 | 1 | 0 | 0 | 2 | 1 |
| TRBV19\|TRBD1\|TRBJ1-4 | 0 | 0 | 0 | 0 | 0.0706 | 0 | 0 | 0 | 0 | 0 | 1 | 0 |
| TRBV19\|TRBD1\|TRBJ1-5 | 0 | 0.1834 | 0.103 | 0.1862 | 0.3531 | 0.3257 | 0 | 3 | 1 | 2 | 5 | 4 |
| TRBV19\|TRBD1\|TRBJ1-6 | 0 | 0 | 0.103 | 0 | 0.0706 | 0 | 0 | 0 | 1 | 0 | 1 | 0 |
| TRBV19\|TRBD1\|TRBJ2-1 | 0 | 0.1834 | 0 | 0.1862 | 0 | 0.0814 | 0 | 3 | 0 | 2 | 0 | 1 |
| TRBV19\|TRBD1\|TRBJ2-2 | 0.0668 | 0 | 0 | 0 | 0 | 0.0814 | 1 | 0 | 0 | 0 | 0 | 1 |
| TRBV19\|TRBD1\|TRBJ2-3 | 0.0668 | 0 | 0 | 0 | 0.0706 | 0 | 1 | 0 | 0 | 0 | 1 | 0 |
| TRBV19\|TRBD1\|TRBJ2-4 | 0.0668 | 0.0611 | 0 | 0.0931 | 0 | 0 | 1 | 1 | 0 | 1 | 0 | 0 |
| TRBV19\|TRBD1\|TRBJ2-7 | 0 | 0.4279 | 0.103 | 0.2793 | 0.1412 | 0 | 0 | 7 | 1 | 3 | 2 | 0 |
| TRBV19\|TRBD2\|TRBJ1-1 | 0 | 0.489 | 0.4119 | 0 | 0 | 0 | 0 | 8 | 4 | 0 | 0 | 0 |
| TRBV19\|TRBD2\|TRBJ1-2 | 0.0668 | 0 | 0 | 0 | 0.0706 | 0 | 1 | 0 | 0 | 0 | 1 | 0 |
| TRBV19\|TRBD2\|TRBJ1-5 | 0 | 0 | 0 | 0 | 0 | 1.7915 | 0 | 0 | 0 | 0 | 0 | 22 |
| TRBV19\|TRBD2\|TRBJ1-6 | 0.0668 | 0 | 0 | 0 | 0 | 0 | 1 | 0 | 0 | 0 | 0 | 0 |
| TRBV19\|TRBD2\|TRBJ2-1 | 0.1337 | 0.1834 | 0 | 0.0931 | 0.1412 | 0 | 2 | 3 | 0 | 1 | 2 | 0 |
| TRBV19\|TRBD2\|TRBJ2-2 | 0.0668 | 0 | 0 | 0 | 0.0706 | 0.0814 | 1 | 0 | 0 | 0 | 1 | 1 |
| TRBV19\|TRBD2\|TRBJ2-3 | 0.0668 | 0 | 0 | 0 | 0 | 0.3257 | 1 | 0 | 0 | 0 | 0 | 4 |
| TRBV19\|TRBD2\|TRBJ2-4 | 0 | 0 | 0 | 0 | 0 | 0.0814 | 0 | 0 | 0 | 0 | 0 | 1 |
| TRBV19\|TRBD2\|TRBJ2-5 | 0.0668 | 0.0611 | 0 | 0 | 0 | 0 | 1 | 1 | 0 | 0 | 0 | 0 |
| TRBV19\|TRBD2\|TRBJ2-7 | 0 | 0.1222 | 0 | 0.2793 | 0.1412 | 0.0814 | 0 | 2 | 0 | 3 | 2 | 1 |
| TRBV1\|TRBD2\|TRBJ1-1 | 0 | 0.1222 | 0 | 0 | 0 | 0 | 0 | 2 | 0 | 0 | 0 | 0 |
| TRBV1\|TRBD2\|TRBJ2-1 | 0 | 0 | 0 | 0.0931 | 0 | 0 | 0 | 0 | 0 | 1 | 0 | 0 |
| TRBV20-1\|TRBD1\|TRBJ1-1 | 0.0668 | 0.1222 | 0.4119 | 0.9311 | 0.8475 | 3.013 | 1 | 2 | 4 | 10 | 12 | 37 |
| TRBV20-1\|TRBD1\|TRBJ1-2 | 0.2674 | 0.3667 | 1.1329 | 0.5587 | 0.6356 | 2.0358 | 4 | 6 | 11 | 6 | 9 | 25 |
| TRBV20-1\|TRBD1\|TRBJ1-3 | 0.0668 | 0.0611 | 0 | 0 | 0.0706 | 0.1629 | 1 | 1 | 0 | 0 | 1 | 2 |
| TRBV20-1\|TRBD1\|TRBJ1-4 | 0.0668 | 0.3056 | 0.206 | 0 | 0.1412 | 0.3257 | 1 | 5 | 2 | 0 | 2 | 4 |
| TRBV20-1\|TRBD1\|TRBJ1-5 | 0.2005 | 0.1222 | 0.9269 | 0.3724 | 0 | 1.873 | 3 | 2 | 9 | 4 | 0 | 23 |
| TRBV20-1\|TRBD1\|TRBJ1-6 | 0.2005 | 0.1222 | 0.4119 | 0.1862 | 0.4944 | 0.0814 | 3 | 2 | 4 | 2 | 7 | 1 |
| TRBV20-1\|TRBD1\|TRBJ2-1 | 0.4011 | 1.1614 | 2.1627 | 0.6518 | 0.9887 | 0.8143 | 6 | 19 | 21 | 7 | 14 | 10 |
| TRBV20-1\|TRBD1\|TRBJ2-2 | 0.2005 | 0.1222 | 1.1329 | 0.4655 | 0.3531 | 0.3257 | 3 | 2 | 11 | 5 | 5 | 4 |
| TRBV20-1\|TRBD1\|TRBJ2-3 | 0.0668 | 0.489 | 0.5149 | 0.2793 | 1.1299 | 0.57 | 1 | 8 | 5 | 3 | 16 | 7 |
| TRBV20-1\|TRBD1\|TRBJ2-4 | 0.4011 | 0.3056 | 0 | 0.0931 | 0.0706 | 0 | 6 | 5 | 0 | 1 | 1 | 0 |
| TRBV20-1\|TRBD1\|TRBJ2-5 | 0.4679 | 0.3056 | 1.4418 | 0.9311 | 0.4944 | 0.4072 | 7 | 5 | 14 | 10 | 7 | 5 |
| TRBV20-1\|TRBD1\|TRBJ2-6 | 0.0668 | 0 | 0 | 0.1862 | 0.0706 | 0.0814 | 1 | 0 | 0 | 2 | 1 | 1 |
| TRBV20-1\|TRBD1\|TRBJ2-7 | 2.4733 | 2.0782 | 2.4717 | 1.2104 | 2.7542 | 2.1173 | 37 | 34 | 24 | 13 | 39 | 26 |
| TRBV20-1\|TRBD2\|TRBJ1-1 | 0.1337 | 0 | 0 | 0.2793 | 0.2119 | 0.3257 | 2 | 0 | 0 | 3 | 3 | 4 |
| TRBV20-1\|TRBD2\|TRBJ1-2 | 0 | 0.0611 | 0.103 | 0.2793 | 0.3531 | 0.1629 | 0 | 1 | 1 | 3 | 5 | 2 |
| TRBV20-1\|TRBD2\|TRBJ1-3 | 0 | 0 | 0 | 0.0931 | 0 | 0 | 0 | 0 | 0 | 1 | 0 | 0 |
| TRBV20-1\|TRBD2\|TRBJ1-4 | 0 | 0 | 0.206 | 0.1862 | 0 | 0.0814 | 0 | 0 | 2 | 2 | 0 | 1 |
| TRBV20-1\|TRBD2\|TRBJ1-5 | 0.6016 | 0.1834 | 0.206 | 0.2793 | 0.1412 | 0.2443 | 9 | 3 | 2 | 3 | 2 | 3 |
| TRBV20-1\|TRBD2\|TRBJ1-6 | 0.0668 | 0 | 0 | 0.0931 | 0.0706 | 0.3257 | 1 | 0 | 0 | 1 | 1 | 4 |
| TRBV20-1\|TRBD2\|TRBJ2-1 | 3.8102 | 3.4841 | 4.5314 | 1.5829 | 3.1073 | 2.3616 | 57 | 57 | 44 | 17 | 44 | 29 |
| TRBV20-1\|TRBD2\|TRBJ2-2 | 0.5348 | 0.4279 | 1.3388 | 0.5587 | 0.1412 | 1.0586 | 8 | 7 | 13 | 6 | 2 | 13 |
| TRBV20-1\|TRBD2\|TRBJ2-3 | 1.3369 | 1.5281 | 1.0299 | 0.6518 | 2.7542 | 2.5244 | 20 | 25 | 10 | 7 | 39 | 31 |
| TRBV20-1\|TRBD2\|TRBJ2-4 | 0.1337 | 0.3667 | 0 | 0.0931 | 0.2119 | 0.3257 | 2 | 6 | 0 | 1 | 3 | 4 |
| TRBV20-1\|TRBD2\|TRBJ2-5 | 1.2032 | 1.5892 | 3.3986 | 0.7449 | 0.9887 | 0.8958 | 18 | 26 | 33 | 8 | 14 | 11 |
| TRBV20-1\|TRBD2\|TRBJ2-6 | 0 | 0 | 0 | 0.0931 | 0 | 0 | 0 | 0 | 0 | 1 | 0 | 0 |
| TRBV20-1\|TRBD2\|TRBJ2-7 | 4.3449 | 2.8117 | 2.1627 | 2.0484 | 1.9068 | 3.0945 | 65 | 46 | 21 | 22 | 27 | 38 |
| TRBV20/OR9-2\|TRBD1\|TRBJ1-1 | 0.0668 | 0.0611 | 0.103 | 0.0931 | 0 | 0.1629 | 1 | 1 | 1 | 1 | 0 | 2 |
| TRBV20/OR9-2\|TRBD1\|TRBJ1-2 | 0.0668 | 0 | 0 | 0.0931 | 0.0706 | 0.1629 | 1 | 0 | 0 | 1 | 1 | 2 |
| TRBV20/OR9-2\|TRBD1\|TRBJ1-4 | 0 | 0 | 0 | 0.1862 | 0 | 0 | 0 | 0 | 0 | 2 | 0 | 0 |
| TRBV20/OR9-2\|TRBD1\|TRBJ1-5 | 0 | 0 | 0 | 0 | 0 | 0.0814 | 0 | 0 | 0 | 0 | 0 | 1 |
| TRBV20/OR9-2\|TRBD1\|TRBJ2-1 | 0.1337 | 0.0611 | 0.4119 | 0 | 0.1412 | 0.0814 | 2 | 1 | 4 | 0 | 2 | 1 |
| TRBV20/OR9-2\|TRBD1\|TRBJ2-2 | 0.0668 | 0 | 0 | 0 | 0 | 0 | 1 | 0 | 0 | 0 | 0 | 0 |
| TRBV20/OR9-2\|TRBD1\|TRBJ2-3 | 0 | 0.4279 | 0 | 0.0931 | 0 | 0 | 0 | 7 | 0 | 1 | 0 | 0 |
| TRBV20/OR9-2\|TRBD1\|TRBJ2-4 | 0 | 0 | 0 | 0.0931 | 0 | 0 | 0 | 0 | 0 | 1 | 0 | 0 |
| TRBV20/OR9-2\|TRBD1\|TRBJ2-5 | 0.0668 | 0.0611 | 0.206 | 0.2793 | 0.2119 | 0 | 1 | 1 | 2 | 3 | 3 | 0 |
| TRBV20/OR9-2\|TRBD1\|TRBJ2-6 | 0 | 0 | 0 | 0 | 0 | 0.0814 | 0 | 0 | 0 | 0 | 0 | 1 |
| TRBV20/OR9-2\|TRBD1\|TRBJ2-7 | 0 | 0.2445 | 0.6179 | 0.1862 | 0.1412 | 0 | 0 | 4 | 6 | 2 | 2 | 0 |
| TRBV20/OR9-2\|TRBD2\|TRBJ1-1 | 0 | 0.0611 | 0 | 0 | 0 | 0.0814 | 0 | 1 | 0 | 0 | 0 | 1 |
| TRBV20/OR9-2\|TRBD2\|TRBJ1-2 | 0 | 0.0611 | 0 | 0 | 0 | 0.1629 | 0 | 1 | 0 | 0 | 0 | 2 |
| TRBV20/OR9-2\|TRBD2\|TRBJ1-4 | 0 | 0 | 0 | 0.0931 | 0 | 0 | 0 | 0 | 0 | 1 | 0 | 0 |
| TRBV20/OR9-2\|TRBD2\|TRBJ1-5 | 0.0668 | 0 | 0 | 0 | 0 | 0 | 1 | 0 | 0 | 0 | 0 | 0 |
| TRBV20/OR9-2\|TRBD2\|TRBJ2-1 | 0 | 1.0391 | 0 | 0 | 0.2119 | 0 | 0 | 17 | 0 | 0 | 3 | 0 |
| TRBV20/OR9-2\|TRBD2\|TRBJ2-3 | 0.2005 | 0.0611 | 0.206 | 0.0931 | 0.0706 | 0.4072 | 3 | 1 | 2 | 1 | 1 | 5 |
| TRBV20/OR9-2\|TRBD2\|TRBJ2-4 | 0.0668 | 0.0611 | 0 | 0 | 0 | 0 | 1 | 1 | 0 | 0 | 0 | 0 |
| TRBV20/OR9-2\|TRBD2\|TRBJ2-5 | 0.2674 | 0.1222 | 0.309 | 0.0931 | 0.2119 | 0.2443 | 4 | 2 | 3 | 1 | 3 | 3 |
| TRBV20/OR9-2\|TRBD2\|TRBJ2-7 | 0.2005 | 0.1834 | 0 | 0 | 0.0706 | 0.1629 | 3 | 3 | 0 | 0 | 1 | 2 |
| TRBV21-1\|TRBD1\|TRBJ1-3 | 0 | 0.0611 | 0 | 0 | 0 | 0 | 0 | 1 | 0 | 0 | 0 | 0 |
| TRBV21-1\|TRBD1\|TRBJ2-2 | 0 | 0 | 0.6179 | 0 | 0 | 0 | 0 | 0 | 6 | 0 | 0 | 0 |
| TRBV21-1\|TRBD2\|TRBJ1-5 | 0 | 0 | 0 | 0.0931 | 0 | 0 | 0 | 0 | 0 | 1 | 0 | 0 |
| TRBV21-1\|TRBD2\|TRBJ2-1 | 0 | 0 | 0 | 0 | 0.0706 | 0 | 0 | 0 | 0 | 0 | 1 | 0 |
| TRBV21-1\|TRBD2\|TRBJ2-3 | 0 | 0 | 0 | 0.0931 | 0.0706 | 0 | 0 | 0 | 0 | 1 | 1 | 0 |
| TRBV21-1\|TRBD2\|TRBJ2-5 | 0.0668 | 0 | 0 | 0 | 0 | 0 | 1 | 0 | 0 | 0 | 0 | 0 |
| TRBV21-1\|TRBD2\|TRBJ2-7 | 0 | 0 | 0 | 0 | 0.0706 | 0 | 0 | 0 | 0 | 0 | 1 | 0 |
| TRBV23-1\|TRBD1\|TRBJ1-1 | 0 | 0 | 0 | 0.0931 | 0 | 0 | 0 | 0 | 0 | 1 | 0 | 0 |
| TRBV23-1\|TRBD1\|TRBJ1-2 | 0 | 0 | 0 | 0.0931 | 0 | 0 | 0 | 0 | 0 | 1 | 0 | 0 |
| TRBV23-1\|TRBD1\|TRBJ2-1 | 0.0668 | 0 | 0 | 0.1862 | 0 | 0 | 1 | 0 | 0 | 2 | 0 | 0 |
| TRBV23-1\|TRBD1\|TRBJ2-2 | 0 | 1.7726 | 0 | 0 | 0 | 0 | 0 | 29 | 0 | 0 | 0 | 0 |
| TRBV23-1\|TRBD1\|TRBJ2-3 | 0 | 0.0611 | 0 | 0 | 0 | 0 | 0 | 1 | 0 | 0 | 0 | 0 |
| TRBV23-1\|TRBD1\|TRBJ2-7 | 0.0668 | 0 | 0 | 0.1862 | 0 | 0 | 1 | 0 | 0 | 2 | 0 | 0 |
| TRBV23-1\|TRBD2\|TRBJ2-1 | 0.0668 | 0 | 0 | 0 | 0 | 0 | 1 | 0 | 0 | 0 | 0 | 0 |
| TRBV23-1\|TRBD2\|TRBJ2-2 | 0 | 0 | 0 | 0 | 0.0706 | 0 | 0 | 0 | 0 | 0 | 1 | 0 |
| TRBV23-1\|TRBD2\|TRBJ2-3 | 0 | 0.5501 | 0 | 0 | 0 | 0.2443 | 0 | 9 | 0 | 0 | 0 | 3 |
| TRBV23-1\|TRBD2\|TRBJ2-5 | 0 | 0 | 0.9269 | 0 | 0.0706 | 0 | 0 | 0 | 9 | 0 | 1 | 0 |
| TRBV23-1\|TRBD2\|TRBJ2-7 | 0 | 0 | 0 | 0.0931 | 0.0706 | 0 | 0 | 0 | 0 | 1 | 1 | 0 |
| TRBV24-1\|TRBD1\|TRBJ1-4 | 0 | 0.0611 | 0 | 0 | 0 | 0 | 0 | 1 | 0 | 0 | 0 | 0 |
| TRBV24-1\|TRBD1\|TRBJ1-5 | 0.1337 | 0 | 0 | 0 | 0 | 0.1629 | 2 | 0 | 0 | 0 | 0 | 2 |
| TRBV24-1\|TRBD1\|TRBJ2-1 | 0.3342 | 0.1834 | 0.8239 | 0.1862 | 0.4944 | 0 | 5 | 3 | 8 | 2 | 7 | 0 |
| TRBV24-1\|TRBD1\|TRBJ2-2 | 0 | 0.1222 | 0 | 0 | 0.0706 | 0.0814 | 0 | 2 | 0 | 0 | 1 | 1 |
| TRBV24-1\|TRBD1\|TRBJ2-3 | 0 | 0 | 0 | 0 | 0.2119 | 0 | 0 | 0 | 0 | 0 | 3 | 0 |
| TRBV24-1\|TRBD1\|TRBJ2-5 | 0 | 0.3056 | 0 | 0 | 0 | 0 | 0 | 5 | 0 | 0 | 0 | 0 |
| TRBV24-1\|TRBD1\|TRBJ2-7 | 0.0668 | 0 | 0.206 | 0.0931 | 0 | 0 | 1 | 0 | 2 | 1 | 0 | 0 |
| TRBV24-1\|TRBD2\|TRBJ1-1 | 0.0668 | 0 | 0.103 | 0 | 0 | 0 | 1 | 0 | 1 | 0 | 0 | 0 |
| TRBV24-1\|TRBD2\|TRBJ1-2 | 0 | 0.0611 | 0 | 0 | 0 | 0 | 0 | 1 | 0 | 0 | 0 | 0 |
| TRBV24-1\|TRBD2\|TRBJ2-1 | 0.5348 | 0.4279 | 0.5149 | 0.1862 | 0.4944 | 0.0814 | 8 | 7 | 5 | 2 | 7 | 1 |
| TRBV24-1\|TRBD2\|TRBJ2-2 | 0 | 0 | 0 | 0 | 0.0706 | 0 | 0 | 0 | 0 | 0 | 1 | 0 |
| TRBV24-1\|TRBD2\|TRBJ2-3 | 0.0668 | 0 | 0.206 | 0 | 0 | 0 | 1 | 0 | 2 | 0 | 0 | 0 |
| TRBV24-1\|TRBD2\|TRBJ2-4 | 0.0668 | 0.0611 | 0 | 0 | 0 | 0 | 1 | 1 | 0 | 0 | 0 | 0 |
| TRBV24-1\|TRBD2\|TRBJ2-5 | 0 | 0.0611 | 0 | 0 | 0.0706 | 0 | 0 | 1 | 0 | 0 | 1 | 0 |
| TRBV24-1\|TRBD2\|TRBJ2-6 | 0 | 0 | 0 | 0.0931 | 0 | 0 | 0 | 0 | 0 | 1 | 0 | 0 |
| TRBV25-1\|TRBD1\|TRBJ1-1 | 0 | 0 | 0 | 0 | 0.0706 | 0.0814 | 0 | 0 | 0 | 0 | 1 | 1 |
| TRBV25-1\|TRBD1\|TRBJ1-4 | 0 | 0 | 0 | 0 | 0 | 0.0814 | 0 | 0 | 0 | 0 | 0 | 1 |
| TRBV25-1\|TRBD1\|TRBJ1-5 | 0.0668 | 0 | 0 | 0 | 0 | 0 | 1 | 0 | 0 | 0 | 0 | 0 |
| TRBV25-1\|TRBD1\|TRBJ2-1 | 0 | 0 | 0 | 0.1862 | 0.1412 | 0 | 0 | 0 | 0 | 2 | 2 | 0 |
| TRBV25-1\|TRBD1\|TRBJ2-2 | 0.0668 | 0 | 0 | 0 | 0 | 0 | 1 | 0 | 0 | 0 | 0 | 0 |
| TRBV25-1\|TRBD1\|TRBJ2-5 | 0 | 0.1222 | 0 | 0 | 0 | 0 | 0 | 2 | 0 | 0 | 0 | 0 |
| TRBV25-1\|TRBD1\|TRBJ2-7 | 0 | 0.1222 | 0 | 0.1862 | 0 | 0 | 0 | 2 | 0 | 2 | 0 | 0 |
| TRBV25-1\|TRBD2\|TRBJ1-1 | 0 | 0.1834 | 0 | 0 | 0 | 0 | 0 | 3 | 0 | 0 | 0 | 0 |
| TRBV25-1\|TRBD2\|TRBJ1-2 | 0.0668 | 0 | 0 | 0 | 0 | 0 | 1 | 0 | 0 | 0 | 0 | 0 |
| TRBV25-1\|TRBD2\|TRBJ1-3 | 0 | 0 | 0 | 0 | 0.0706 | 0 | 0 | 0 | 0 | 0 | 1 | 0 |
| TRBV25-1\|TRBD2\|TRBJ1-6 | 0.0668 | 0 | 0 | 0 | 0 | 0 | 1 | 0 | 0 | 0 | 0 | 0 |
| TRBV25-1\|TRBD2\|TRBJ2-1 | 0 | 0 | 0.8239 | 0.1862 | 0.0706 | 0 | 0 | 0 | 8 | 2 | 1 | 0 |
| TRBV25-1\|TRBD2\|TRBJ2-2 | 0.0668 | 0 | 0 | 0 | 0 | 0 | 1 | 0 | 0 | 0 | 0 | 0 |
| TRBV25-1\|TRBD2\|TRBJ2-5 | 0 | 0.1222 | 0 | 0.0931 | 0 | 0 | 0 | 2 | 0 | 1 | 0 | 0 |
| TRBV27\|TRBD1\|TRBJ1-1 | 0.3342 | 0 | 0 | 0 | 0 | 0 | 5 | 0 | 0 | 0 | 0 | 0 |
| TRBV27\|TRBD1\|TRBJ1-2 | 0.0668 | 0.1222 | 0.6179 | 0.0931 | 0.2119 | 0.1629 | 1 | 2 | 6 | 1 | 3 | 2 |
| TRBV27\|TRBD1\|TRBJ1-3 | 0 | 0 | 0 | 0 | 0.0706 | 0 | 0 | 0 | 0 | 0 | 1 | 0 |
| TRBV27\|TRBD1\|TRBJ1-4 | 0 | 0 | 0.9269 | 0.2793 | 0 | 0 | 0 | 0 | 9 | 3 | 0 | 0 |
| TRBV27\|TRBD1\|TRBJ1-5 | 0.2674 | 0 | 0 | 0.0931 | 0 | 0 | 4 | 0 | 0 | 1 | 0 | 0 |
| TRBV27\|TRBD1\|TRBJ1-6 | 0.0668 | 0 | 0.6179 | 0.0931 | 0 | 0.2443 | 1 | 0 | 6 | 1 | 0 | 3 |
| TRBV27\|TRBD1\|TRBJ2-1 | 0.4679 | 0.1222 | 0 | 0.9311 | 0.2119 | 0.2443 | 7 | 2 | 0 | 10 | 3 | 3 |
| TRBV27\|TRBD1\|TRBJ2-2 | 0 | 0.0611 | 0.206 | 0 | 0 | 0 | 0 | 1 | 2 | 0 | 0 | 0 |
| TRBV27\|TRBD1\|TRBJ2-3 | 0.3342 | 0.5501 | 0.309 | 0.1862 | 0 | 0 | 5 | 9 | 3 | 2 | 0 | 0 |
| TRBV27\|TRBD1\|TRBJ2-4 | 0 | 0.2445 | 0 | 0.2793 | 0 | 0 | 0 | 4 | 0 | 3 | 0 | 0 |
| TRBV27\|TRBD1\|TRBJ2-5 | 0 | 0.2445 | 0 | 0.2793 | 0.0706 | 0.1629 | 0 | 4 | 0 | 3 | 1 | 2 |
| TRBV27\|TRBD1\|TRBJ2-6 | 0.0668 | 0 | 0 | 0.0931 | 0.0706 | 0 | 1 | 0 | 0 | 1 | 1 | 0 |
| TRBV27\|TRBD1\|TRBJ2-7 | 0.1337 | 0.3667 | 0.4119 | 0.1862 | 0 | 0.0814 | 2 | 6 | 4 | 2 | 0 | 1 |
| TRBV27\|TRBD2\|TRBJ1-1 | 0.0668 | 0 | 0 | 0 | 0 | 0.0814 | 1 | 0 | 0 | 0 | 0 | 1 |
| TRBV27\|TRBD2\|TRBJ1-2 | 0 | 0.1222 | 0 | 0 | 0.0706 | 0 | 0 | 2 | 0 | 0 | 1 | 0 |
| TRBV27\|TRBD2\|TRBJ1-5 | 0.1337 | 0.0611 | 0 | 0.2793 | 0.4237 | 0 | 2 | 1 | 0 | 3 | 6 | 0 |
| TRBV27\|TRBD2\|TRBJ1-6 | 0 | 0 | 0 | 0 | 0 | 0.0814 | 0 | 0 | 0 | 0 | 0 | 1 |
| TRBV27\|TRBD2\|TRBJ2-1 | 0.1337 | 0.1222 | 0.8239 | 0.7449 | 0.2119 | 0.0814 | 2 | 2 | 8 | 8 | 3 | 1 |
| TRBV27\|TRBD2\|TRBJ2-2 | 0.2674 | 0.0611 | 0.103 | 0 | 0.0706 | 0.0814 | 4 | 1 | 1 | 0 | 1 | 1 |
| TRBV27\|TRBD2\|TRBJ2-3 | 0.0668 | 0.5501 | 0.5149 | 0.1862 | 0 | 0.2443 | 1 | 9 | 5 | 2 | 0 | 3 |
| TRBV27\|TRBD2\|TRBJ2-4 | 0 | 0.1222 | 0 | 0.1862 | 0 | 0.0814 | 0 | 2 | 0 | 2 | 0 | 1 |
| TRBV27\|TRBD2\|TRBJ2-5 | 0 | 0.1834 | 0 | 0.0931 | 0 | 0.2443 | 0 | 3 | 0 | 1 | 0 | 3 |
| TRBV27\|TRBD2\|TRBJ2-6 | 0 | 0 | 0 | 0 | 0 | 0.0814 | 0 | 0 | 0 | 0 | 0 | 1 |
| TRBV27\|TRBD2\|TRBJ2-7 | 0.0668 | 0.5501 | 0.206 | 0.2793 | 0 | 0.0814 | 1 | 9 | 2 | 3 | 0 | 1 |
| TRBV28\|TRBD1\|TRBJ1-1 | 0.0668 | 0.0611 | 0 | 0.0931 | 0 | 0.0814 | 1 | 1 | 0 | 1 | 0 | 1 |
| TRBV28\|TRBD1\|TRBJ1-2 | 0 | 0.0611 | 0.103 | 0.2793 | 0 | 0.1629 | 0 | 1 | 1 | 3 | 0 | 2 |
| TRBV28\|TRBD1\|TRBJ1-3 | 0 | 0.1222 | 0 | 0 | 0.0706 | 0 | 0 | 2 | 0 | 0 | 1 | 0 |
| TRBV28\|TRBD1\|TRBJ1-4 | 0 | 0 | 0 | 0.0931 | 0 | 0 | 0 | 0 | 0 | 1 | 0 | 0 |
| TRBV28\|TRBD1\|TRBJ1-5 | 0 | 0 | 0 | 0 | 0 | 0.0814 | 0 | 0 | 0 | 0 | 0 | 1 |
| TRBV28\|TRBD1\|TRBJ2-1 | 0.2674 | 0 | 0.309 | 0.0931 | 0.1412 | 0.0814 | 4 | 0 | 3 | 1 | 2 | 1 |
| TRBV28\|TRBD1\|TRBJ2-2 | 0.0668 | 0.2445 | 0.206 | 0.0931 | 0.0706 | 0 | 1 | 4 | 2 | 1 | 1 | 0 |
| TRBV28\|TRBD1\|TRBJ2-3 | 0.0668 | 0.489 | 0 | 0.0931 | 0 | 0.0814 | 1 | 8 | 0 | 1 | 0 | 1 |
| TRBV28\|TRBD1\|TRBJ2-4 | 0.0668 | 0.0611 | 0 | 0 | 0 | 0 | 1 | 1 | 0 | 0 | 0 | 0 |
| TRBV28\|TRBD1\|TRBJ2-5 | 0 | 0 | 0.103 | 0 | 0 | 0.3257 | 0 | 0 | 1 | 0 | 0 | 4 |
| TRBV28\|TRBD1\|TRBJ2-6 | 0.0668 | 0.2445 | 0 | 0.0931 | 0 | 0 | 1 | 4 | 0 | 1 | 0 | 0 |
| TRBV28\|TRBD1\|TRBJ2-7 | 0.3342 | 0 | 0 | 0.0931 | 0.0706 | 0.4886 | 5 | 0 | 0 | 1 | 1 | 6 |
| TRBV28\|TRBD2\|TRBJ1-1 | 0.0668 | 0.1222 | 0 | 0 | 0 | 0 | 1 | 2 | 0 | 0 | 0 | 0 |
| TRBV28\|TRBD2\|TRBJ1-2 | 0 | 0 | 0 | 0.0931 | 0 | 0.1629 | 0 | 0 | 0 | 1 | 0 | 2 |
| TRBV28\|TRBD2\|TRBJ1-3 | 0 | 0 | 0 | 0.0931 | 0 | 0 | 0 | 0 | 0 | 1 | 0 | 0 |
| TRBV28\|TRBD2\|TRBJ1-5 | 0.0668 | 0.0611 | 0 | 0 | 0 | 0 | 1 | 1 | 0 | 0 | 0 | 0 |
| TRBV28\|TRBD2\|TRBJ1-6 | 0.0668 | 0 | 0 | 0 | 0 | 0.0814 | 1 | 0 | 0 | 0 | 0 | 1 |
| TRBV28\|TRBD2\|TRBJ2-1 | 0.2005 | 0.3667 | 0.4119 | 0.3724 | 0.1412 | 0.0814 | 3 | 6 | 4 | 4 | 2 | 1 |
| TRBV28\|TRBD2\|TRBJ2-2 | 0.0668 | 0.1834 | 0 | 0.0931 | 0.0706 | 0 | 1 | 3 | 0 | 1 | 1 | 0 |
| TRBV28\|TRBD2\|TRBJ2-3 | 0.1337 | 0.1834 | 0 | 0.3724 | 0 | 0.1629 | 2 | 3 | 0 | 4 | 0 | 2 |
| TRBV28\|TRBD2\|TRBJ2-4 | 0 | 0 | 0 | 0.0931 | 0 | 0 | 0 | 0 | 0 | 1 | 0 | 0 |
| TRBV28\|TRBD2\|TRBJ2-5 | 0 | 0 | 0 | 0.0931 | 0 | 0.0814 | 0 | 0 | 0 | 1 | 0 | 1 |
| TRBV28\|TRBD2\|TRBJ2-6 | 0 | 0 | 0 | 0.0931 | 0 | 0 | 0 | 0 | 0 | 1 | 0 | 0 |
| TRBV28\|TRBD2\|TRBJ2-7 | 0 | 0 | 0 | 0.1862 | 0 | 0.1629 | 0 | 0 | 0 | 2 | 0 | 2 |
| TRBV29-1\|TRBD1\|TRBJ1-1 | 0.2674 | 0.1222 | 0 | 0.0931 | 0.2119 | 0.0814 | 4 | 2 | 0 | 1 | 3 | 1 |
| TRBV29-1\|TRBD1\|TRBJ1-2 | 0.0668 | 0.2445 | 0 | 0.1862 | 0.2825 | 0.3257 | 1 | 4 | 0 | 2 | 4 | 4 |
| TRBV29-1\|TRBD1\|TRBJ1-4 | 0 | 0.0611 | 0 | 0.1862 | 0 | 0 | 0 | 1 | 0 | 2 | 0 | 0 |
| TRBV29-1\|TRBD1\|TRBJ1-5 | 0.0668 | 0.1834 | 0 | 0 | 0 | 0.2443 | 1 | 3 | 0 | 0 | 0 | 3 |
| TRBV29-1\|TRBD1\|TRBJ2-1 | 0.5348 | 0.6724 | 0 | 0.3724 | 0.7062 | 0.3257 | 8 | 11 | 0 | 4 | 10 | 4 |
| TRBV29-1\|TRBD1\|TRBJ2-2 | 0 | 0.3056 | 0 | 0 | 0 | 0.1629 | 0 | 5 | 0 | 0 | 0 | 2 |
| TRBV29-1\|TRBD1\|TRBJ2-3 | 0.0668 | 0.0611 | 0.309 | 0.3724 | 0.7062 | 0.0814 | 1 | 1 | 3 | 4 | 10 | 1 |
| TRBV29-1\|TRBD1\|TRBJ2-4 | 0 | 0 | 0 | 0.0931 | 0.1412 | 0 | 0 | 0 | 0 | 1 | 2 | 0 |
| TRBV29-1\|TRBD1\|TRBJ2-5 | 0.4011 | 0.1222 | 0 | 0.3724 | 0.9887 | 0.0814 | 6 | 2 | 0 | 4 | 14 | 1 |
| TRBV29-1\|TRBD1\|TRBJ2-7 | 0.2674 | 0.5501 | 0.7209 | 0.5587 | 0.9181 | 1.3029 | 4 | 9 | 7 | 6 | 13 | 16 |
| TRBV29-1\|TRBD2\|TRBJ1-1 | 0.2674 | 0.0611 | 0 | 0 | 0 | 0.2443 | 4 | 1 | 0 | 0 | 0 | 3 |
| TRBV29-1\|TRBD2\|TRBJ1-2 | 0 | 0 | 0 | 0.0931 | 0.0706 | 0 | 0 | 0 | 0 | 1 | 1 | 0 |
| TRBV29-1\|TRBD2\|TRBJ1-4 | 0 | 0 | 0 | 0.0931 | 0 | 0 | 0 | 0 | 0 | 1 | 0 | 0 |
| TRBV29-1\|TRBD2\|TRBJ2-1 | 1.0027 | 1.3447 | 0 | 0.2793 | 0.8475 | 0.3257 | 15 | 22 | 0 | 3 | 12 | 4 |
| TRBV29-1\|TRBD2\|TRBJ2-2 | 0.2005 | 0.4279 | 0.8239 | 0 | 0.1412 | 0 | 3 | 7 | 8 | 0 | 2 | 0 |
| TRBV29-1\|TRBD2\|TRBJ2-3 | 0.2005 | 0.3056 | 1.1329 | 0.4655 | 1.2712 | 0 | 3 | 5 | 11 | 5 | 18 | 0 |
| TRBV29-1\|TRBD2\|TRBJ2-4 | 0 | 0.1222 | 0 | 0 | 1.1299 | 0 | 0 | 2 | 0 | 0 | 16 | 0 |
| TRBV29-1\|TRBD2\|TRBJ2-5 | 0.2005 | 0.1834 | 0 | 0.3724 | 0.2119 | 0.0814 | 3 | 3 | 0 | 4 | 3 | 1 |
| TRBV29-1\|TRBD2\|TRBJ2-7 | 1.0695 | 0.4279 | 0 | 0.4655 | 0.0706 | 0.57 | 16 | 7 | 0 | 5 | 1 | 7 |
| TRBV2\|TRBD1\|TRBJ1-1 | 0 | 0 | 0 | 0.0931 | 0 | 0 | 0 | 0 | 0 | 1 | 0 | 0 |
| TRBV2\|TRBD1\|TRBJ1-2 | 0 | 0 | 0.103 | 0.1862 | 0.1412 | 0 | 0 | 0 | 1 | 2 | 2 | 0 |
| TRBV2\|TRBD1\|TRBJ1-5 | 0.0668 | 0 | 0.103 | 0 | 0 | 0.0814 | 1 | 0 | 1 | 0 | 0 | 1 |
| TRBV2\|TRBD1\|TRBJ2-1 | 0.0668 | 0 | 0.206 | 0 | 0.0706 | 0.0814 | 1 | 0 | 2 | 0 | 1 | 1 |
| TRBV2\|TRBD1\|TRBJ2-2 | 0 | 0 | 0.7209 | 0.0931 | 0 | 0 | 0 | 0 | 7 | 1 | 0 | 0 |
| TRBV2\|TRBD1\|TRBJ2-3 | 0.1337 | 0.0611 | 0.103 | 0 | 0.1412 | 0.2443 | 2 | 1 | 1 | 0 | 2 | 3 |
| TRBV2\|TRBD1\|TRBJ2-4 | 0 | 0 | 0 | 0.0931 | 0 | 0 | 0 | 0 | 0 | 1 | 0 | 0 |
| TRBV2\|TRBD1\|TRBJ2-5 | 0 | 0.1834 | 0 | 0 | 0 | 0 | 0 | 3 | 0 | 0 | 0 | 0 |
| TRBV2\|TRBD1\|TRBJ2-6 | 0.0668 | 0.0611 | 0.309 | 0 | 0 | 0 | 1 | 1 | 3 | 0 | 0 | 0 |
| TRBV2\|TRBD1\|TRBJ2-7 | 0.1337 | 0.1834 | 0.6179 | 0 | 0.2825 | 0 | 2 | 3 | 6 | 0 | 4 | 0 |
| TRBV2\|TRBD2\|TRBJ1-5 | 0 | 0 | 0.103 | 0 | 0.0706 | 0 | 0 | 0 | 1 | 0 | 1 | 0 |
| TRBV2\|TRBD2\|TRBJ2-1 | 0.2005 | 0.1834 | 0 | 0.0931 | 0.6356 | 0.3257 | 3 | 3 | 0 | 1 | 9 | 4 |
| TRBV2\|TRBD2\|TRBJ2-2 | 0 | 0.1222 | 0 | 0 | 0.0706 | 0 | 0 | 2 | 0 | 0 | 1 | 0 |
| TRBV2\|TRBD2\|TRBJ2-3 | 0.1337 | 0 | 0 | 0.0931 | 0.2825 | 0.0814 | 2 | 0 | 0 | 1 | 4 | 1 |
| TRBV2\|TRBD2\|TRBJ2-4 | 0 | 0 | 0 | 0 | 0.0706 | 0 | 0 | 0 | 0 | 0 | 1 | 0 |
| TRBV2\|TRBD2\|TRBJ2-5 | 0 | 0.9169 | 0.4119 | 0 | 0 | 0 | 0 | 15 | 4 | 0 | 0 | 0 |
| TRBV2\|TRBD2\|TRBJ2-6 | 0 | 0.0611 | 0 | 0 | 0 | 0 | 0 | 1 | 0 | 0 | 0 | 0 |
| TRBV2\|TRBD2\|TRBJ2-7 | 0.1337 | 0 | 0 | 0.1862 | 0.2825 | 0.0814 | 2 | 0 | 0 | 2 | 4 | 1 |
| TRBV3-1\|TRBD1\|TRBJ1-1 | 0 | 0 | 0.206 | 0.0931 | 0.0706 | 0.2443 | 0 | 0 | 2 | 1 | 1 | 3 |
| TRBV3-1\|TRBD1\|TRBJ1-2 | 0 | 0 | 0 | 0.0931 | 0.2119 | 0 | 0 | 0 | 0 | 1 | 3 | 0 |
| TRBV3-1\|TRBD1\|TRBJ1-3 | 0 | 0 | 0 | 0.0931 | 0 | 0.0814 | 0 | 0 | 0 | 1 | 0 | 1 |
| TRBV3-1\|TRBD1\|TRBJ1-4 | 0.0668 | 0 | 0.103 | 0.0931 | 0.0706 | 0 | 1 | 0 | 1 | 1 | 1 | 0 |
| TRBV3-1\|TRBD1\|TRBJ1-5 | 0 | 0 | 0 | 0.0931 | 0.1412 | 0 | 0 | 0 | 0 | 1 | 2 | 0 |
| TRBV3-1\|TRBD1\|TRBJ1-6 | 0.0668 | 0 | 0 | 0 | 0 | 0 | 1 | 0 | 0 | 0 | 0 | 0 |
| TRBV3-1\|TRBD1\|TRBJ2-1 | 0.1337 | 0.0611 | 0 | 0.1862 | 0.1412 | 0.0814 | 2 | 1 | 0 | 2 | 2 | 1 |
| TRBV3-1\|TRBD1\|TRBJ2-2 | 0 | 0 | 0.309 | 0 | 0.0706 | 0.0814 | 0 | 0 | 3 | 0 | 1 | 1 |
| TRBV3-1\|TRBD1\|TRBJ2-3 | 0.0668 | 0.1834 | 0.5149 | 0 | 0.0706 | 0 | 1 | 3 | 5 | 0 | 1 | 0 |
| TRBV3-1\|TRBD1\|TRBJ2-5 | 0 | 0 | 0 | 0 | 0.0706 | 0 | 0 | 0 | 0 | 0 | 1 | 0 |
| TRBV3-1\|TRBD1\|TRBJ2-6 | 0 | 0 | 0 | 0.0931 | 0.0706 | 0 | 0 | 0 | 0 | 1 | 1 | 0 |
| TRBV3-1\|TRBD1\|TRBJ2-7 | 0.2005 | 0.3667 | 0.206 | 0.2793 | 0 | 0.8143 | 3 | 6 | 2 | 3 | 0 | 10 |
| TRBV3-1\|TRBD2\|TRBJ1-1 | 0 | 0.2445 | 0 | 0 | 0.1412 | 0 | 0 | 4 | 0 | 0 | 2 | 0 |
| TRBV3-1\|TRBD2\|TRBJ1-4 | 0.0668 | 0 | 0 | 0 | 0 | 0 | 1 | 0 | 0 | 0 | 0 | 0 |
| TRBV3-1\|TRBD2\|TRBJ1-6 | 0.0668 | 0 | 0 | 0 | 0.0706 | 0 | 1 | 0 | 0 | 0 | 1 | 0 |
| TRBV3-1\|TRBD2\|TRBJ2-1 | 0.4679 | 0.7946 | 0.206 | 0.1862 | 0.3531 | 0.8958 | 7 | 13 | 2 | 2 | 5 | 11 |
| TRBV3-1\|TRBD2\|TRBJ2-2 | 0.0668 | 0.0611 | 0.7209 | 0.1862 | 0 | 0 | 1 | 1 | 7 | 2 | 0 | 0 |
| TRBV3-1\|TRBD2\|TRBJ2-3 | 0 | 0.3667 | 0.206 | 0.0931 | 0.1412 | 0.57 | 0 | 6 | 2 | 1 | 2 | 7 |
| TRBV3-1\|TRBD2\|TRBJ2-4 | 0.0668 | 0 | 0 | 0.0931 | 0.1412 | 0.0814 | 1 | 0 | 0 | 1 | 2 | 1 |
| TRBV3-1\|TRBD2\|TRBJ2-5 | 0.0668 | 0 | 0 | 0.2793 | 0.2825 | 0 | 1 | 0 | 0 | 3 | 4 | 0 |
| TRBV3-1\|TRBD2\|TRBJ2-6 | 0 | 0.0611 | 0 | 0 | 0.0706 | 0.1629 | 0 | 1 | 0 | 0 | 1 | 2 |
| TRBV3-1\|TRBD2\|TRBJ2-7 | 0.4679 | 0.1222 | 0 | 0.0931 | 0 | 1.5472 | 7 | 2 | 0 | 1 | 0 | 19 |
| TRBV3-2\|TRBD2\|TRBJ2-5 | 0 | 0 | 0 | 0 | 0.0706 | 0 | 0 | 0 | 0 | 0 | 1 | 0 |
| TRBV30\|TRBD1\|TRBJ1-1 | 0.1337 | 0 | 0 | 0 | 0 | 0 | 2 | 0 | 0 | 0 | 0 | 0 |
| TRBV30\|TRBD1\|TRBJ1-2 | 0 | 0.0611 | 0 | 0 | 0 | 0.0814 | 0 | 1 | 0 | 0 | 0 | 1 |
| TRBV30\|TRBD1\|TRBJ1-4 | 0 | 0 | 0 | 0 | 0 | 0.0814 | 0 | 0 | 0 | 0 | 0 | 1 |
| TRBV30\|TRBD1\|TRBJ1-5 | 0.2005 | 0 | 0.4119 | 0 | 0 | 0 | 3 | 0 | 4 | 0 | 0 | 0 |
| TRBV30\|TRBD1\|TRBJ2-1 | 0.2005 | 0.0611 | 0 | 0 | 0.0706 | 0.4072 | 3 | 1 | 0 | 0 | 1 | 5 |
| TRBV30\|TRBD1\|TRBJ2-2 | 0 | 0.0611 | 0 | 0.0931 | 0 | 0.0814 | 0 | 1 | 0 | 1 | 0 | 1 |
| TRBV30\|TRBD1\|TRBJ2-4 | 0 | 0.0611 | 0 | 0 | 0 | 0 | 0 | 1 | 0 | 0 | 0 | 0 |
| TRBV30\|TRBD1\|TRBJ2-5 | 0 | 0 | 0 | 0 | 0 | 0.1629 | 0 | 0 | 0 | 0 | 0 | 2 |
| TRBV30\|TRBD1\|TRBJ2-7 | 0.2005 | 0.1834 | 0 | 0 | 0.0706 | 0 | 3 | 3 | 0 | 0 | 1 | 0 |
| TRBV30\|TRBD2\|TRBJ1-1 | 0 | 0 | 0 | 0.0931 | 0 | 0 | 0 | 0 | 0 | 1 | 0 | 0 |
| TRBV30\|TRBD2\|TRBJ2-1 | 0.1337 | 0.0611 | 0 | 0.1862 | 0 | 0.1629 | 2 | 1 | 0 | 2 | 0 | 2 |
| TRBV30\|TRBD2\|TRBJ2-2 | 0.2005 | 0.1222 | 0 | 0 | 0 | 0.1629 | 3 | 2 | 0 | 0 | 0 | 2 |
| TRBV30\|TRBD2\|TRBJ2-3 | 0.1337 | 0 | 0 | 0 | 0.1412 | 0 | 2 | 0 | 0 | 0 | 2 | 0 |
| TRBV4-1\|TRBD1\|TRBJ1-1 | 9.9599 | 0.0611 | 0 | 0 | 0.1412 | 0 | 149 | 1 | 0 | 0 | 2 | 0 |
| TRBV4-1\|TRBD1\|TRBJ1-2 | 0 | 0.0611 | 0.103 | 0 | 0.2119 | 0 | 0 | 1 | 1 | 0 | 3 | 0 |
| TRBV4-1\|TRBD1\|TRBJ1-3 | 0.2005 | 0 | 0 | 0.0931 | 0 | 0.0814 | 3 | 0 | 0 | 1 | 0 | 1 |
| TRBV4-1\|TRBD1\|TRBJ1-4 | 0.2005 | 0.1222 | 0 | 0.0931 | 0.1412 | 0.2443 | 3 | 2 | 0 | 1 | 2 | 3 |
| TRBV4-1\|TRBD1\|TRBJ1-5 | 0 | 0.1222 | 0 | 0.0931 | 0.1412 | 0.57 | 0 | 2 | 0 | 1 | 2 | 7 |
| TRBV4-1\|TRBD1\|TRBJ1-6 | 0 | 0.1834 | 0 | 0.0931 | 0 | 0 | 0 | 3 | 0 | 1 | 0 | 0 |
| TRBV4-1\|TRBD1\|TRBJ2-1 | 0 | 0 | 0.206 | 0.0931 | 0.2825 | 0 | 0 | 0 | 2 | 1 | 4 | 0 |
| TRBV4-1\|TRBD1\|TRBJ2-2 | 0 | 0 | 0 | 0.0931 | 0 | 0 | 0 | 0 | 0 | 1 | 0 | 0 |
| TRBV4-1\|TRBD1\|TRBJ2-3 | 0.0668 | 0.1834 | 0 | 0.1862 | 0 | 0 | 1 | 3 | 0 | 2 | 0 | 0 |
| TRBV4-1\|TRBD1\|TRBJ2-4 | 0.2674 | 0 | 0 | 0 | 0.1412 | 0 | 4 | 0 | 0 | 0 | 2 | 0 |
| TRBV4-1\|TRBD1\|TRBJ2-5 | 0 | 0.1222 | 0 | 0.0931 | 0 | 0.0814 | 0 | 2 | 0 | 1 | 0 | 1 |
| TRBV4-1\|TRBD1\|TRBJ2-6 | 0 | 0 | 0 | 0.1862 | 0 | 0 | 0 | 0 | 0 | 2 | 0 | 0 |
| TRBV4-1\|TRBD1\|TRBJ2-7 | 0.4679 | 0 | 0 | 0.0931 | 0.1412 | 0.1629 | 7 | 0 | 0 | 1 | 2 | 2 |
| TRBV4-1\|TRBD2\|TRBJ1-1 | 0 | 0.0611 | 0 | 0.0931 | 0.0706 | 0 | 0 | 1 | 0 | 1 | 1 | 0 |
| TRBV4-1\|TRBD2\|TRBJ1-5 | 0.1337 | 0.0611 | 0 | 0 | 0 | 0 | 2 | 1 | 0 | 0 | 0 | 0 |
| TRBV4-1\|TRBD2\|TRBJ2-1 | 0.6016 | 0.1222 | 0.103 | 0.0931 | 0.3531 | 0 | 9 | 2 | 1 | 1 | 5 | 0 |
| TRBV4-1\|TRBD2\|TRBJ2-2 | 0.0668 | 0.0611 | 0.309 | 0 | 0 | 0 | 1 | 1 | 3 | 0 | 0 | 0 |
| TRBV4-1\|TRBD2\|TRBJ2-3 | 0.4011 | 0.0611 | 0 | 0.1862 | 0.2825 | 0.1629 | 6 | 1 | 0 | 2 | 4 | 2 |
| TRBV4-1\|TRBD2\|TRBJ2-4 | 0.0668 | 0 | 0 | 0 | 0.0706 | 0 | 1 | 0 | 0 | 0 | 1 | 0 |
| TRBV4-1\|TRBD2\|TRBJ2-5 | 0.4679 | 0.1222 | 0 | 0.1862 | 0.1412 | 0.0814 | 7 | 2 | 0 | 2 | 2 | 1 |
| TRBV4-1\|TRBD2\|TRBJ2-7 | 0.4011 | 0.3667 | 0.206 | 0.2793 | 0.2825 | 0.0814 | 6 | 6 | 2 | 3 | 4 | 1 |
| TRBV4-2\|TRBD1\|TRBJ1-1 | 0 | 0.1222 | 0.206 | 0.0931 | 0 | 0.0814 | 0 | 2 | 2 | 1 | 0 | 1 |
| TRBV4-2\|TRBD1\|TRBJ1-2 | 0 | 0 | 0.103 | 0 | 0.0706 | 0 | 0 | 0 | 1 | 0 | 1 | 0 |
| TRBV4-2\|TRBD1\|TRBJ1-6 | 0 | 0.0611 | 0 | 0 | 0.0706 | 0 | 0 | 1 | 0 | 0 | 1 | 0 |
| TRBV4-2\|TRBD1\|TRBJ2-1 | 0 | 0 | 0 | 0.0931 | 0.2825 | 0 | 0 | 0 | 0 | 1 | 4 | 0 |
| TRBV4-2\|TRBD1\|TRBJ2-3 | 0 | 0 | 0 | 0 | 0 | 0.0814 | 0 | 0 | 0 | 0 | 0 | 1 |
| TRBV4-2\|TRBD1\|TRBJ2-5 | 0.2674 | 0 | 0.206 | 0.1862 | 0 | 0.0814 | 4 | 0 | 2 | 2 | 0 | 1 |
| TRBV4-2\|TRBD1\|TRBJ2-6 | 0 | 0 | 0 | 0.0931 | 0 | 0 | 0 | 0 | 0 | 1 | 0 | 0 |
| TRBV4-2\|TRBD1\|TRBJ2-7 | 0 | 0.3056 | 0 | 0.1862 | 0.0706 | 0 | 0 | 5 | 0 | 2 | 1 | 0 |
| TRBV4-2\|TRBD2\|TRBJ2-1 | 0.2005 | 0.2445 | 0 | 0.2793 | 0.1412 | 0 | 3 | 4 | 0 | 3 | 2 | 0 |
| TRBV4-2\|TRBD2\|TRBJ2-2 | 0 | 0 | 0 | 0 | 0.0706 | 0.0814 | 0 | 0 | 0 | 0 | 1 | 1 |
| TRBV4-2\|TRBD2\|TRBJ2-3 | 0.0668 | 0.3056 | 0 | 0.0931 | 0 | 0.1629 | 1 | 5 | 0 | 1 | 0 | 2 |
| TRBV4-2\|TRBD2\|TRBJ2-4 | 0 | 0.0611 | 0 | 0 | 0 | 0 | 0 | 1 | 0 | 0 | 0 | 0 |
| TRBV4-2\|TRBD2\|TRBJ2-5 | 0.0668 | 0.2445 | 0 | 0.2793 | 0.1412 | 0 | 1 | 4 | 0 | 3 | 2 | 0 |
| TRBV4-2\|TRBD2\|TRBJ2-7 | 0 | 0.7335 | 0 | 0.0931 | 0.0706 | 0.2443 | 0 | 12 | 0 | 1 | 1 | 3 |
| TRBV4-3\|TRBD1\|TRBJ2-3 | 0 | 0 | 0 | 0.0931 | 0 | 0 | 0 | 0 | 0 | 1 | 0 | 0 |
| TRBV4-3\|TRBD1\|TRBJ2-5 | 0 | 0.1834 | 0 | 0 | 0 | 0 | 0 | 3 | 0 | 0 | 0 | 0 |
| TRBV4-3\|TRBD1\|TRBJ2-7 | 0 | 0.1834 | 0 | 0 | 0 | 0 | 0 | 3 | 0 | 0 | 0 | 0 |
| TRBV4-3\|TRBD2\|TRBJ1-5 | 0 | 0.0611 | 0 | 0 | 0 | 0 | 0 | 1 | 0 | 0 | 0 | 0 |
| TRBV4-3\|TRBD2\|TRBJ2-5 | 0.0668 | 0 | 0 | 0 | 0 | 0 | 1 | 0 | 0 | 0 | 0 | 0 |
| TRBV4-3\|TRBD2\|TRBJ2-7 | 0 | 0.978 | 0 | 0 | 0 | 0 | 0 | 16 | 0 | 0 | 0 | 0 |
| TRBV5-1\|TRBD1\|TRBJ1-1 | 0.5348 | 0.4279 | 1.6478 | 0.6518 | 0.6356 | 0.4072 | 8 | 7 | 16 | 7 | 9 | 5 |
| TRBV5-1\|TRBD1\|TRBJ1-2 | 0 | 0.0611 | 0.309 | 0.1862 | 0.3531 | 0.4886 | 0 | 1 | 3 | 2 | 5 | 6 |
| TRBV5-1\|TRBD1\|TRBJ1-3 | 0.1337 | 0.0611 | 0 | 0.0931 | 0 | 0 | 2 | 1 | 0 | 1 | 0 | 0 |
| TRBV5-1\|TRBD1\|TRBJ1-4 | 0.1337 | 0 | 0.206 | 0.1862 | 0.1412 | 0.1629 | 2 | 0 | 2 | 2 | 2 | 2 |
| TRBV5-1\|TRBD1\|TRBJ1-5 | 0 | 0 | 0 | 0 | 0 | 0.1629 | 0 | 0 | 0 | 0 | 0 | 2 |
| TRBV5-1\|TRBD1\|TRBJ1-6 | 0 | 0.0611 | 0.103 | 0.0931 | 0.1412 | 0.1629 | 0 | 1 | 1 | 1 | 2 | 2 |
| TRBV5-1\|TRBD1\|TRBJ2-1 | 1.2032 | 0.489 | 1.9567 | 2.0484 | 4.5198 | 1.1401 | 18 | 8 | 19 | 22 | 64 | 14 |
| TRBV5-1\|TRBD1\|TRBJ2-2 | 0.6016 | 0.4279 | 1.2358 | 0.838 | 0.7768 | 0.1629 | 9 | 7 | 12 | 9 | 11 | 2 |
| TRBV5-1\|TRBD1\|TRBJ2-3 | 0.1337 | 0.3056 | 0.7209 | 1.3035 | 0.7768 | 0.4072 | 2 | 5 | 7 | 14 | 11 | 5 |
| TRBV5-1\|TRBD1\|TRBJ2-4 | 0.0668 | 0.1834 | 0 | 0.0931 | 0.2119 | 0.0814 | 1 | 3 | 0 | 1 | 3 | 1 |
| TRBV5-1\|TRBD1\|TRBJ2-5 | 0.6016 | 0.5501 | 1.0299 | 0.6518 | 0.3531 | 1.1401 | 9 | 9 | 10 | 7 | 5 | 14 |
| TRBV5-1\|TRBD1\|TRBJ2-6 | 0.2674 | 0.1222 | 0.206 | 0.0931 | 0.3531 | 0.4886 | 4 | 2 | 2 | 1 | 5 | 6 |
| TRBV5-1\|TRBD1\|TRBJ2-7 | 1.0027 | 0.2445 | 0.5149 | 1.4898 | 0.9887 | 0.8143 | 15 | 4 | 5 | 16 | 14 | 10 |
| TRBV5-1\|TRBD2\|TRBJ1-1 | 0.4679 | 0.1222 | 0.9269 | 0.1862 | 0.0706 | 0.0814 | 7 | 2 | 9 | 2 | 1 | 1 |
| TRBV5-1\|TRBD2\|TRBJ1-2 | 0 | 0.1222 | 0.309 | 0.0931 | 0 | 0 | 0 | 2 | 3 | 1 | 0 | 0 |
| TRBV5-1\|TRBD2\|TRBJ1-3 | 0 | 0 | 0.206 | 0 | 0 | 0 | 0 | 0 | 2 | 0 | 0 | 0 |
| TRBV5-1\|TRBD2\|TRBJ1-4 | 0.2005 | 0.0611 | 0.103 | 0 | 0 | 0.0814 | 3 | 1 | 1 | 0 | 0 | 1 |
| TRBV5-1\|TRBD2\|TRBJ1-6 | 0 | 0 | 0 | 0.0931 | 0 | 0.0814 | 0 | 0 | 0 | 1 | 0 | 1 |
| TRBV5-1\|TRBD2\|TRBJ2-1 | 2.6738 | 4.1565 | 0.9269 | 2.7933 | 2.1893 | 2.2801 | 40 | 68 | 9 | 30 | 31 | 28 |
| TRBV5-1\|TRBD2\|TRBJ2-2 | 0.7353 | 0.489 | 1.2358 | 0.1862 | 0.3531 | 0.4886 | 11 | 8 | 12 | 2 | 5 | 6 |
| TRBV5-1\|TRBD2\|TRBJ2-3 | 1.2032 | 0.6112 | 0.5149 | 1.7691 | 1.4124 | 1.3029 | 18 | 10 | 5 | 19 | 20 | 16 |
| TRBV5-1\|TRBD2\|TRBJ2-4 | 0.2005 | 0.2445 | 0 | 0.0931 | 0.2119 | 0.4072 | 3 | 4 | 0 | 1 | 3 | 5 |
| TRBV5-1\|TRBD2\|TRBJ2-5 | 1.1364 | 2.0782 | 1.2358 | 1.0242 | 0.9181 | 0.2443 | 17 | 34 | 12 | 11 | 13 | 3 |
| TRBV5-1\|TRBD2\|TRBJ2-6 | 0.1337 | 0.0611 | 0.5149 | 0.0931 | 0 | 0 | 2 | 1 | 5 | 1 | 0 | 0 |
| TRBV5-1\|TRBD2\|TRBJ2-7 | 0.869 | 2.0782 | 1.1329 | 0.5587 | 0.7768 | 2.1987 | 13 | 34 | 11 | 6 | 11 | 27 |
| TRBV5-3\|TRBD1\|TRBJ1-6 | 0 | 0 | 0 | 0.0931 | 0 | 0 | 0 | 0 | 0 | 1 | 0 | 0 |
| TRBV5-3\|TRBD1\|TRBJ2-7 | 0 | 0 | 0 | 0.0931 | 0 | 0 | 0 | 0 | 0 | 1 | 0 | 0 |
| TRBV5-3\|TRBD2\|TRBJ2-1 | 0 | 0 | 0 | 0 | 0 | 0.0814 | 0 | 0 | 0 | 0 | 0 | 1 |
| TRBV5-3\|TRBD2\|TRBJ2-5 | 0 | 0 | 0.4119 | 0 | 0 | 0 | 0 | 0 | 4 | 0 | 0 | 0 |
| TRBV5-3\|TRBD2\|TRBJ2-6 | 0 | 0 | 0 | 0.0931 | 0 | 0 | 0 | 0 | 0 | 1 | 0 | 0 |
| TRBV5-4\|TRBD1\|TRBJ1-1 | 0 | 0.0611 | 0.103 | 0 | 0 | 0.0814 | 0 | 1 | 1 | 0 | 0 | 1 |
| TRBV5-4\|TRBD1\|TRBJ1-2 | 0 | 0 | 0.103 | 0 | 0.0706 | 0.3257 | 0 | 0 | 1 | 0 | 1 | 4 |
| TRBV5-4\|TRBD1\|TRBJ1-6 | 0.0668 | 0 | 0 | 0 | 0 | 0 | 1 | 0 | 0 | 0 | 0 | 0 |
| TRBV5-4\|TRBD1\|TRBJ2-1 | 0.1337 | 0 | 0 | 0 | 0 | 0.2443 | 2 | 0 | 0 | 0 | 0 | 3 |
| TRBV5-4\|TRBD1\|TRBJ2-2 | 0.1337 | 0 | 0 | 0 | 0 | 0 | 2 | 0 | 0 | 0 | 0 | 0 |
| TRBV5-4\|TRBD1\|TRBJ2-3 | 0 | 0 | 0.103 | 0 | 0 | 0 | 0 | 0 | 1 | 0 | 0 | 0 |
| TRBV5-4\|TRBD1\|TRBJ2-4 | 0.0668 | 0.1222 | 0 | 0 | 0 | 0 | 1 | 2 | 0 | 0 | 0 | 0 |
| TRBV5-4\|TRBD1\|TRBJ2-5 | 0.0668 | 0 | 0.103 | 0.3724 | 0 | 0.0814 | 1 | 0 | 1 | 4 | 0 | 1 |
| TRBV5-4\|TRBD1\|TRBJ2-7 | 0.1337 | 0.0611 | 0 | 0.0931 | 0 | 0.1629 | 2 | 1 | 0 | 1 | 0 | 2 |
| TRBV5-4\|TRBD2\|TRBJ1-2 | 0 | 0 | 0 | 0 | 0.0706 | 0 | 0 | 0 | 0 | 0 | 1 | 0 |
| TRBV5-4\|TRBD2\|TRBJ2-1 | 0.0668 | 0.489 | 0.4119 | 0.0931 | 0.0706 | 0.57 | 1 | 8 | 4 | 1 | 1 | 7 |
| TRBV5-4\|TRBD2\|TRBJ2-2 | 0 | 0 | 0 | 0.0931 | 0.2119 | 0 | 0 | 0 | 0 | 1 | 3 | 0 |
| TRBV5-4\|TRBD2\|TRBJ2-3 | 0 | 0.3056 | 0 | 0.0931 | 0.2119 | 0.3257 | 0 | 5 | 0 | 1 | 3 | 4 |
| TRBV5-4\|TRBD2\|TRBJ2-4 | 0 | 0 | 0.206 | 0 | 0 | 0 | 0 | 0 | 2 | 0 | 0 | 0 |
| TRBV5-4\|TRBD2\|TRBJ2-5 | 0.0668 | 0.1222 | 0 | 0.0931 | 0.1412 | 0 | 1 | 2 | 0 | 1 | 2 | 0 |
| TRBV5-4\|TRBD2\|TRBJ2-7 | 0.0668 | 0.1222 | 0.206 | 0.0931 | 0 | 0.0814 | 1 | 2 | 2 | 1 | 0 | 1 |
| TRBV5-5\|TRBD1\|TRBJ1-1 | 0 | 0.0611 | 0.309 | 0 | 0 | 0.2443 | 0 | 1 | 3 | 0 | 0 | 3 |
| TRBV5-5\|TRBD1\|TRBJ1-2 | 0 | 0.1834 | 0 | 0.1862 | 0 | 0.0814 | 0 | 3 | 0 | 2 | 0 | 1 |
| TRBV5-5\|TRBD1\|TRBJ1-5 | 0 | 0 | 0 | 0 | 0.0706 | 0.2443 | 0 | 0 | 0 | 0 | 1 | 3 |
| TRBV5-5\|TRBD1\|TRBJ2-1 | 0.2674 | 0 | 0.103 | 0.0931 | 0.0706 | 0.0814 | 4 | 0 | 1 | 1 | 1 | 1 |
| TRBV5-5\|TRBD1\|TRBJ2-2 | 0.4011 | 0.1834 | 0 | 0.1862 | 0 | 0 | 6 | 3 | 0 | 2 | 0 | 0 |
| TRBV5-5\|TRBD1\|TRBJ2-3 | 0 | 0 | 0.309 | 0 | 0 | 0.0814 | 0 | 0 | 3 | 0 | 0 | 1 |
| TRBV5-5\|TRBD1\|TRBJ2-5 | 0 | 0.1222 | 0 | 0 | 0 | 0 | 0 | 2 | 0 | 0 | 0 | 0 |
| TRBV5-5\|TRBD1\|TRBJ2-6 | 0.0668 | 0 | 0 | 0 | 0 | 0 | 1 | 0 | 0 | 0 | 0 | 0 |
| TRBV5-5\|TRBD1\|TRBJ2-7 | 0.1337 | 0.1222 | 0 | 0.1862 | 0 | 0 | 2 | 2 | 0 | 2 | 0 | 0 |
| TRBV5-5\|TRBD2\|TRBJ1-1 | 0 | 0 | 0.6179 | 0 | 0 | 0 | 0 | 0 | 6 | 0 | 0 | 0 |
| TRBV5-5\|TRBD2\|TRBJ1-5 | 0 | 0 | 0 | 0.0931 | 0 | 0.0814 | 0 | 0 | 0 | 1 | 0 | 1 |
| TRBV5-5\|TRBD2\|TRBJ1-6 | 0 | 0.1222 | 0 | 0 | 0 | 0 | 0 | 2 | 0 | 0 | 0 | 0 |
| TRBV5-5\|TRBD2\|TRBJ2-1 | 0.3342 | 0 | 0.4119 | 0.1862 | 0 | 0.4886 | 5 | 0 | 4 | 2 | 0 | 6 |
| TRBV5-5\|TRBD2\|TRBJ2-2 | 0 | 0.1834 | 0 | 0 | 0.0706 | 0 | 0 | 3 | 0 | 0 | 1 | 0 |
| TRBV5-5\|TRBD2\|TRBJ2-3 | 0 | 0.3667 | 0.103 | 0 | 0.2825 | 0.1629 | 0 | 6 | 1 | 0 | 4 | 2 |
| TRBV5-5\|TRBD2\|TRBJ2-5 | 0 | 0 | 0 | 0 | 0 | 0.0814 | 0 | 0 | 0 | 0 | 0 | 1 |
| TRBV5-5\|TRBD2\|TRBJ2-7 | 0 | 0 | 0 | 0.0931 | 0.0706 | 0 | 0 | 0 | 0 | 1 | 1 | 0 |
| TRBV5-6\|TRBD1\|TRBJ1-1 | 0 | 0 | 0.6179 | 0.1862 | 0 | 0 | 0 | 0 | 6 | 2 | 0 | 0 |
| TRBV5-6\|TRBD1\|TRBJ1-2 | 0.0668 | 0 | 0 | 0 | 0 | 0 | 1 | 0 | 0 | 0 | 0 | 0 |
| TRBV5-6\|TRBD1\|TRBJ1-4 | 0 | 0 | 0 | 0.0931 | 0 | 0 | 0 | 0 | 0 | 1 | 0 | 0 |
| TRBV5-6\|TRBD1\|TRBJ1-5 | 0 | 0 | 0 | 0.0931 | 0 | 0 | 0 | 0 | 0 | 1 | 0 | 0 |
| TRBV5-6\|TRBD1\|TRBJ2-1 | 1.1364 | 0.1834 | 0 | 0.1862 | 0.1412 | 0.0814 | 17 | 3 | 0 | 2 | 2 | 1 |
| TRBV5-6\|TRBD1\|TRBJ2-2 | 0.1337 | 0 | 0 | 0.3724 | 0 | 0.0814 | 2 | 0 | 0 | 4 | 0 | 1 |
| TRBV5-6\|TRBD1\|TRBJ2-3 | 0 | 0.1834 | 0 | 0 | 0 | 0 | 0 | 3 | 0 | 0 | 0 | 0 |
| TRBV5-6\|TRBD1\|TRBJ2-5 | 0 | 0 | 0 | 0 | 0.0706 | 0 | 0 | 0 | 0 | 0 | 1 | 0 |
| TRBV5-6\|TRBD1\|TRBJ2-6 | 0.0668 | 0 | 0 | 0 | 0 | 0 | 1 | 0 | 0 | 0 | 0 | 0 |
| TRBV5-6\|TRBD1\|TRBJ2-7 | 0 | 0 | 0.309 | 0.1862 | 0.0706 | 0.0814 | 0 | 0 | 3 | 2 | 1 | 1 |
| TRBV5-6\|TRBD2\|TRBJ1-5 | 0 | 0 | 0 | 0 | 0 | 0.0814 | 0 | 0 | 0 | 0 | 0 | 1 |
| TRBV5-6\|TRBD2\|TRBJ1-6 | 0 | 0 | 0.103 | 0 | 0 | 0 | 0 | 0 | 1 | 0 | 0 | 0 |
| TRBV5-6\|TRBD2\|TRBJ2-1 | 0.5348 | 0 | 0 | 0.4655 | 0.2119 | 0 | 8 | 0 | 0 | 5 | 3 | 0 |
| TRBV5-6\|TRBD2\|TRBJ2-2 | 0 | 0.3056 | 0 | 0 | 0.0706 | 0 | 0 | 5 | 0 | 0 | 1 | 0 |
| TRBV5-6\|TRBD2\|TRBJ2-3 | 0.0668 | 0 | 0 | 0 | 0.2119 | 0.1629 | 1 | 0 | 0 | 0 | 3 | 2 |
| TRBV5-6\|TRBD2\|TRBJ2-5 | 0 | 0.1222 | 0 | 0 | 0.0706 | 0.0814 | 0 | 2 | 0 | 0 | 1 | 1 |
| TRBV5-6\|TRBD2\|TRBJ2-7 | 0.1337 | 0.1222 | 0.103 | 0.1862 | 0 | 0 | 2 | 2 | 1 | 2 | 0 | 0 |
| TRBV5-8\|TRBD1\|TRBJ1-2 | 0 | 0 | 0 | 0 | 0.0706 | 0 | 0 | 0 | 0 | 0 | 1 | 0 |
| TRBV5-8\|TRBD1\|TRBJ2-1 | 0.0668 | 0 | 0 | 0.0931 | 0 | 0.0814 | 1 | 0 | 0 | 1 | 0 | 1 |
| TRBV5-8\|TRBD1\|TRBJ2-3 | 0 | 0 | 0 | 0.0931 | 0 | 0 | 0 | 0 | 0 | 1 | 0 | 0 |
| TRBV5-8\|TRBD1\|TRBJ2-7 | 0.0668 | 0 | 0.103 | 0.0931 | 0 | 0 | 1 | 0 | 1 | 1 | 0 | 0 |
| TRBV5-8\|TRBD2\|TRBJ1-2 | 0 | 0 | 0 | 0.0931 | 0 | 0 | 0 | 0 | 0 | 1 | 0 | 0 |
| TRBV5-8\|TRBD2\|TRBJ2-1 | 0.1337 | 0 | 0 | 0.0931 | 0 | 0 | 2 | 0 | 0 | 1 | 0 | 0 |
| TRBV5-8\|TRBD2\|TRBJ2-7 | 0 | 0 | 0 | 0.0931 | 0 | 0 | 0 | 0 | 0 | 1 | 0 | 0 |
| TRBV6-1\|TRBD1\|TRBJ1-1 | 0.0668 | 0 | 0 | 0.0931 | 0.0706 | 0.0814 | 1 | 0 | 0 | 1 | 1 | 1 |
| TRBV6-1\|TRBD1\|TRBJ1-2 | 0.0668 | 0 | 0 | 0.0931 | 0.0706 | 0 | 1 | 0 | 0 | 1 | 1 | 0 |
| TRBV6-1\|TRBD1\|TRBJ1-3 | 0 | 0 | 0 | 0 | 0.0706 | 0 | 0 | 0 | 0 | 0 | 1 | 0 |
| TRBV6-1\|TRBD1\|TRBJ1-5 | 0.0668 | 0.0611 | 0.103 | 0 | 0.0706 | 0.0814 | 1 | 1 | 1 | 0 | 1 | 1 |
| TRBV6-1\|TRBD1\|TRBJ1-6 | 0 | 0 | 0.309 | 0 | 0 | 0 | 0 | 0 | 3 | 0 | 0 | 0 |
| TRBV6-1\|TRBD1\|TRBJ2-1 | 0 | 0.0611 | 0.103 | 0 | 0 | 0.0814 | 0 | 1 | 1 | 0 | 0 | 1 |
| TRBV6-1\|TRBD1\|TRBJ2-2 | 0.0668 | 0.3667 | 0 | 0.1862 | 0.0706 | 0.0814 | 1 | 6 | 0 | 2 | 1 | 1 |
| TRBV6-1\|TRBD1\|TRBJ2-3 | 0 | 0.0611 | 0.5149 | 0.0931 | 0.1412 | 0.0814 | 0 | 1 | 5 | 1 | 2 | 1 |
| TRBV6-1\|TRBD1\|TRBJ2-5 | 0 | 0.1834 | 0.206 | 0.0931 | 0.1412 | 0 | 0 | 3 | 2 | 1 | 2 | 0 |
| TRBV6-1\|TRBD1\|TRBJ2-6 | 0 | 0 | 0.309 | 0.0931 | 0 | 0 | 0 | 0 | 3 | 1 | 0 | 0 |
| TRBV6-1\|TRBD1\|TRBJ2-7 | 0.0668 | 0.0611 | 0 | 0 | 0.0706 | 0.2443 | 1 | 1 | 0 | 0 | 1 | 3 |
| TRBV6-1\|TRBD2\|TRBJ1-2 | 0 | 0 | 0 | 0.0931 | 0.0706 | 0 | 0 | 0 | 0 | 1 | 1 | 0 |
| TRBV6-1\|TRBD2\|TRBJ2-1 | 0.3342 | 0.3056 | 0 | 0.1862 | 0.0706 | 0.0814 | 5 | 5 | 0 | 2 | 1 | 1 |
| TRBV6-1\|TRBD2\|TRBJ2-2 | 0 | 0 | 0 | 0.0931 | 0 | 0 | 0 | 0 | 0 | 1 | 0 | 0 |
| TRBV6-1\|TRBD2\|TRBJ2-3 | 0 | 0.0611 | 0 | 0 | 0 | 0 | 0 | 1 | 0 | 0 | 0 | 0 |
| TRBV6-1\|TRBD2\|TRBJ2-4 | 0.0668 | 0 | 0 | 0 | 0 | 0 | 1 | 0 | 0 | 0 | 0 | 0 |
| TRBV6-1\|TRBD2\|TRBJ2-5 | 0 | 0 | 0 | 0 | 0.1412 | 0 | 0 | 0 | 0 | 0 | 2 | 0 |
| TRBV6-1\|TRBD2\|TRBJ2-6 | 0 | 0.0611 | 0 | 0 | 0 | 0 | 0 | 1 | 0 | 0 | 0 | 0 |
| TRBV6-1\|TRBD2\|TRBJ2-7 | 0.2674 | 0.0611 | 0.309 | 0 | 0.0706 | 0.1629 | 4 | 1 | 3 | 0 | 1 | 2 |
| TRBV6-2\|TRBD1\|TRBJ1-1 | 0 | 0 | 0 | 0.0931 | 0.0706 | 0 | 0 | 0 | 0 | 1 | 1 | 0 |
| TRBV6-2\|TRBD1\|TRBJ1-6 | 0 | 0.0611 | 0 | 0 | 0.0706 | 0 | 0 | 1 | 0 | 0 | 1 | 0 |
| TRBV6-2\|TRBD1\|TRBJ2-1 | 0 | 0 | 0.103 | 0.0931 | 0 | 0.1629 | 0 | 0 | 1 | 1 | 0 | 2 |
| TRBV6-2\|TRBD1\|TRBJ2-2 | 0.0668 | 0.0611 | 0 | 0 | 0.0706 | 0 | 1 | 1 | 0 | 0 | 1 | 0 |
| TRBV6-2\|TRBD1\|TRBJ2-3 | 0 | 0 | 0 | 0.1862 | 0 | 0 | 0 | 0 | 0 | 2 | 0 | 0 |
| TRBV6-2\|TRBD1\|TRBJ2-4 | 0 | 0 | 0 | 0 | 0.0706 | 0 | 0 | 0 | 0 | 0 | 1 | 0 |
| TRBV6-2\|TRBD1\|TRBJ2-7 | 0 | 0.3667 | 0 | 0.0931 | 0 | 0.0814 | 0 | 6 | 0 | 1 | 0 | 1 |
| TRBV6-2\|TRBD2\|TRBJ1-1 | 0 | 0 | 0.206 | 0 | 0.0706 | 0 | 0 | 0 | 2 | 0 | 1 | 0 |
| TRBV6-2\|TRBD2\|TRBJ1-5 | 0 | 0 | 0 | 0 | 0 | 0.0814 | 0 | 0 | 0 | 0 | 0 | 1 |
| TRBV6-2\|TRBD2\|TRBJ2-1 | 0 | 0.1222 | 0 | 0 | 0 | 0.1629 | 0 | 2 | 0 | 0 | 0 | 2 |
| TRBV6-2\|TRBD2\|TRBJ2-2 | 0 | 0.0611 | 0 | 0 | 0 | 0 | 0 | 1 | 0 | 0 | 0 | 0 |
| TRBV6-2\|TRBD2\|TRBJ2-3 | 0 | 0.1222 | 0 | 0 | 0 | 0.0814 | 0 | 2 | 0 | 0 | 0 | 1 |
| TRBV6-2\|TRBD2\|TRBJ2-5 | 0.1337 | 0.1222 | 0 | 0 | 0.0706 | 0.0814 | 2 | 2 | 0 | 0 | 1 | 1 |
| TRBV6-2\|TRBD2\|TRBJ2-7 | 0.1337 | 0 | 0.103 | 0.0931 | 0.0706 | 0.0814 | 2 | 0 | 1 | 1 | 1 | 1 |
| TRBV6-4\|TRBD1\|TRBJ2-1 | 0 | 0 | 0 | 0.4655 | 0 | 0 | 0 | 0 | 0 | 5 | 0 | 0 |
| TRBV6-4\|TRBD1\|TRBJ2-2 | 0 | 0.0611 | 0 | 0 | 0 | 0 | 0 | 1 | 0 | 0 | 0 | 0 |
| TRBV6-4\|TRBD1\|TRBJ2-3 | 0 | 0 | 0 | 0 | 0.0706 | 0 | 0 | 0 | 0 | 0 | 1 | 0 |
| TRBV6-4\|TRBD1\|TRBJ2-4 | 0 | 0 | 0 | 0 | 0.0706 | 0 | 0 | 0 | 0 | 0 | 1 | 0 |
| TRBV6-4\|TRBD2\|TRBJ2-1 | 0 | 0 | 0 | 0.1862 | 0.3531 | 0 | 0 | 0 | 0 | 2 | 5 | 0 |
| TRBV6-4\|TRBD2\|TRBJ2-2 | 0.0668 | 0 | 0 | 0.1862 | 0 | 0 | 1 | 0 | 0 | 2 | 0 | 0 |
| TRBV6-4\|TRBD2\|TRBJ2-3 | 0 | 0 | 0 | 0.0931 | 0 | 0 | 0 | 0 | 0 | 1 | 0 | 0 |
| TRBV6-4\|TRBD2\|TRBJ2-5 | 0 | 0 | 0 | 0 | 0.0706 | 0 | 0 | 0 | 0 | 0 | 1 | 0 |
| TRBV6-5\|TRBD1\|TRBJ1-1 | 0.1337 | 0.0611 | 0 | 0 | 0.2825 | 0.0814 | 2 | 1 | 0 | 0 | 4 | 1 |
| TRBV6-5\|TRBD1\|TRBJ1-2 | 0 | 0 | 0.206 | 0.0931 | 0 | 0.0814 | 0 | 0 | 2 | 1 | 0 | 1 |
| TRBV6-5\|TRBD1\|TRBJ1-3 | 0.0668 | 0 | 0 | 0 | 0 | 0 | 1 | 0 | 0 | 0 | 0 | 0 |
| TRBV6-5\|TRBD1\|TRBJ1-4 | 0 | 0.0611 | 0 | 0 | 0 | 0.0814 | 0 | 1 | 0 | 0 | 0 | 1 |
| TRBV6-5\|TRBD1\|TRBJ1-5 | 0.0668 | 0 | 0 | 0.0931 | 0 | 0.1629 | 1 | 0 | 0 | 1 | 0 | 2 |
| TRBV6-5\|TRBD1\|TRBJ1-6 | 0.1337 | 0 | 0 | 0 | 0.0706 | 0 | 2 | 0 | 0 | 0 | 1 | 0 |
| TRBV6-5\|TRBD1\|TRBJ2-1 | 0 | 0 | 0 | 0.0931 | 0.565 | 0 | 0 | 0 | 0 | 1 | 8 | 0 |
| TRBV6-5\|TRBD1\|TRBJ2-2 | 0.1337 | 0 | 0.206 | 0 | 0 | 0.57 | 2 | 0 | 2 | 0 | 0 | 7 |
| TRBV6-5\|TRBD1\|TRBJ2-3 | 0 | 0 | 0 | 0.1862 | 0.565 | 0.4886 | 0 | 0 | 0 | 2 | 8 | 6 |
| TRBV6-5\|TRBD1\|TRBJ2-4 | 0 | 0 | 0 | 0 | 0 | 0.0814 | 0 | 0 | 0 | 0 | 0 | 1 |
| TRBV6-5\|TRBD1\|TRBJ2-5 | 0.2674 | 0.2445 | 0 | 0.0931 | 0.0706 | 0 | 4 | 4 | 0 | 1 | 1 | 0 |
| TRBV6-5\|TRBD1\|TRBJ2-6 | 0.0668 | 0 | 0 | 0 | 0 | 0 | 1 | 0 | 0 | 0 | 0 | 0 |
| TRBV6-5\|TRBD1\|TRBJ2-7 | 0.1337 | 0.1222 | 0 | 0.2793 | 0.0706 | 0.0814 | 2 | 2 | 0 | 3 | 1 | 1 |
| TRBV6-5\|TRBD2\|TRBJ1-1 | 0 | 0.1222 | 0 | 0 | 0.1412 | 0 | 0 | 2 | 0 | 0 | 2 | 0 |
| TRBV6-5\|TRBD2\|TRBJ1-2 | 0 | 0 | 0 | 0 | 0.0706 | 0 | 0 | 0 | 0 | 0 | 1 | 0 |
| TRBV6-5\|TRBD2\|TRBJ1-4 | 0 | 0.0611 | 0 | 0 | 0 | 0 | 0 | 1 | 0 | 0 | 0 | 0 |
| TRBV6-5\|TRBD2\|TRBJ1-5 | 0 | 0.2445 | 0 | 0 | 0.0706 | 0 | 0 | 4 | 0 | 0 | 1 | 0 |
| TRBV6-5\|TRBD2\|TRBJ1-6 | 0.0668 | 0 | 0 | 0 | 0 | 0 | 1 | 0 | 0 | 0 | 0 | 0 |
| TRBV6-5\|TRBD2\|TRBJ2-1 | 0.2005 | 0.0611 | 0 | 0.0931 | 0.1412 | 0 | 3 | 1 | 0 | 1 | 2 | 0 |
| TRBV6-5\|TRBD2\|TRBJ2-2 | 0.0668 | 0.1834 | 0.103 | 0 | 0.0706 | 0.3257 | 1 | 3 | 1 | 0 | 1 | 4 |
| TRBV6-5\|TRBD2\|TRBJ2-3 | 0.0668 | 0.0611 | 0 | 0 | 0.0706 | 0.3257 | 1 | 1 | 0 | 0 | 1 | 4 |
| TRBV6-5\|TRBD2\|TRBJ2-5 | 0 | 0 | 0 | 0 | 0 | 0.1629 | 0 | 0 | 0 | 0 | 0 | 2 |
| TRBV6-5\|TRBD2\|TRBJ2-7 | 0.2674 | 0.0611 | 0.309 | 0.0931 | 0.0706 | 0 | 4 | 1 | 3 | 1 | 1 | 0 |
| TRBV6-6\|TRBD1\|TRBJ1-1 | 0 | 0 | 0 | 0.1862 | 0 | 0 | 0 | 0 | 0 | 2 | 0 | 0 |
| TRBV6-6\|TRBD1\|TRBJ1-2 | 0 | 0.0611 | 0 | 0 | 0.0706 | 0 | 0 | 1 | 0 | 0 | 1 | 0 |
| TRBV6-6\|TRBD1\|TRBJ1-4 | 0.0668 | 0 | 0 | 0 | 0 | 0 | 1 | 0 | 0 | 0 | 0 | 0 |
| TRBV6-6\|TRBD1\|TRBJ1-5 | 0 | 0.0611 | 0.103 | 0 | 0.2119 | 0.0814 | 0 | 1 | 1 | 0 | 3 | 1 |
| TRBV6-6\|TRBD1\|TRBJ1-6 | 0 | 0 | 0 | 0 | 0.0706 | 0 | 0 | 0 | 0 | 0 | 1 | 0 |
| TRBV6-6\|TRBD1\|TRBJ2-1 | 0.0668 | 0.3667 | 0.206 | 0.0931 | 0 | 0.1629 | 1 | 6 | 2 | 1 | 0 | 2 |
| TRBV6-6\|TRBD1\|TRBJ2-2 | 0 | 0.0611 | 0 | 0 | 0 | 0 | 0 | 1 | 0 | 0 | 0 | 0 |
| TRBV6-6\|TRBD1\|TRBJ2-3 | 0 | 0.1222 | 0 | 0.0931 | 0.0706 | 0.0814 | 0 | 2 | 0 | 1 | 1 | 1 |
| TRBV6-6\|TRBD1\|TRBJ2-4 | 0 | 0 | 0.206 | 0.0931 | 0 | 0.0814 | 0 | 0 | 2 | 1 | 0 | 1 |
| TRBV6-6\|TRBD1\|TRBJ2-5 | 0 | 0 | 0 | 0 | 0 | 0.0814 | 0 | 0 | 0 | 0 | 0 | 1 |
| TRBV6-6\|TRBD1\|TRBJ2-7 | 0.2005 | 0 | 0 | 0 | 0.2825 | 0.2443 | 3 | 0 | 0 | 0 | 4 | 3 |
| TRBV6-6\|TRBD2\|TRBJ1-1 | 0 | 0 | 0.309 | 0.0931 | 0.1412 | 0 | 0 | 0 | 3 | 1 | 2 | 0 |
| TRBV6-6\|TRBD2\|TRBJ1-5 | 0 | 0 | 0 | 0 | 0.0706 | 0 | 0 | 0 | 0 | 0 | 1 | 0 |
| TRBV6-6\|TRBD2\|TRBJ2-1 | 0 | 0.0611 | 0 | 0.0931 | 0.0706 | 0.0814 | 0 | 1 | 0 | 1 | 1 | 1 |
| TRBV6-6\|TRBD2\|TRBJ2-3 | 0.1337 | 0.0611 | 0 | 0.0931 | 0.0706 | 0.0814 | 2 | 1 | 0 | 1 | 1 | 1 |
| TRBV6-6\|TRBD2\|TRBJ2-4 | 0.0668 | 0.0611 | 0 | 0 | 0 | 0.2443 | 1 | 1 | 0 | 0 | 0 | 3 |
| TRBV6-6\|TRBD2\|TRBJ2-5 | 0 | 0.0611 | 0 | 0.0931 | 0 | 0.0814 | 0 | 1 | 0 | 1 | 0 | 1 |
| TRBV6-6\|TRBD2\|TRBJ2-6 | 0.0668 | 0 | 0 | 0 | 0 | 0 | 1 | 0 | 0 | 0 | 0 | 0 |
| TRBV6-6\|TRBD2\|TRBJ2-7 | 0 | 0 | 0.103 | 0.0931 | 0 | 0.1629 | 0 | 0 | 1 | 1 | 0 | 2 |
| TRBV6-7\|TRBD1\|TRBJ1-2 | 0 | 0 | 0 | 0 | 0.0706 | 0 | 0 | 0 | 0 | 0 | 1 | 0 |
| TRBV6-7\|TRBD1\|TRBJ1-5 | 0.0668 | 0 | 0 | 0 | 0 | 0 | 1 | 0 | 0 | 0 | 0 | 0 |
| TRBV6-8\|TRBD2\|TRBJ2-5 | 0 | 0.1834 | 0 | 0 | 0 | 0 | 0 | 3 | 0 | 0 | 0 | 0 |
| TRBV7-1\|TRBD2\|TRBJ2-1 | 0 | 0 | 0 | 0.1862 | 0 | 0 | 0 | 0 | 0 | 2 | 0 | 0 |
| TRBV7-1\|TRBD2\|TRBJ2-2P | 0 | 0.1222 | 0 | 0 | 0 | 0 | 0 | 2 | 0 | 0 | 0 | 0 |
| TRBV7-2\|TRBD1\|TRBJ1-3 | 0 | 0.0611 | 0 | 0 | 0 | 0 | 0 | 1 | 0 | 0 | 0 | 0 |
| TRBV7-2\|TRBD1\|TRBJ1-5 | 0 | 0 | 0 | 0 | 0 | 0.0814 | 0 | 0 | 0 | 0 | 0 | 1 |
| TRBV7-2\|TRBD1\|TRBJ2-1 | 0 | 0 | 0 | 0 | 0 | 0.1629 | 0 | 0 | 0 | 0 | 0 | 2 |
| TRBV7-2\|TRBD1\|TRBJ2-2 | 0 | 0.1222 | 0 | 0 | 0 | 0 | 0 | 2 | 0 | 0 | 0 | 0 |
| TRBV7-2\|TRBD1\|TRBJ2-5 | 0 | 0 | 0 | 0 | 0.0706 | 0.0814 | 0 | 0 | 0 | 0 | 1 | 1 |
| TRBV7-2\|TRBD2\|TRBJ1-2 | 0 | 0 | 0 | 0.0931 | 0 | 0 | 0 | 0 | 0 | 1 | 0 | 0 |
| TRBV7-2\|TRBD2\|TRBJ2-1 | 0.0668 | 0 | 0 | 0.0931 | 0.1412 | 0.1629 | 1 | 0 | 0 | 1 | 2 | 2 |
| TRBV7-2\|TRBD2\|TRBJ2-2 | 0 | 0 | 0 | 0 | 0.0706 | 0.0814 | 0 | 0 | 0 | 0 | 1 | 1 |
| TRBV7-2\|TRBD2\|TRBJ2-3 | 0 | 0 | 0 | 0.1862 | 0 | 0 | 0 | 0 | 0 | 2 | 0 | 0 |
| TRBV7-2\|TRBD2\|TRBJ2-7 | 0.0668 | 0 | 0.103 | 0.0931 | 0.0706 | 0.1629 | 1 | 0 | 1 | 1 | 1 | 2 |
| TRBV7-3\|TRBD1\|TRBJ1-1 | 0 | 0 | 0.4119 | 0 | 0 | 0.1629 | 0 | 0 | 4 | 0 | 0 | 2 |
| TRBV7-3\|TRBD1\|TRBJ1-2 | 0 | 0 | 0 | 0 | 0 | 0.0814 | 0 | 0 | 0 | 0 | 0 | 1 |
| TRBV7-3\|TRBD1\|TRBJ2-1 | 0 | 0 | 0 | 0.0931 | 0 | 0.0814 | 0 | 0 | 0 | 1 | 0 | 1 |
| TRBV7-3\|TRBD1\|TRBJ2-2 | 0 | 0 | 0 | 0 | 0 | 0.0814 | 0 | 0 | 0 | 0 | 0 | 1 |
| TRBV7-3\|TRBD1\|TRBJ2-3 | 0 | 0 | 0 | 0.1862 | 0.0706 | 0 | 0 | 0 | 0 | 2 | 1 | 0 |
| TRBV7-3\|TRBD1\|TRBJ2-4 | 0.0668 | 0 | 0 | 0 | 0 | 0 | 1 | 0 | 0 | 0 | 0 | 0 |
| TRBV7-3\|TRBD1\|TRBJ2-7 | 0.1337 | 0 | 0 | 0.0931 | 0 | 0 | 2 | 0 | 0 | 1 | 0 | 0 |
| TRBV7-3\|TRBD2\|TRBJ1-1 | 0 | 0 | 0 | 0.1862 | 0 | 0 | 0 | 0 | 0 | 2 | 0 | 0 |
| TRBV7-3\|TRBD2\|TRBJ1-2 | 0 | 0 | 0.309 | 0 | 0 | 0 | 0 | 0 | 3 | 0 | 0 | 0 |
| TRBV7-3\|TRBD2\|TRBJ1-5 | 0 | 0.1834 | 0 | 0 | 0 | 0 | 0 | 3 | 0 | 0 | 0 | 0 |
| TRBV7-3\|TRBD2\|TRBJ2-1 | 0 | 0 | 0 | 0 | 0.1412 | 0 | 0 | 0 | 0 | 0 | 2 | 0 |
| TRBV7-3\|TRBD2\|TRBJ2-2 | 0 | 0 | 0.103 | 0.0931 | 0.0706 | 0 | 0 | 0 | 1 | 1 | 1 | 0 |
| TRBV7-3\|TRBD2\|TRBJ2-3 | 0.0668 | 0 | 0.103 | 0 | 0 | 0 | 1 | 0 | 1 | 0 | 0 | 0 |
| TRBV7-3\|TRBD2\|TRBJ2-4 | 0.0668 | 0 | 0 | 0 | 0 | 0 | 1 | 0 | 0 | 0 | 0 | 0 |
| TRBV7-3\|TRBD2\|TRBJ2-5 | 0.0668 | 0 | 0 | 0 | 0 | 0.2443 | 1 | 0 | 0 | 0 | 0 | 3 |
| TRBV7-3\|TRBD2\|TRBJ2-7 | 0 | 0 | 0 | 0.0931 | 0 | 0 | 0 | 0 | 0 | 1 | 0 | 0 |
| TRBV7-6\|TRBD1\|TRBJ1-1 | 0 | 0 | 0 | 0 | 0.0706 | 0 | 0 | 0 | 0 | 0 | 1 | 0 |
| TRBV7-6\|TRBD1\|TRBJ1-2 | 0.0668 | 0 | 0 | 0 | 0 | 0.1629 | 1 | 0 | 0 | 0 | 0 | 2 |
| TRBV7-6\|TRBD1\|TRBJ1-4 | 0 | 0 | 0.206 | 0 | 0.1412 | 0 | 0 | 0 | 2 | 0 | 2 | 0 |
| TRBV7-6\|TRBD1\|TRBJ1-5 | 0.0668 | 0 | 0 | 0 | 0 | 0 | 1 | 0 | 0 | 0 | 0 | 0 |
| TRBV7-6\|TRBD1\|TRBJ2-1 | 0 | 0 | 0 | 0 | 0 | 0.0814 | 0 | 0 | 0 | 0 | 0 | 1 |
| TRBV7-6\|TRBD1\|TRBJ2-2 | 0 | 0 | 0 | 0 | 0.0706 | 0 | 0 | 0 | 0 | 0 | 1 | 0 |
| TRBV7-6\|TRBD1\|TRBJ2-3 | 0 | 0 | 0.4119 | 0 | 0 | 0 | 0 | 0 | 4 | 0 | 0 | 0 |
| TRBV7-6\|TRBD1\|TRBJ2-5 | 0 | 0 | 0.6179 | 0 | 0 | 0.0814 | 0 | 0 | 6 | 0 | 0 | 1 |
| TRBV7-6\|TRBD1\|TRBJ2-7 | 0 | 0 | 0 | 0 | 0.1412 | 0 | 0 | 0 | 0 | 0 | 2 | 0 |
| TRBV7-6\|TRBD2\|TRBJ1-5 | 0.1337 | 0 | 0 | 0 | 0 | 0 | 2 | 0 | 0 | 0 | 0 | 0 |
| TRBV7-6\|TRBD2\|TRBJ2-1 | 0.5348 | 0 | 0 | 0 | 0.1412 | 0 | 8 | 0 | 0 | 0 | 2 | 0 |
| TRBV7-6\|TRBD2\|TRBJ2-2 | 0 | 0.0611 | 0 | 0 | 0 | 0.1629 | 0 | 1 | 0 | 0 | 0 | 2 |
| TRBV7-6\|TRBD2\|TRBJ2-5 | 0 | 0 | 0 | 0 | 0.1412 | 0 | 0 | 0 | 0 | 0 | 2 | 0 |
| TRBV7-6\|TRBD2\|TRBJ2-7 | 0 | 0 | 0 | 0.1862 | 0 | 0 | 0 | 0 | 0 | 2 | 0 | 0 |
| TRBV7-7\|TRBD1\|TRBJ2-1 | 0 | 0.0611 | 0 | 0.0931 | 0 | 0 | 0 | 1 | 0 | 1 | 0 | 0 |
| TRBV7-7\|TRBD2\|TRBJ2-1 | 0 | 0 | 0 | 0.1862 | 0 | 0 | 0 | 0 | 0 | 2 | 0 | 0 |
| TRBV7-7\|TRBD2\|TRBJ2-5 | 0.0668 | 0 | 0 | 0.4655 | 0 | 0 | 1 | 0 | 0 | 5 | 0 | 0 |
| TRBV7-8\|TRBD1\|TRBJ1-1 | 0.0668 | 0.1834 | 0 | 0 | 0 | 0.0814 | 1 | 3 | 0 | 0 | 0 | 1 |
| TRBV7-8\|TRBD1\|TRBJ1-2 | 0 | 0 | 0 | 0 | 0.0706 | 0.0814 | 0 | 0 | 0 | 0 | 1 | 1 |
| TRBV7-8\|TRBD1\|TRBJ1-5 | 0 | 0.0611 | 0 | 0.0931 | 0 | 0.0814 | 0 | 1 | 0 | 1 | 0 | 1 |
| TRBV7-8\|TRBD1\|TRBJ1-6 | 0 | 0 | 0.309 | 0 | 0 | 0.0814 | 0 | 0 | 3 | 0 | 0 | 1 |
| TRBV7-8\|TRBD1\|TRBJ2-1 | 0.2674 | 0.0611 | 0 | 0 | 0.2119 | 0.0814 | 4 | 1 | 0 | 0 | 3 | 1 |
| TRBV7-8\|TRBD1\|TRBJ2-2 | 0 | 0 | 0 | 0 | 0.0706 | 0.0814 | 0 | 0 | 0 | 0 | 1 | 1 |
| TRBV7-8\|TRBD1\|TRBJ2-3 | 0 | 0 | 0 | 0 | 0.0706 | 0.2443 | 0 | 0 | 0 | 0 | 1 | 3 |
| TRBV7-8\|TRBD1\|TRBJ2-6 | 0.0668 | 0.0611 | 0 | 0 | 0 | 0.0814 | 1 | 1 | 0 | 0 | 0 | 1 |
| TRBV7-8\|TRBD1\|TRBJ2-7 | 0.2005 | 0 | 0 | 0.0931 | 0 | 0.0814 | 3 | 0 | 0 | 1 | 0 | 1 |
| TRBV7-8\|TRBD2\|TRBJ1-1 | 0.0668 | 0 | 0 | 0 | 0 | 0 | 1 | 0 | 0 | 0 | 0 | 0 |
| TRBV7-8\|TRBD2\|TRBJ1-2 | 0 | 0 | 0.206 | 0 | 0 | 0 | 0 | 0 | 2 | 0 | 0 | 0 |
| TRBV7-8\|TRBD2\|TRBJ2-1 | 0.0668 | 0.489 | 0 | 0 | 0.3531 | 0.4072 | 1 | 8 | 0 | 0 | 5 | 5 |
| TRBV7-8\|TRBD2\|TRBJ2-2 | 0.1337 | 0.0611 | 0 | 0.1862 | 0 | 0.1629 | 2 | 1 | 0 | 2 | 0 | 2 |
| TRBV7-8\|TRBD2\|TRBJ2-3 | 0.0668 | 0.0611 | 0.103 | 0 | 0.2119 | 0.2443 | 1 | 1 | 1 | 0 | 3 | 3 |
| TRBV7-8\|TRBD2\|TRBJ2-5 | 0.0668 | 0 | 0 | 0 | 0 | 0 | 1 | 0 | 0 | 0 | 0 | 0 |
| TRBV7-8\|TRBD2\|TRBJ2-6 | 0.0668 | 0 | 0 | 0 | 0 | 0 | 1 | 0 | 0 | 0 | 0 | 0 |
| TRBV7-8\|TRBD2\|TRBJ2-7 | 0.0668 | 0.0611 | 0 | 0 | 0.0706 | 0.0814 | 1 | 1 | 0 | 0 | 1 | 1 |
| TRBV7-9\|TRBD1\|TRBJ1-1 | 0.1337 | 0.0611 | 0.103 | 0.1862 | 0.2825 | 0.0814 | 2 | 1 | 1 | 2 | 4 | 1 |
| TRBV7-9\|TRBD1\|TRBJ1-2 | 0 | 0.0611 | 0.206 | 0.0931 | 0.2825 | 0.3257 | 0 | 1 | 2 | 1 | 4 | 4 |
| TRBV7-9\|TRBD1\|TRBJ1-3 | 0 | 0.3667 | 0 | 0 | 0 | 0 | 0 | 6 | 0 | 0 | 0 | 0 |
| TRBV7-9\|TRBD1\|TRBJ1-5 | 0 | 0 | 0.206 | 0.0931 | 0 | 0.0814 | 0 | 0 | 2 | 1 | 0 | 1 |
| TRBV7-9\|TRBD1\|TRBJ1-6 | 0 | 0 | 0.206 | 0 | 0.0706 | 0.0814 | 0 | 0 | 2 | 0 | 1 | 1 |
| TRBV7-9\|TRBD1\|TRBJ2-1 | 0.1337 | 0.1222 | 0.206 | 0.1862 | 0.6356 | 0.1629 | 2 | 2 | 2 | 2 | 9 | 2 |
| TRBV7-9\|TRBD1\|TRBJ2-2 | 0 | 0.0611 | 0 | 0.0931 | 0 | 0 | 0 | 1 | 0 | 1 | 0 | 0 |
| TRBV7-9\|TRBD1\|TRBJ2-3 | 0.0668 | 0.1834 | 0.206 | 0.1862 | 0.1412 | 0.1629 | 1 | 3 | 2 | 2 | 2 | 2 |
| TRBV7-9\|TRBD1\|TRBJ2-4 | 0 | 0 | 0 | 0.0931 | 0 | 0.0814 | 0 | 0 | 0 | 1 | 0 | 1 |
| TRBV7-9\|TRBD1\|TRBJ2-5 | 0.2674 | 0.1834 | 0.4119 | 0.2793 | 0.8475 | 0.1629 | 4 | 3 | 4 | 3 | 12 | 2 |
| TRBV7-9\|TRBD1\|TRBJ2-6 | 1.9385 | 0.0611 | 0.103 | 0 | 0 | 0 | 29 | 1 | 1 | 0 | 0 | 0 |
| TRBV7-9\|TRBD1\|TRBJ2-7 | 0.2005 | 0.6724 | 0.7209 | 0.4655 | 0.0706 | 0.0814 | 3 | 11 | 7 | 5 | 1 | 1 |
| TRBV7-9\|TRBD2\|TRBJ1-1 | 0 | 0 | 0 | 0 | 0 | 0.2443 | 0 | 0 | 0 | 0 | 0 | 3 |
| TRBV7-9\|TRBD2\|TRBJ1-2 | 0 | 0 | 0 | 0 | 0 | 0.1629 | 0 | 0 | 0 | 0 | 0 | 2 |
| TRBV7-9\|TRBD2\|TRBJ1-3 | 0 | 0 | 0 | 0 | 0.0706 | 0 | 0 | 0 | 0 | 0 | 1 | 0 |
| TRBV7-9\|TRBD2\|TRBJ1-4 | 0 | 0 | 0.206 | 0 | 0 | 0 | 0 | 0 | 2 | 0 | 0 | 0 |
| TRBV7-9\|TRBD2\|TRBJ1-5 | 0 | 0 | 0 | 0 | 0 | 0.0814 | 0 | 0 | 0 | 0 | 0 | 1 |
| TRBV7-9\|TRBD2\|TRBJ1-6 | 0.0668 | 0 | 0 | 0.0931 | 0 | 0 | 1 | 0 | 0 | 1 | 0 | 0 |
| TRBV7-9\|TRBD2\|TRBJ2-1 | 0.2005 | 0.1222 | 0 | 0.5587 | 0.1412 | 0.7329 | 3 | 2 | 0 | 6 | 2 | 9 |
| TRBV7-9\|TRBD2\|TRBJ2-2 | 0.0668 | 0.0611 | 0 | 0 | 0 | 0.0814 | 1 | 1 | 0 | 0 | 0 | 1 |
| TRBV7-9\|TRBD2\|TRBJ2-3 | 0 | 0.4279 | 0 | 0.0931 | 0.2825 | 0.1629 | 0 | 7 | 0 | 1 | 4 | 2 |
| TRBV7-9\|TRBD2\|TRBJ2-4 | 0.0668 | 0 | 0 | 0 | 0 | 0.0814 | 1 | 0 | 0 | 0 | 0 | 1 |
| TRBV7-9\|TRBD2\|TRBJ2-5 | 0.0668 | 0.1834 | 0 | 0.2793 | 0.1412 | 0 | 1 | 3 | 0 | 3 | 2 | 0 |
| TRBV7-9\|TRBD2\|TRBJ2-7 | 0.3342 | 0.6112 | 0 | 0.1862 | 0.0706 | 0 | 5 | 10 | 0 | 2 | 1 | 0 |
| TRBV9\|TRBD1\|TRBJ1-2 | 0 | 0.0611 | 0 | 0 | 0.0706 | 0 | 0 | 1 | 0 | 0 | 1 | 0 |
| TRBV9\|TRBD1\|TRBJ1-5 | 0.0668 | 0 | 0 | 0 | 0 | 0 | 1 | 0 | 0 | 0 | 0 | 0 |
| TRBV9\|TRBD1\|TRBJ1-6 | 0.5348 | 0.0611 | 0 | 0 | 0.1412 | 0 | 8 | 1 | 0 | 0 | 2 | 0 |
| TRBV9\|TRBD1\|TRBJ2-1 | 0.1337 | 0.0611 | 0.206 | 0 | 0 | 0 | 2 | 1 | 2 | 0 | 0 | 0 |
| TRBV9\|TRBD1\|TRBJ2-2 | 0 | 0.4279 | 0 | 0 | 0 | 0.0814 | 0 | 7 | 0 | 0 | 0 | 1 |
| TRBV9\|TRBD1\|TRBJ2-3 | 0.1337 | 0 | 0.309 | 0.0931 | 0 | 0.2443 | 2 | 0 | 3 | 1 | 0 | 3 |
| TRBV9\|TRBD1\|TRBJ2-5 | 0.1337 | 0 | 0.206 | 0 | 0.6356 | 0.0814 | 2 | 0 | 2 | 0 | 9 | 1 |
| TRBV9\|TRBD1\|TRBJ2-6 | 0.0668 | 0.1834 | 0 | 0 | 0 | 0 | 1 | 3 | 0 | 0 | 0 | 0 |
| TRBV9\|TRBD1\|TRBJ2-7 | 0 | 0 | 0 | 0.4655 | 0 | 0.1629 | 0 | 0 | 0 | 5 | 0 | 2 |
| TRBV9\|TRBD2\|TRBJ1-1 | 0 | 0 | 0 | 0 | 0.0706 | 0.0814 | 0 | 0 | 0 | 0 | 1 | 1 |
| TRBV9\|TRBD2\|TRBJ1-2 | 0.0668 | 0 | 0 | 0.0931 | 0 | 0 | 1 | 0 | 0 | 1 | 0 | 0 |
| TRBV9\|TRBD2\|TRBJ1-4 | 0.1337 | 0.0611 | 0 | 0 | 0 | 0 | 2 | 1 | 0 | 0 | 0 | 0 |
| TRBV9\|TRBD2\|TRBJ1-6 | 0 | 0 | 0.6179 | 0 | 0 | 0 | 0 | 0 | 6 | 0 | 0 | 0 |
| TRBV9\|TRBD2\|TRBJ2-1 | 0.1337 | 0.1834 | 0 | 0.0931 | 0.2825 | 0.1629 | 2 | 3 | 0 | 1 | 4 | 2 |
| TRBV9\|TRBD2\|TRBJ2-2 | 0.2005 | 0.2445 | 0.309 | 0.1862 | 0.0706 | 0 | 3 | 4 | 3 | 2 | 1 | 0 |
| TRBV9\|TRBD2\|TRBJ2-3 | 0.4011 | 0.0611 | 0.103 | 0 | 0.2119 | 0.1629 | 6 | 1 | 1 | 0 | 3 | 2 |
| TRBV9\|TRBD2\|TRBJ2-4 | 0 | 0 | 0 | 0 | 0.0706 | 0 | 0 | 0 | 0 | 0 | 1 | 0 |
| TRBV9\|TRBD2\|TRBJ2-5 | 0.2005 | 0 | 0 | 0 | 0 | 0.1629 | 3 | 0 | 0 | 0 | 0 | 2 |
| TRBV9\|TRBD2\|TRBJ2-7 | 0 | 0.0611 | 0 | 0 | 0.1412 | 0.0814 | 0 | 1 | 0 | 0 | 2 | 1 |
