# Supplementary figures and images for: High-throughput Treg cell receptor sequencing reveals differential immune repertoires in rheumatoid arthritis with kidney deficiency
Source: PeerJ. 2023 Feb 2;11:e14837. doi: 10.7717/peerj.14837 (PMC9899432; doi:10.7717/peerj.14837)

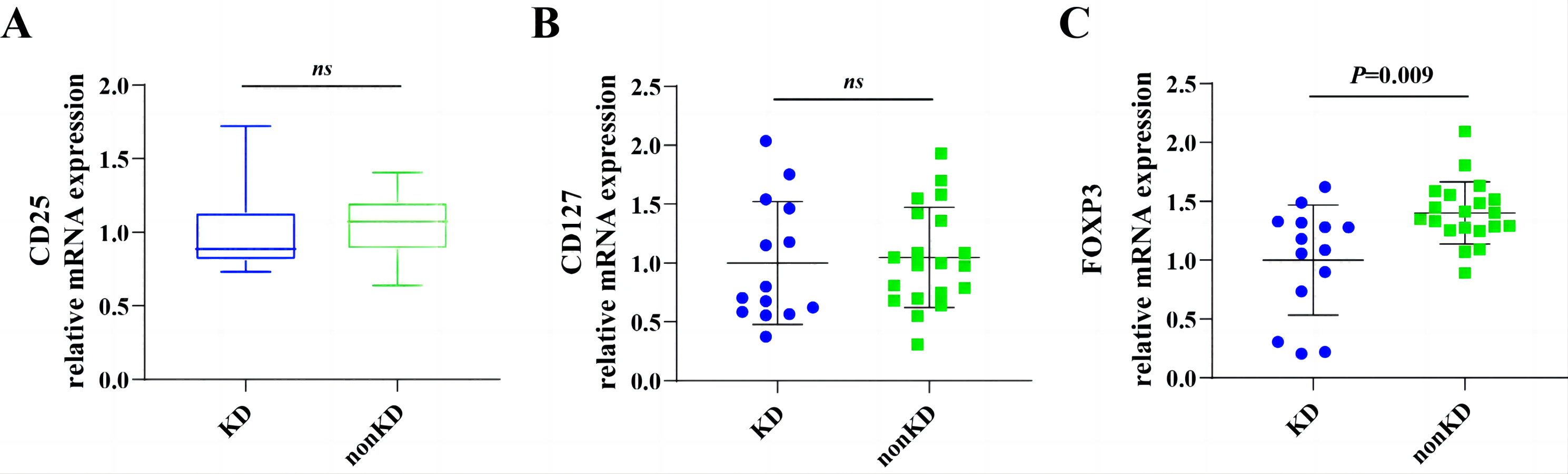

Supplement: Supplemental Information 10 — ns means no significant between two groups. [file peerj-11-14837-s010.png]
